# Lactose as a “Trojan Horse” for Quantum Dot Cell Transport**

**DOI:** 10.1002/anie.201307232

**Published:** 2013-12-05

**Authors:** David Benito-Alifonso, Shirley Tremel, Bo Hou, Harriet Lockyear, Judith Mantell, David J Fermin, Paul Verkade, Monica Berry, M Carmen Galan

**Affiliations:** School of Chemistry, University of Bristol, Cantock's CloseBristol BS8 1TS (UK); School of Physics, University of Bristol, NSQITyndall Ave, Bristol BS8 1F (UK) E-mail: mon.berry@bristol.ac.uk; Wolfson Bioimaging Facility, School of Biochemistry and Physiology & Pharmacology, Medical Sciences Building, University of Bristol, University WalkBristol BS8 1TD (UK)

**Keywords:** glycans, intracellular localization, multivalency, nanoparticles, quantum dots

## Abstract

A series of glycan-coated quantum dots were prepared to probe the effect of glycan presentation in intracellular localization in HeLa and SV40 epithelial cells. We show that glycan density mostly impacts on cell toxicity, whereas glycan type affects the cell uptake and intracellular localization. Moreover, we show that lactose can act as a “Trojan horse” on bi-functionalized QDs to help intracellular delivery of other non-internalizable glycan moieties and largely avoid the endosomal/lysosomal degradative pathway.

The ability to track functional biomolecules within the cell is essential to understanding complex cellular processes. The last few decades have seen an explosion of research in the area of nanotechnology applied to biology.^1^ Nanomaterials with novel optical, electronic, and surface properties, as well as size, geometry, distribution, and surface functionality have become useful platforms for studying biological processes.^2^ Herein, we report a simple and convenient synthesis of sugar-coated PEGylated CdSe/ZnS QDs with varying carbohydrate types and surface density that were used to study the effect of glycan type and presentation on cellular uptake and intracellular localization. We show that these biophysical parameters are highly dependent on the type of sugar coating, whereas carbohydrate surface density has an impact on toxicity to cells. Moreover, we show that lactose can be used as a “Trojan horse” on bifunctionalized QDs to help internalize sugars, such as mannose and maltotriose, that do not cross the cell membrane unaided. These bifunctionalized QDs escape the endosomal pathway and experience a different intracellular fate that is dependent on the glycan pattern and cell type.

Luminescent semiconductors, quantum dots (QDs), have emerged as a versatile class of non-isotopic detection labels suitable for live cells, in vivo imaging and immunoassays.^3^ Among the many advantages of quantum dots are their narrow emission spectra and common excitation, a photostability superior to organic fluorophores, their electron density, and their bright visible emission. QDs are inherently electron dense and therefore ideal cellular markers for correlative light electron microscopy (CLEM).^4^

Carbohydrate–lectin recognition processes are mediated by multivalent interactions that help achieve higher affinity, as well as higher specificity.^5^ Glycan-coated QDs provide a powerful tool to screen for protein–carbohydrate interactions, and consequently for the identification of carbohydrate receptors or ligands associated with intercellular recognition processes,^6^ and in our case, for a glimpse at these processes.

To use glyco-QDs effectively in biomedical applications, it is of the utmost importance to evaluate the parameters that control particle stability in physiological media and the effect that specific capping groups, that is, glycan type and glycan surface density, have on particle cellular uptake, localization, and toxicity.

Active cellular internalization is largely dependent on the inorganic core composition of the particles, size, organic shell used for glycan conjugation, and the type of glycan and cell environment (culture conditions).^7^ Functionalization with mono- and oligosaccharides has been used to facilitate cellular uptake of nanoparticles of different core composition and linker coating in a variety of cell lines.^2^–^6^, 7b,e, 8 However, little attention has been paid to the effects of glycan type and glycan surface density on cellular uptake, localization, and toxicity in the short term (hours to days).

To that end, monodispersed lipophilic CdSe/ZnS nanoparticles coated with trioctylphosphine oxide were prepared following literature procedures^9^ (see the Supporting Information for general experimental procedures) and QDs with two different core sizes (2.7±0.2 nm and 4.0±0.4 nm) were obtained. PEG-terminated dihydrolipoic acid (DHLA-PEG) linkers with a bidentate thiol motif to provide enhanced affinity for CdSe/ZnS core–shell QDs,^10^ and either a hydroxy group (as a spacer; **2**) or an acid group (for sugar attachment; **3**) were prepared (Scheme 1). Ligand exchange under reductive conditions with different ratios of HO-DHLA-PEG and HOOC-DHLA-PEG linkers **2** and **3** produced water-soluble QDs **5 a**–**d**. Similarly, QDs fully coated with mercaptoacetic acid (**1**) were prepared (MAA-QDs; **4**). The QDs were physicochemically characterized by UV/Vis, fluorescence spectroscopy and TEM (Figure S1–S3).

**Scheme 1:**
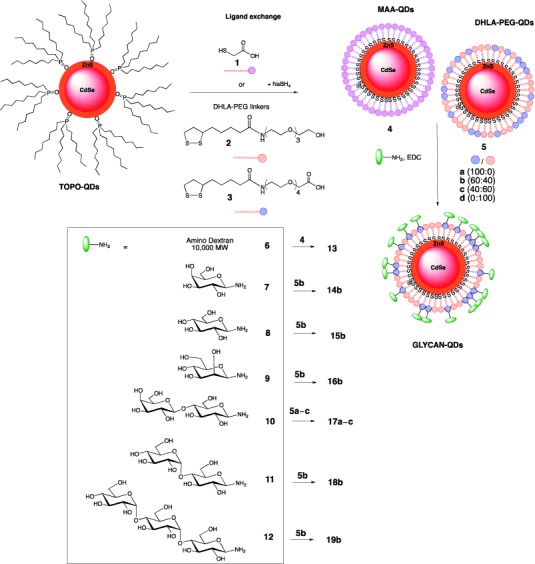
General glyco-QD preparation.

To analyze the effect of glycans and surface density, glycosylamines **7**–**12**, which were prepared by microwave-assisted Kochetkov amination,^11^ were attached to the differentially acid capped QDs **5 a**–**c** using *N*-(3-dimethylaminopropyl)-*N*′-ethylcarbodiimide hydrochloride (EDC) as the coupling reagent. Additionaly, commercial amino dextran **6** was conjugated to MAA-QDs **4** and used as a control, as fluorescent dextran conjugates have previously been used as models to study endocytosis.^12^ The corresponding glyco-QD products **13**–**19** were purified by dialysis (10 kDa membrane cutoff). The glyco-QDs were characterized as above. Additionally, their hydrodymanic volume and zeta potential^13^ were measured (Figure 1). Linker ratios and glycan incorporation onto the QDs were monitored by ^1^H NMR spectroscopy (Figure S2). All QD samples in this study were stable up to four weeks, with no particle aggregation during this period, with the exception of 100 % lactose-QDs **17 a**, which tended to aggregate in solution at room temperature.

**Figure 1:**
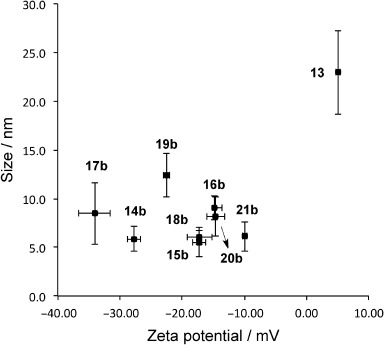
Dynamic light scattering (DLS) and Zeta potential measurements of QDs functionalized with galactose (**14 b**), glucose (**15 b**), mannose (**16 b**), lactose (**17 b**), maltose (**18 b**), maltotriose (**19 b**), lactose/mannose (**20 b**), lactose/maltotriose (**21 b**), and dextran (**13**). **14 b**–**21 b** were conjugated onto 4.0 nm QDs, **13** was conjugated onto 2.7 nm QDs.

Two cell lines, HeLa (human cervical cancer cells) and Araki Sasaki (AS, SV40-immortalized human corneal epithelium^14^) were chosen for the study. Preliminary toxicity and cell viability studies (Figure 2; see also Figures S4 and S5) were performed with dextran- (**13**) and lactose-coated (**17 a,b,c**) QDs at surface densities 100 %, 60 %, 40 %, and 0 % coated neutral-QDs **5 d**. It was found that the treatment of cells with **5 d** decreased metabolic activity compared to untreated cultures. The effects of 60 % Lactose QDs **17 b** on cell metabolism and proliferation were not different from untreated control groups after a 24 h exposure to **17 b**, suggesting that the optimum sugar density for cell uptake is found at 60 % glycan coating.

**Figure 2:**
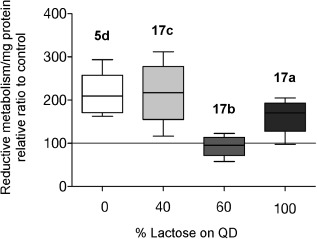
Effects of QDs (**5 d**, **17 a**–**c**) with different glycan densities on reductive metabolism in HeLa.

We then studied the effect that glycan type has on cell uptake. 60 % coated QD samples **14 b**–**19 b** were incubated in serum-containing medium (see the Supporting Information for details) with both cell lines at 37 °C for 2 and 24 h, at which time intracellular glycan-coated QDs were visualized by confocal microscopy (Figure 3). Internalization within cell organelles was determined by calculating the Manders overlap coefficient (*R*)^15^ for each organelle marker and glycan-coated QD (Figure S6).

**Figure 3:**
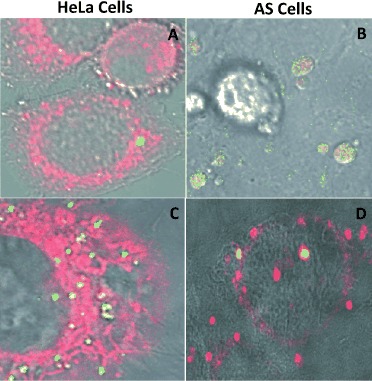
Representative confocal microscopy images showing internalization after 2 h incubation of: A) galactose-QD **14 b** in the Golgi of HeLa cells; B) **14 b** in the Lysosomes of AS cells; C) lactose-QD **17 b** in the Golgi of HeLa cells; D) **17 b** in the ER of AS cells. QD shown in green, organelle tracker in red, and overlap in yellow. For a full set of images and *R* values, see the Supporting Information.

Dextran-QDs **13** were internalized in both cell lines. After 2 h, higher uptake was observed in HeLa than AS cells. Dextran-QDs **13** were mainly localized within early and late endosomes in HeLa cells (*R*=0.48 and *R*=0.50, respectively), and mostly in early endosomes in AS cells (*R*=0.49). After incubation for 24 h with dextran-QDs **13**, fewer intracellular particles were detected in both cell lines. These results suggest that particles could be trapped in endocytic vesicles and subsequently recycled back to the plasma membrane and exocytosed, as previously observed for other nanoparticles.7c, 16 Cell division could also be attributed to the dilution in the number of dextran QDs **13** observed after 24 h incubation.^17^

Interestingly, galactose-QDs **14 b**, when taken up by both cell lines, have different intracellular accumulation sites in the two cell lines used. Galactose-QDs were mostly localized within endosomes (early (*R*=0.97) and late (*R*=0.90)) and Golgi (*R*=0.64) in HeLa cells, whereas in AS cells, colocalization with lysosomes (*R*=0.61) and early endosomes (*R*=0.66) is most conspicuous. Internalization was observed for lactose-QDs **17 b** in both cell lines, with similar intracellular localization: Lac-QDs are mostly found in endosomes, Golgi, and the ER. (Figure 3; see also Figure S8). It is noteworthy that there is a larger accumulation of **17 b** in endosomal organelles of AS cells than in HeLa cells after 2 h of incubation.

No cell uptake was detected for OH-capped QDs **5 d**, glucose **15 b**, mannose **16 b**, maltose **18 b**, or maltotriose-QDs **19 b** (data not shown). This is consistent with previously reported data where digitonin treatment of HeLa cells was necessary to cause partial damage to the plasma membrane to increase the permeability of cells as glucose- or maltotriose-CdTe QDs were not able to travel through the plasma membrane on their own.8a

On the basis that lactose QDs were internalized by the two cell lines used, we hypothesized that we could use lactose as a “Trojan horse” to help the uptake of sugars such as mannose and maltotriose by intact cells, and that the mixed conjugates could perhaps avoid the cell recycling pathway. A 1:1 mixture of aminated lactose (**10**) and mannose (**9**) were conjugated to QDs 60 % coated with COOH (**5 b**). Similarly a 1:1 mixture of **10** and maltotriose (**12**) were also conjugated to **5 b**. The resulting bifunctionalized QDs **20 b** and **21 b** were internalized by both cell lines, as observed by confocal microscopy and confirmed by correlative microscopy, that is TEM (Figure 4) and scanning transmission electron microscopy (STEM) on the cells observed in the confocal microscope. Lactose/mannose QDs **20 b** were found mainly in early endosomes in both cells lines (*R*=0.53 for AS and *R*=0.73 for HeLa), whereas Golgi accumulation was observed in HeLa cells (*R*=0.59). (Scheme 2; see also the Supporting Information) Lactose/mannose-functionalized QDs **20 b** accumulated in different compartments in the two cells lines, and underwent a different intracellular fate to that of the parent QDs **17 b** or **16 b**. Interestingly, lactose/maltotriose QDs **21 b** did not co-localize with lysosomal, ER, or mitochondrial trackers (see the Supporting Information). Confocal and correlative microscopy showed that **21 b** QDs were found mainly clustered in intracellular vesicles in the vicinity of the nucleus, albeit with cell-type specific patterns. (Scheme 2; see also the Supporting Information).

**Figure 4:**
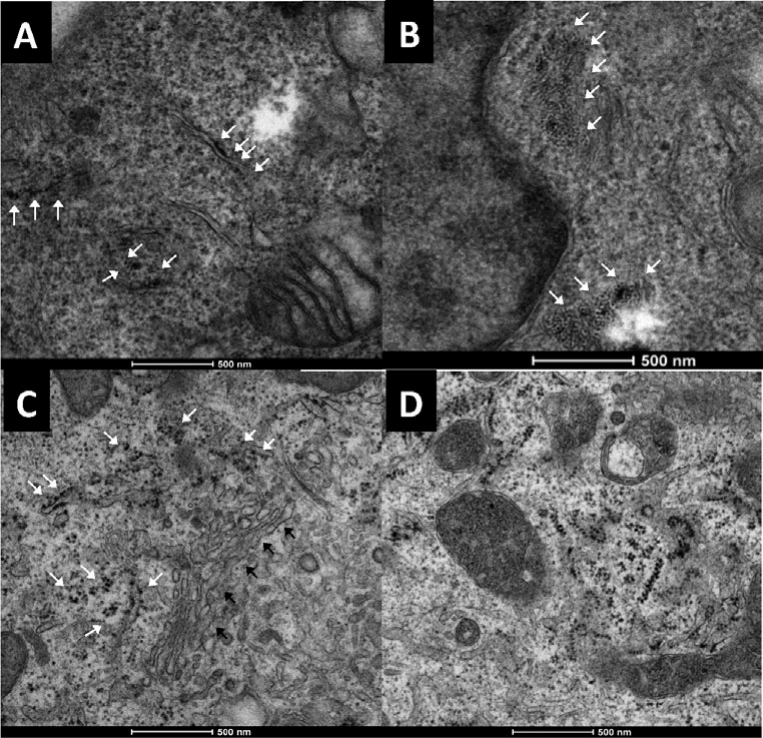
Electron microscopy images of HeLa cells: A) lactose-QDs **17 b** in the ER (white arrows); B) lactose/Maltotriose-QDs **21 b** accumulated in cytosol near the nucleus (white arrows). Electron microscopy images of AS cells: C) lactose-QDs **17 b** in the ER (white arrows) and near the Golgi (black arrows); D) **21 b** accumulating within the cytosol. All scale bars are 500 nm.

**Scheme 2:**
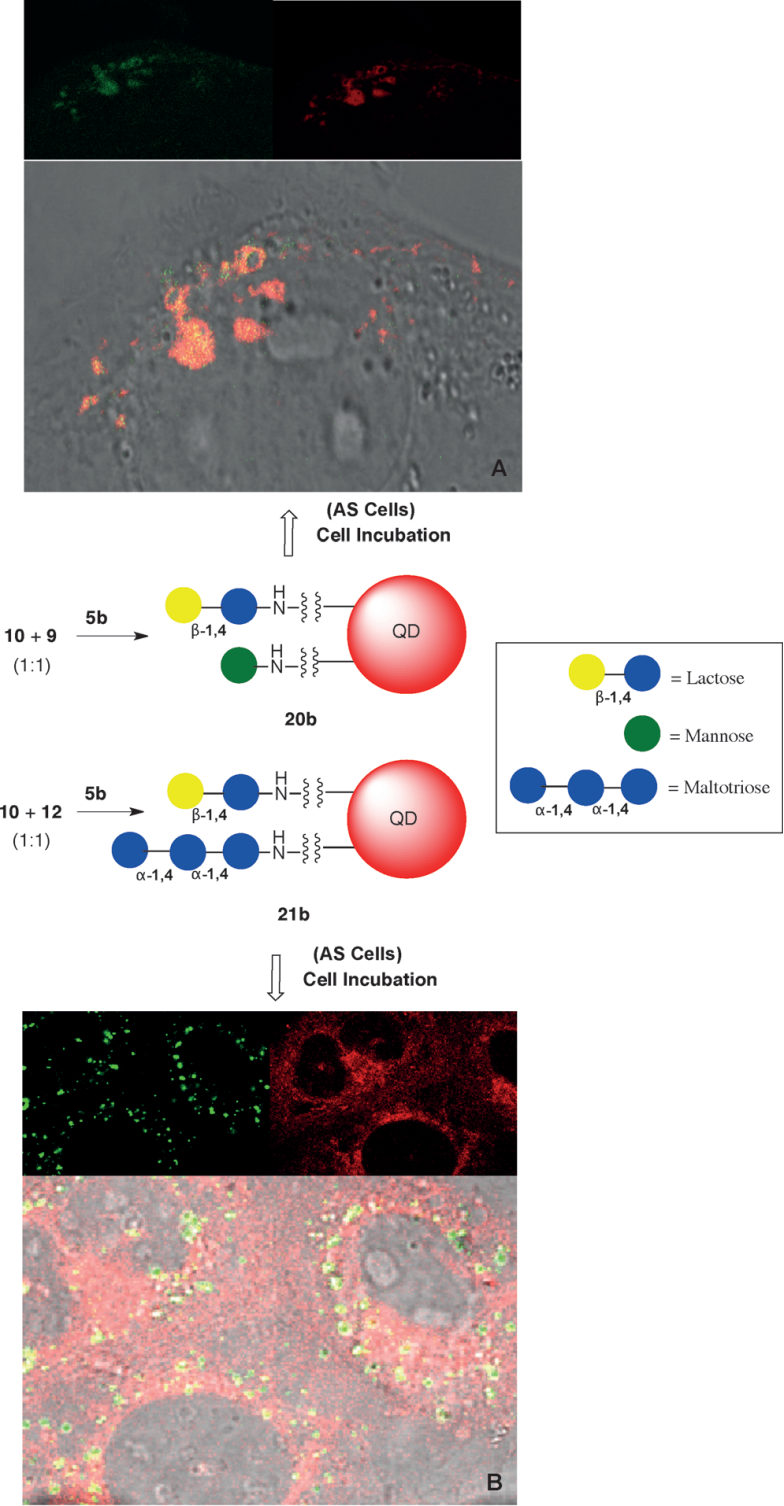
Synthesis of a 1:1 mixture of lactose/mannose-QDs **20 b** and lactose/maltotriose-QDs **21 b** and confocal microscopy images after 2 h incubation with AS cells: A) **20 b** localized in early endosomes and B) **21 b** internalization in the Golgi. QD shown in green, organelle tracker in red, and overlap in yellow.

Higher-definition pictures of QD intracellular localization obtained by CLEM and STEM4c in cells treated for 2 h with **17 b** and **21 b** (Figure 4; see also Figures S7–S10) confirmed the presence of Lactose QDs **17 b** in the ER and Golgi of both cell lines. In the case of bifunctionalized QDs **21 b**, the particles appear to have mostly circumvented the cell recycling pathway, and were found on the cytosol and Golgi of both cell lines. These results suggest a cooperative effect of lactose and maltotriose glycans that ultimately leads to particle localization within the cytoplasm.

In conclusion, we have shown that different types of glycan modulate nanoparticle uptake and intracellular localization, whereas glycan density protects from core and linker coating overt toxicity. For each cell type, the uptake mechanism of a given nanoparticle is still not fully understood^18^ and factors such as particle size^19^ and linker coating,7b as well as cell culture media (for example, the presence or absence of a protein corona)7f,g are implicated in QD uptake. Using the same core, conjugating linker, and cell culture medium, we demonstrated that QD intracellular localization can be modulated by the surface carbohydrates that decorate it. This observation was exploited to internalize mannose and maltotriose on lactose/mannose and lactose/maltotriose bifunctional QDs. These QDs had different intracellular fates, dependening on glycan combination and cell line. Interestingly, the lactose/maltotriose bifunctionalized-QDs were found in the cytosol and in perinuclear vesicles, in addition to the Golgi; this suggests *endo*-lysosomal escape, which might be physiological or a subtle sign of nano-toxicity. CLEM and STEM further indicated that intracellular accumulation of the lactose/maltotriose-QDs was altered in comparison to lactose-QDs. The alteration in the intracellular fate of these bifunctionalized QDs might point to a high-glycan specificity of the complex mechanisms that regulate vesicle transport and vesicle fusion.^20^ Further studies are currently underway to better understand the glycan influence in cellular uptake and localization of these nanoparticles. Our results suggest new opportunities to utilize the inherent glycan diversity as a strategy for the intracellular-targeted therapeutic delivery of biomolecules.

## Supporting Information

As a service to our authors and readers, this journal provides supporting information supplied by the authors. Such materials are peer reviewed and may be re-organized for online delivery, but are not copy-edited or typeset. Technical support issues arising from supporting information (other than missing files) should be addressed to the authors.

miscellaneous_information
