# Supplementary material for: Lactose as a “Trojan Horse” for Quantum Dot Cell Transport
Source: Angew Chem Int Ed Engl. 2013 Dec 5;53(3):810–4. doi: 10.1002/anie.201307232 (PMC4227560; doi:10.1002/anie.201307232)
Supplement: Supplementary file 1 — miscellaneous_information [file anie0053-0810-SD1.pdf]

Angewandte Chemie
Eine Zeitschrift der Gesellschaft Deutscher Chemiker

Supporting Information
© Wiley-VCH 2013
69451 Weinheim, Germany

Lactose as a "Trojan Horse" for Quantum Dot Cell Transport**

David Benito-Alifonso, Shirley Tremel, Bo Hou, Harriet Lockyear, Judith Mantell, David J. Fermin, Paul Verkade, Monica Berry, and M. Carmen Galan**

anie_201307232_sm_miscellaneous_information.pdf

Electronic Supplementary Information

Content:

General experimental procedures	S2
Synthesis of linkers	S2
Synthesis of CdSe Quantum Dots (QDs)	S5
QD linker functionalization and ^{1}H-NMR characterization	S6
^{1}H-NMR characterization of aminated glycans	S7
QD glycan functionalization and ^{1}H-NMR characterization	S8
QD physico-chemical characterization	S10
Cell cultures and toxicity assays	S12
Confocal microscopy experimental details	S12
Colocalization analysis	S14
CLEM and STEM experimental protocols and images	S15
References	S20
Confocal images and colocalization data	S21

Experimental Procedures

General. Chemicals were purchased and used without further purification. Dry solvents were obtained by distillation using standard procedures, or by passage through a column of anhydrous alumina using equipment from Anhydrous Engineering (University of Bristol) based on the Grubbs' design. Reactions requiring anhydrous conditions were performed under N_2; glassware and needles were either flame dried immediately prior to use, or placed in an oven (150 °C) for at least 2 h and allowed to cool in a desiccators or under reduced pressure.Liquid reagents, solutions or solvents were added *via* syringe through rubber septa; solid reagents were added *via* Schlenk type adapters. Teflon rings were used between the joints of the condensers and round bottom flasks. Reactions were monitored by TLC on Kieselgel 60 F254 (Merck), with UV light (254 nm) detection and by charring with 10% sulfuric acid in ethanol. Flash column chromatography was performed using silica gel [Merck, 230−400 mesh (40−63 μm)]. Extracts were concentrated *in vacuo* using both a Büchi rotary evaporator (bath temperatures up to 40 °C) at a pressure of 15 mmHg (diaphragm pump) or 0.1 mmHg (oil pump), as appropriate, and a high vacuum line at room temperature. Water soluble compounds were freeze dried on a Lytotrap Plus (LTE Scientific LTD). ^{1}H NMR and ^{13}C NMR spectra were measured in the solvent stated at 400, 500 or 600 MHz. Chemical shifts are quoted in parts per million from residual solvent peak ($CDCl_3$: ^{1}H - 7.26 ppm and ^{13}C - 77.16 ppm) and coupling constants (*J*) given in Hertz. Multiplicities are abbreviated as: b (broad), s (singlet), d (doublet), t (triplet), q (quartet), m (multiplet) or combinations thereof. Dynamic Light Scattering (DLS) and Zeta analysis are carried out using Malvern Instruments, Nano_S90 Red Laser Model ZEN1690 for DLS and Nano-Z ZEN 2600 for Zeta potential.

Chemical Synthesis

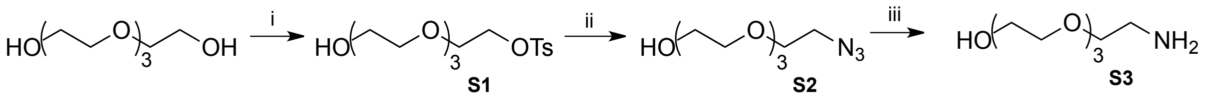

Scheme S1. Reagents and conditions: (i) TsCl, Et_3N; (ii) NaN3; (iii) H_2, Pd/C, HCl

2-(2-(2-(2-Hydroxyethoxy)ethoxy)ethoxy)ethyl 4-methylbenzenesulfonate (S1).[1] To a stirred solution of tetraethyleneglycol (8.0 g, 41 mmol) in CH_2Cl_2 (82 mL), Et_3N (5.7 mL, 41 mmol) and tosyl chloride (3.9 g, 21 mmol) were added, and the solution stirred at room temperature for 24 h. The mixture was then washed with H_2O (3 x 30 mL), brine (1 x 30 mL), dried over $MgSO_4$, filtered and concentrated *in vacuo* to afford **S1** as a clear oil (8.6 g, 60%). ^{1}H NMR (400 MHz, $CDCl_3$) d 7.82-7.79 (m, 2H, Ar), 7.37-7.34 (m, 2H, Ar), 4.18-4.10 (m, 2H, CH_2OTs), 3.74-3.56 (m, 14H, 7×CH_2), 2.46 (s, 3H, CH_3); ^{13}C NMR (100 MHz, $CDCl_3$) d 145.0 (Ar), 133.1 (Ar), 130.0 (Ar), 128.08 (Ar), 128.07 (Ar), 72.6, 70.83, 70.81, 70.75, 70.64, 70.56, 69.4 (7×CH_2), 68.8 (CH_2OTs), 61.8 (CH_2OH), 21.8 (CH_3).

2-(2-(2-(2-azidoethoxy)ethoxy)ethoxy)ethanol (S2).[1] To a stirred solution of **S1** (4.85 g, 13.9 mmol) in a mixture of H_2O/acetone (1/1, v/v, 70 mL), NaN_3 (4.52 g, 69.7 mmol) and NaI (0.21 g, 1.4 mmol) were added portionwise and the mixture refluxed for 17 h. The acetone was then removed under reduced pressure and the remaining aqueous phase was extracted with CH_2Cl_2 (3 x 30mL), brine (1 x 30 mL), dried over $MgSO_4$, filtered and concentrated *in vacuo* to afford **S2** as a clear oil (2.4 g, 80%). ^{1}H NMR (400 MHz, $CDCl_3$) d 3.61-3.54 (m, 12H, 6×CH_2), 3.49-3.47 (m, 2H, CH_2OH), 3.27 (t, 2H, *J* = 5.1 Hz, CH_2N_3); ^{13}C NMR (100 MHz, $CDCl_3$) d 72.4, 70.54, 70.49, 70.4, 70.2, 69.9 (6×CH_2), 61.5 (CH_2OH), 50.5 (CH_2N_3).

2-(2-(2-(2-aminoethoxy)ethoxy)ethoxy)ethanol (S3).[1] To a stirred solution of **S2** (2.4 g, 11 mmol) in a mixture of EtOH:HCl (95/5, v/v, 20 mL), Pd/C catalyst (10 mol%, 2.5 g) was added. The mixture was put under a N_2 atmosphere and hydrogen was then bubbled through the solution for 6 h. Upon completion of the reaction, as indicated by TLC, the solution was filtered through Celite™ and concentrated *in vacuo* to afford **S3** as an brown oil (2.5 g, 97%). ^{1}H NMR (400 MHz, $CDCl_3$) d 3.67-3.61 (m, 12H, 6×CH_2), 3.41-3.38 (m, 2H, CH_2OH), 2.88 (m, 2H, CH_2NH_2); ^{13}C NMR (100 MHz, $CDCl_3$) d 70.8, 70.6, 70.53, 70.48, 70.46, 70.1 (6×CH_2), 54.7 (CH_2OH), 50.8 (CH_2NH_2).

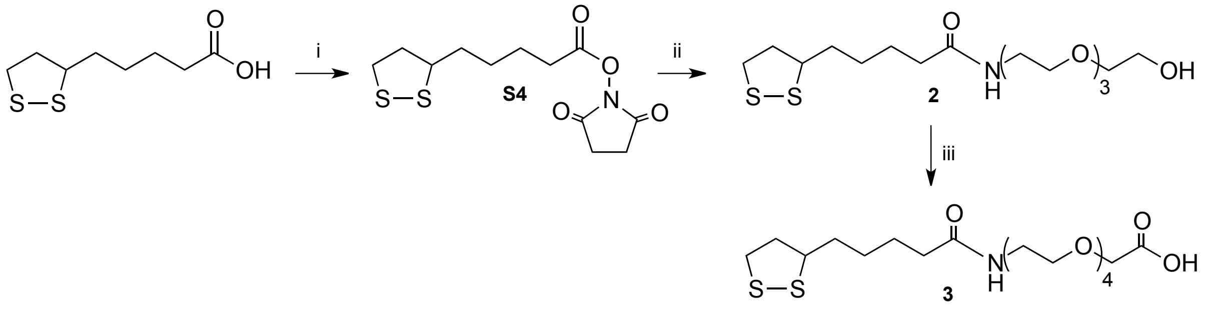

Scheme S2. Reagents and conditions: (i) NHS, DCC ,THF; (ii) S**3**, DMF/H_2O; (iii) NaH, TBAI, DMF, 0°C.

Lipoic Acid NHS-Ester (LA-NHS) (S4).[2]

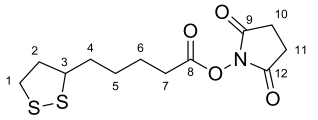

To a stirred solution of lipoic acid (3.0 g, 15 mmol) in THF (75mL), N-hydroxysuccinimide (2.0 g, 17 mmol) was added, and the mixture was cooled to 4 °C. A solution of dicyclohexylcarbodiimide (3.6 g, 17 mmol) in THF (12 mL) was then added dropwise, and the reaction mixture was allowed to stir at room temperature overnight. The solution was then filtered through Celite™ and concentrated *in vacuo* to give **S4** as a yellow solid (4.1 g, 94%). ^{1}H NMR (400 MHz, $CDCl_3$) δ 3.61-3.54 (m, 1H, H-3), 3.21-3.08 (m, 2H, 2×H-1), 2.83 (s, 4H, 2×H-10, 2×H-11), 2.62 (t, 2H, *J* = 7.3 Hz, 2×H-7) 2.50-2.42 (m, 1H, H-2_a), 1.96-1.88 (m, 1H, H-2_b), 1.82-1.75 (m, 2H, 2×H-4), 1.74 (m, 2H, 2×H-6), 1.60-

1.51 (m, 2H, 2×H-5); 13 C NMR (100 MHz, CDCl3) δ 169.1 (C-9, C-12), 168.4 (C-8), 56.1 (C-3), 40.1 (C-2), 38.5 (C-1), 34.4 (C-4), 30.7 (C-7), 28.3 (C-5), 25.6 (C-8, C-9), 24.4 (C-6).

Lipoic Acid-PEG-OH (2).

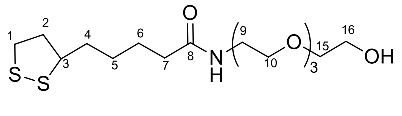

A solution of **S3** (1.79 g, 5.92 mmol) was neutralised in aqueous $NaHCO_3$ (1 M, 28 mL) and the volume of the solution was reduced to 10 mL *in vacuo*. The solution was cooled to 4 °C and to it a solution of **S4** (2.66 g, 9.23 mmol) was added dropwise, over 90 minutes. The reaction was then warmed to 20 °C and left to stir for 24 hours. The product was extracted with $CHCl_3$ (3 x 40 mL) and dried over $MgSO_4$. The product was evaporated to dryness and purified by flash silica gel chromatography (CH_2Cl_2/MeOH gradient: 1/0 to 9/1, v/v) to give **2** as a yellow oil (1.1 g, 70%). ^{1}H NMR (400 MHz, $CDCl_3$) d 7.09 (s, 1H, NH), 3.75 – 3.49 (m, 15H, 6×OCH_2, H-3), 3.52 – 3.44 (m, 2H, 2×H-9), 3.26 – 3.08 (m, 2H, 2×H-1), 2.49 – 2.52 (m, 1H, H-2$_a$), 2.18 (t, *J* = 7.9 Hz, 2H, 2×H-7), 1.94 – 1.86 (m, 1, H-2$_b$), 1.80 – 1.62 (m, 4H, 2×H-4, 2×H-6), 1.57-1.41 (m, 2H, 2×H-5); ^{13}C NMR (100 MHz, $CDCl_3$) d173.0 (C-8), 72.6, 70.6, 70.41, 70.35, 70.0 (6 x OCH_2), 61.5 (C-16), 56.5 (C-3), 40.2 (C-2), 39.1 (C-9), 38.4 (C-1), 36.2 (C-7), 34.7 (C-4), 29.0 (C-5), 25.4 (C-6); ESI-HRMS for $C_{16}H_{31}NO_5NaS_2$ (MNa^+) Calculated: 404.1536; Found: 404.1528.

Lipoic Acid-PEG-COOH (3).[3]

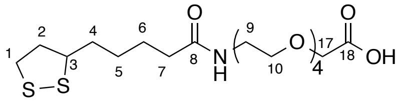

Bromoacetic acid (0.11 g, 0.79 mmol) and TBAI (10 mol%) were added to a solution of **2** (0.1 g, 0.26 mmol) in DMF (2 mL) under a nitrogen atmosphere and the mixture cooled to 0°C. Sodium hydride (31 mg, 1.3 mmol) was then added and the mixture stirred at 0°C for 30 minutes before being allowed to warm to room temperature and stirred for 5 hours. The reaction was quenched using ammonium chloride solution and the solvent removed. The residue was then dissolved in chloroform (10 mL) and washed with brine (3 x 10 mL). The crude product was purified by flash column chromoatography (gradient: DCM/MeOH: 1:0 to 9:1, v/v) to give **3** as a yellow syrup (41.6 mg, 0.09 mmol, 37%). ^{1}H NMR (400 MHz, CDCl3) δ 4.14 (s, 2H, 2×H-17), 3.73-3.53 8 (m, 15H, 7×OCH2, H-3), 3.55 (t, 2H, J = 5.5 Hz, 2×H-9), 3.21-3.03 (m, 2H, 2×H-1), 2.51-2.43 (m, 1H, H-2$_a$), 2.22 (t, 2H, J = 7.4 Hz, 2×H-7), 1.94-1.86 (m, 1H, H-2$_b$),

1.75-1.60 (m, 4H, 2×H-6, 2×H-4), 1.51-1.38 (m, 2H, 2×H-5); 13 C NMR (100 MHz, CDCl3) δ 172.9 (C-18), 169.4 (C-8), 72.3, 70.1, 69.9, 69.7, 69.6, 69.6, 69.5 (7×OCH_2), 56.1 (C-3), 39.8 (C-2), 38.7 (C-9), 38.0 (C-1), 35.5 (C-7), 34.2 (C-4), 28.4 (C-5),25.1 (C-6). $C_{18}H_{33}NO_7NaS_2$ (MNa^+) Calculated: 462.1591; Found: 462.1585.

General Procedure for Synthesis of CdSe QDs. Tributylphsophine (TBP) (5 mL) was syringed into a vessel containing elemental selenium (Se) (39.5 mg, 0.50 mmol) under argon to give a 0.1M Se solution. Cadmium oxide (37 mg, 0.30 mmol), tetradecylphosphonic acid (TDPA) (334 mg, 1.2 mmol) and trioctylphosphine oxide (TOPO) (3.6g, mmol) were dried *in vacuo* at 40 °C for 15 min and then at 60 °C for 15 min. The atmosphere was exchanged for Ar and the solution heated to 300 °C. When the solution turned colourless the Se solution (2.5 mL, 0.25 mmol) was added. After 1-3 min*, the reaction was quenched by rapid cooling. Particles were precipitated by addition of MeOH (10 mL), centrifuged (4 min, 6000 rpm) and the supernatant discarded. The particles were then redissolved in $CHCl_3$ (10 mL). (***Note:** By varying the duration, different size QDs, i.e. different colors were obtained – with yellow-green for shorter times and orange-red for longer times)

General Procedure for ZnS Coating of CdSe QDs. CdSe QD solution (8 mL, 0.2 mmol) was added to TOPO (3.6g, 1 mmol) and the $CHCl_3$ evaporated under vacuum at 50 °C. The resultant material was then heated to 180 °C under Ar. In a separate flask, to a degassed (Ar for 30 min) solution of zinc stearate (198 mg, 0.313 mmol) in toluene (5 mL), was added hexamethyldisilathiane (0.05 mL, 0.2 mmol). The resulting ZnS solution (0.7 mL, 0.03 mmol) was added dropwise to the heated CdSe QD solution and mixed for 2 h at 100 °C. The mixture was then allowed to cool and the particles precipitated by the addition of MeOH (10 mL). The mixture was centrifuged (4 min, 6000 rpm) and the supernatant discarded before redissolving the Cdse/ZnS QDs in $CHCl_3$.

General Procedure for Ligand Exchange. To a flask containing a solution of the **Linker** (0.29 mmol) in a mixture of MeOH/H_2O (1/1, v/v, 3 mL) was added $NaBH_4$ (21.9 mg, 0.58 mmol) under a N_2 atmosphere and the mixture stirred for 1 h. The pH of the solution was then adjusted to neutral by the dropwise addition of HCl (1 M) before transferring it to a glass vial. The CdSe/ZnS QD solution in $CHCl_3$ (2 mL) was then added to the vial and the biphasic mixture stirred vigorously for 16 h. Once the coloured QDs had transferred to the aqueous layer, the $CHCl_3$ phase was removed and the aqueous layer dialyzed for 16 h to before the QDs solution was freeze dryed.

NMR spectra of Lipoic Acid-PEG- coated QDs.

A) Compound **5a** (**Linker** = 100 % of **3**)
B) Compound **5b** (**Linker** = 60 % of **3** and 40 % of **2**)
C) Compound **5c** (**Linker** = 40 % of **3** and 60 % of **2**)
D) Compound **5d** (**Linker** = 100% of **2**)

A) ^{1}H NMR spectra of compound **5a**

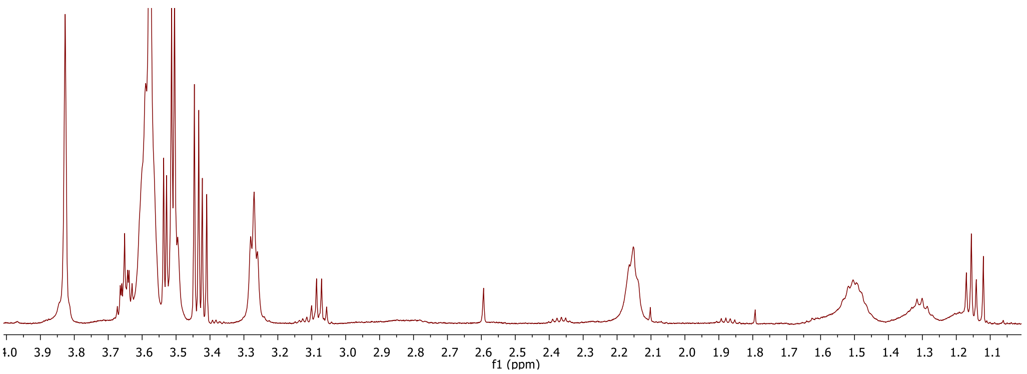

B) ^{1}H NMR spectra of compound **5b**

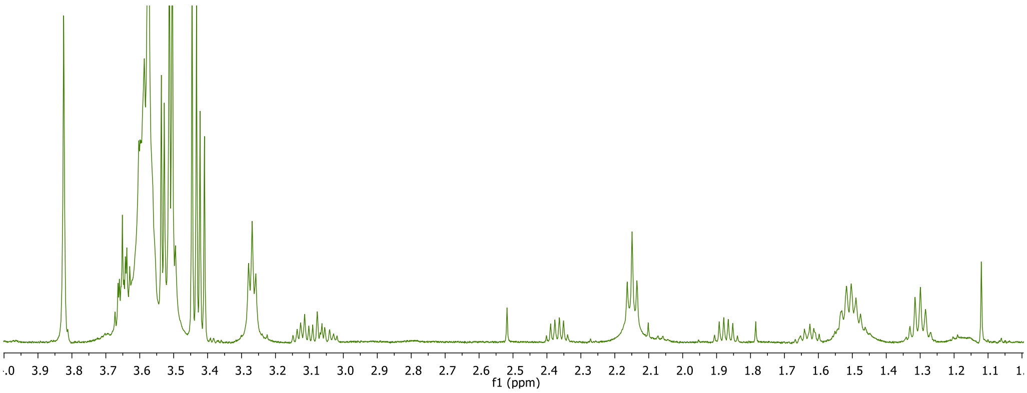

C) ^{1}H NMR spectra of compound **5c**

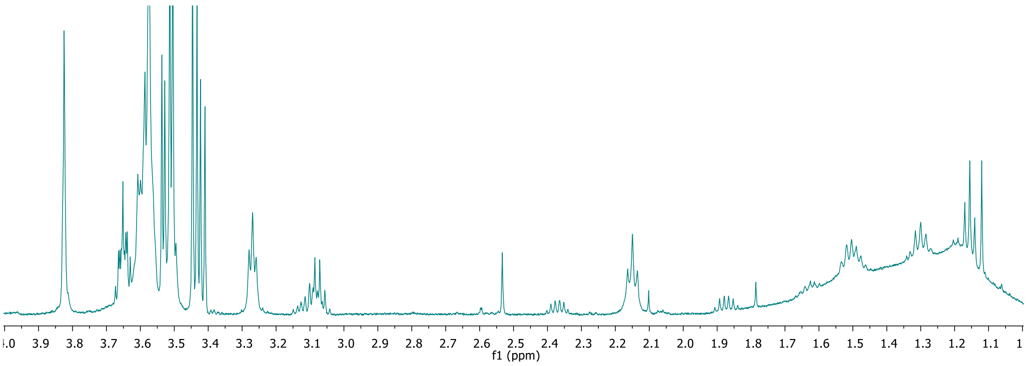

D) ^{1}H NMR spectra of compound **5d**

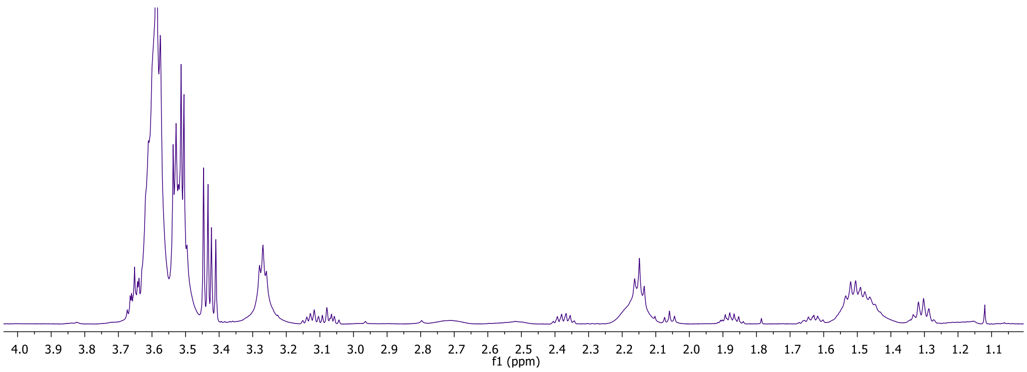

General Procedure for Functionalisation with Aminated Carbohydrates. Glycosylamine carbohydrates were prepared using the microwave assisted Kochetkov amination protocol of the corresponding unprotected glycosides with ammonium carbonate (5 fold excess w/w over sugar).[4,5] Water soluble QDs (**4** and **5**) were added to a PBS buffer solution (pH 7.4, 3 mL) and 1 mL transferred to a glass vial. **Glycosylamine** (0.04 mmol) and EDC (34 mg, 0.22 mmol) were then added and the mixture stirred vigorously for 18 h at room temperature. The QD solution was then dialysed against water for 16 h and the QDs collected and freeze dried.

β-galactosylamine

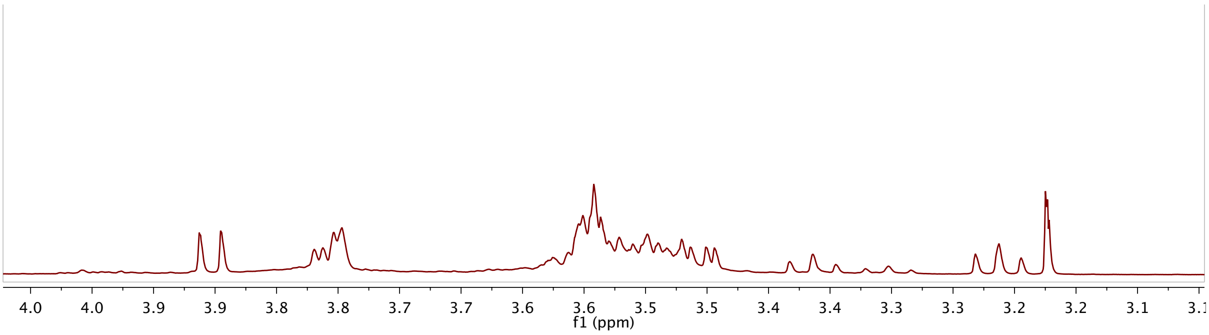

^{1}H NMR (500 MHz, D_2O) δ 3.90 (d, 1H, J = 8.7 Hz, H-1) ppm

β-lactosylamine

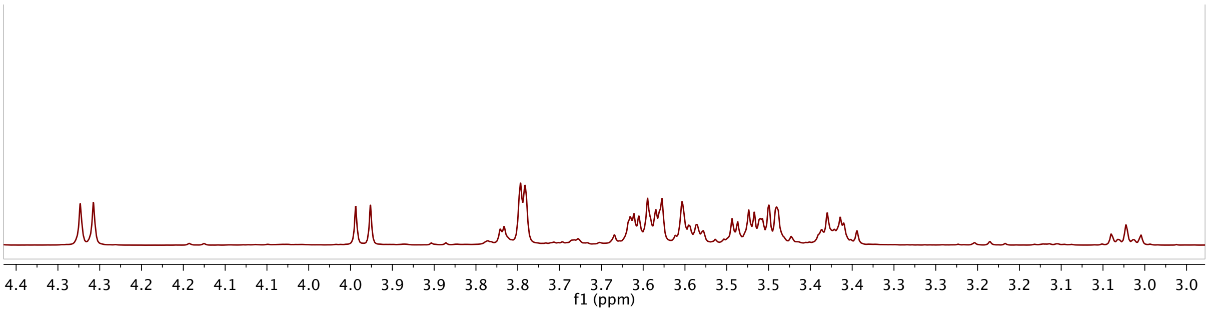

^{1}H NMR (500 MHz, D_2O) δ 4.32 (d, 1H, J = 7.8 Hz, H-1'), 3.99 (d, 1H, J = 8.8 Hz, H-1) ppm

β-maltotriosylamine

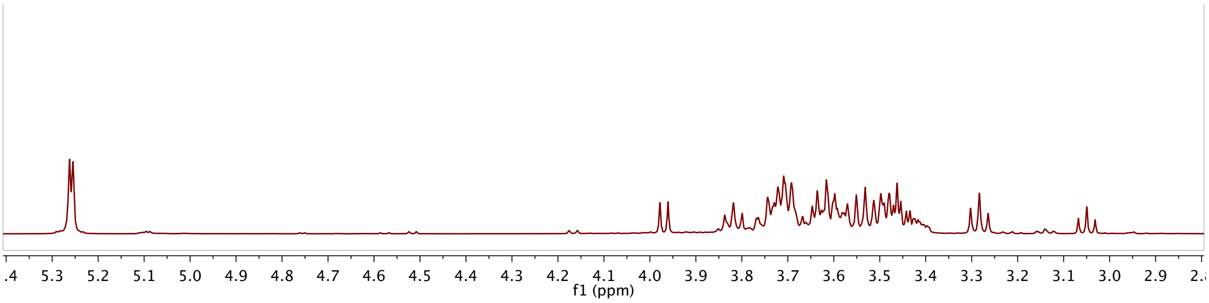

^{1}H NMR (500 MHz, D_2O) δ 5.27 (d, 2H, J = 3.8 Hz, H-1', H-1''), 3.98 (d, 1H, J = 8.8 Hz, H-1) ppm

β-mannosamine

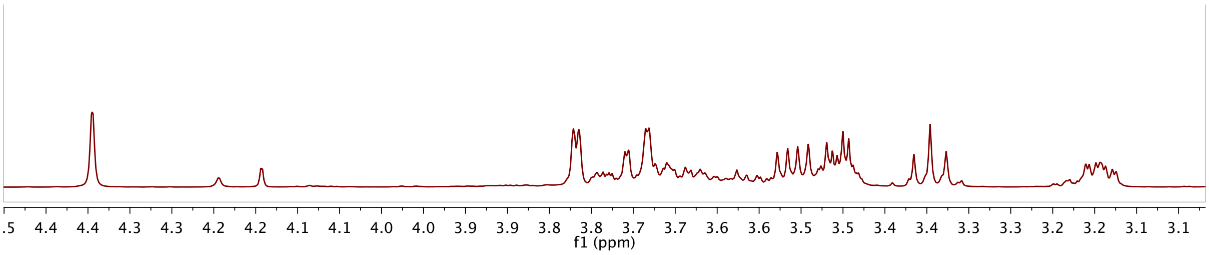

^{1}H NMR (500 MHz, D_2O) δ 4.40 (bs, H-1) ppm

NMR spectra of carbodydrate coated QDs

A) Compound **13** (**Glycosylamine** = Dextran amine 10,000 MW (Invitrogen) conjugated onto **4**)
B) Compound **14b** (**Glycosylamine** = β-galactosylamine conjugated onto **5b**)
C) Compound **18b** (**Glycosylamine** = β-lactosylamine conjugated onto **5b**)
D) Compound **20b** (**Glycosylamine** = β-lactosylamine/β-mannosamine (1/1) conjugated onto **5b**)
E) Compound **21b** (**Glycosylamine** = β-lactosylamine/β-maltotriosylamine (1/1)conjugated onto **5b**)

A) ^{1}H NMR spectra of compound Dextran-QD **13**

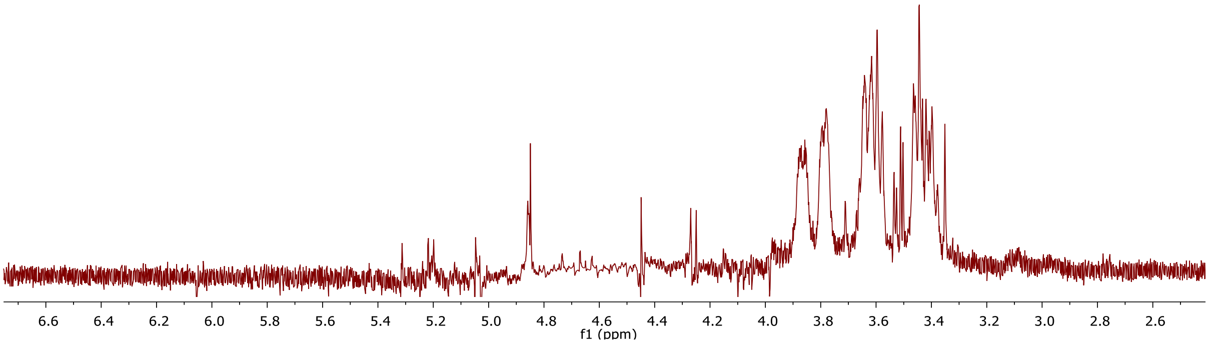

^{1}H NMR (500 MHz, D_2O) anomeric peaks: δ 5.21, 4.26 ppm (H_2O peak suppressed at 4.65 ppm)

B) ^{1}H NMR spectra of compound Galactose **14b**

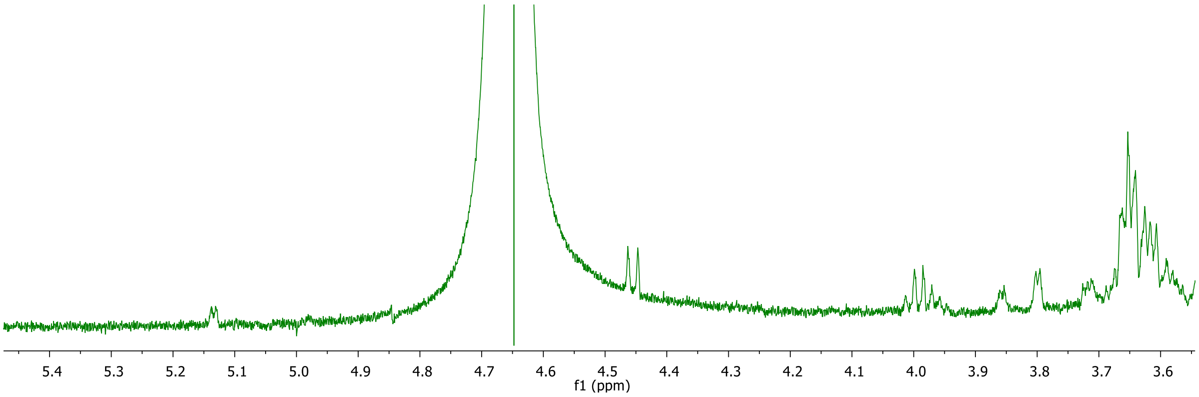

^{1}H NMR (500 MHz, D_2O) anomeric peaks: δ 4.46 ppm

C) ^{1}H NMR spectra of compound Lactose-QD **17b**

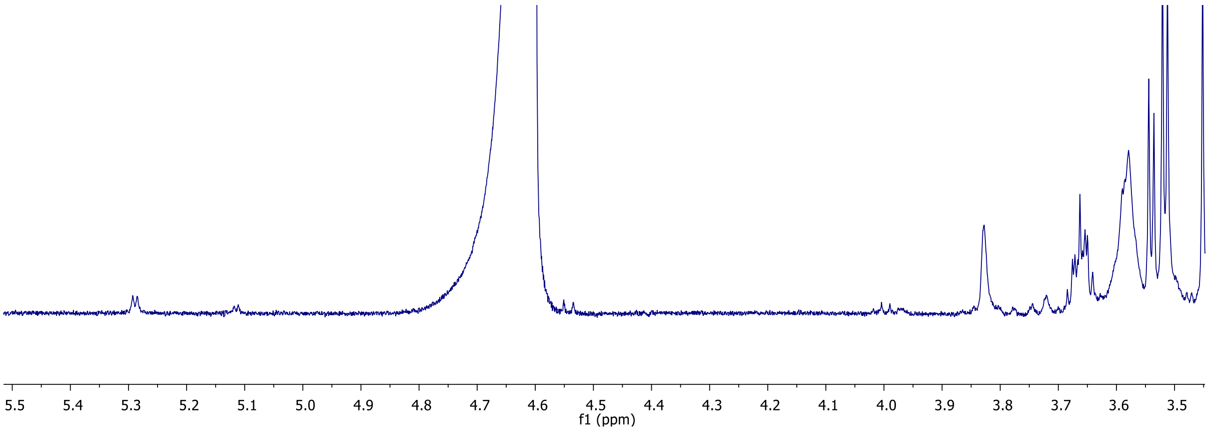

^{1}H NMR (500 MHz, D_2O) anomeric peaks: δ 4.58 ppm

D) ^{1}H NMR spectra of compound Lactose/maltotriose-QD **21b**

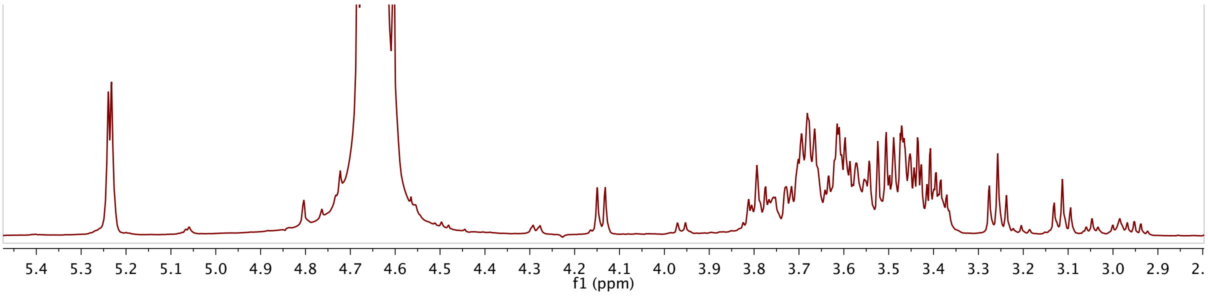

^{1}H NMR (500 MHz, D_2O) anomeric peaks: δ 5.24, 4.29, 4.14, 3.96 ppm

E) ^{1}H NMR spectra of compound Lactose/mannose-QD **20b**

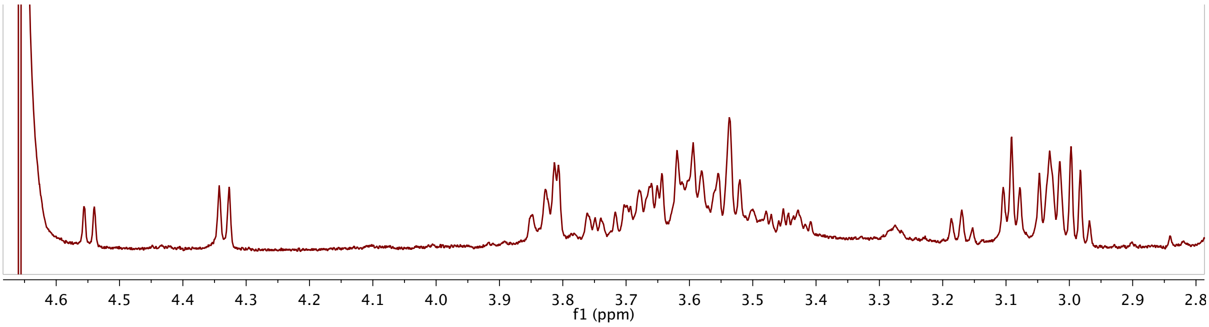

^{1}H NMR (500 MHz, D_2O) anomeric peaks: δ 4.55, 4.33 ppm

QD Characterization data

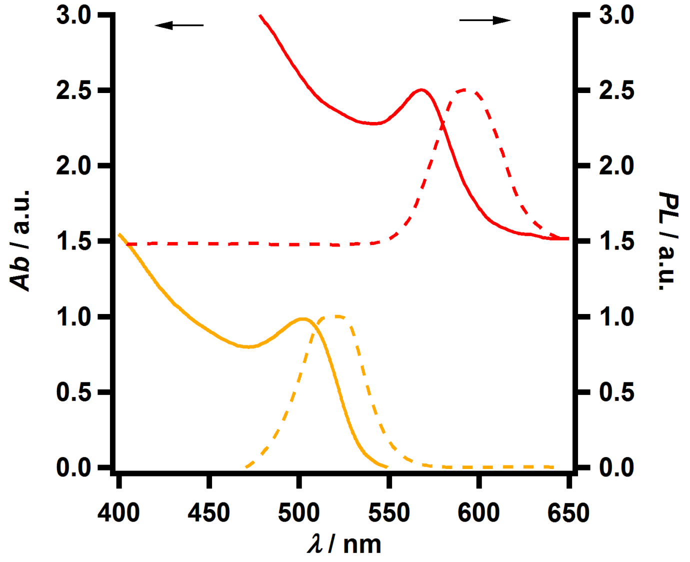

Figure S1. Room temperature absorption and luminescence spectra of the two different CdSe QDs used in the study (2.7±0.2nm and 4.0±0.4 nm).

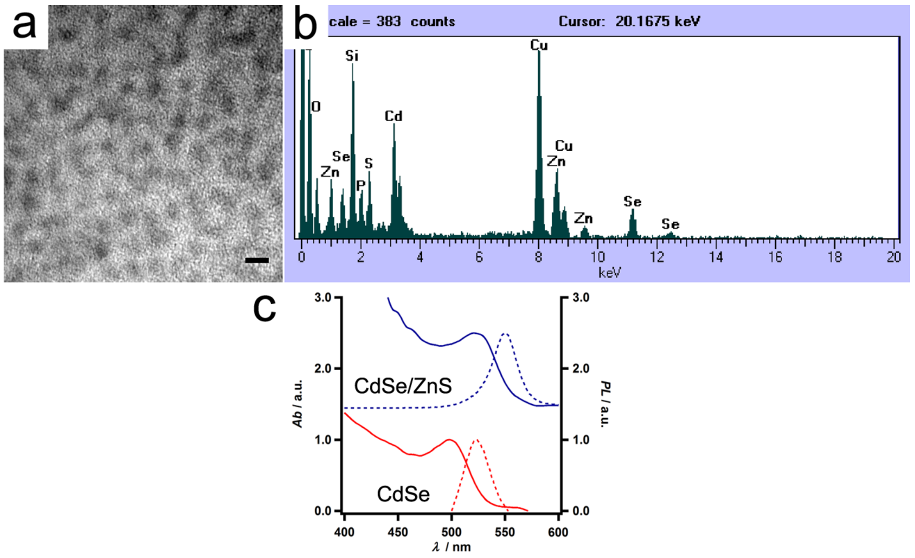

Figure S2. (a) Characteristic TEM of CdSe/ZnS (2.7±0.2nm). Scale bar = 5nm; (b) EDX analysis of CdSe/ZnS (2.7±0.2nm); (c) uv-vis (solid line) and Photoluminescence analysis (dotted line) of CdSe QDs before (red) and after ZnS coating (blue). The TEM analysis was performed on a JEOL 200 kV Hi Resolution TEM 2011 fitted with an EDX Oxford Instruments ISIS 300 system.

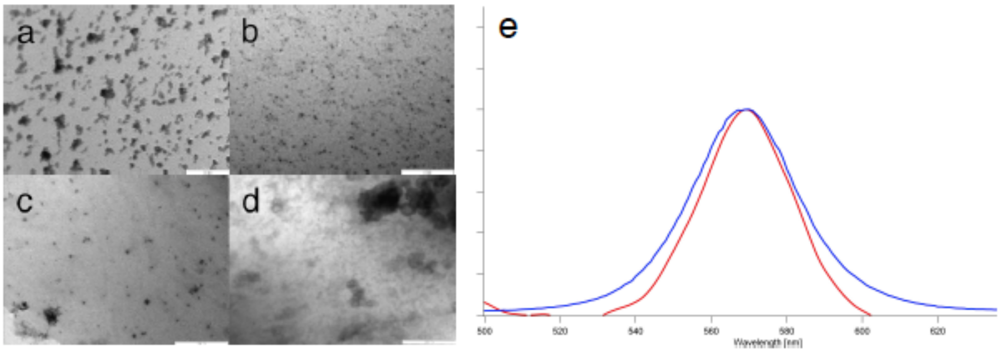

Figure S3. TEM images of a) PEG-QDs; b) 40% lactosamine-PEG-QDs **16c**; c) 60% lactosamine-PEG-QDs **17b**; d) 100% lactosamine-PEG-QDs **17a** (scale bar = 200 nm); e) Emission spectra of 60% Carboxy-PEG-QDs (blue line) **5b,** and 60% lactosamine-capped PEG-QDs (red line) **17b** in PBS. Note peak emission at 569 nm.

Cell culture

HeLa cells were maintained in Minimal Essential Medium (MEM) with GlutaMAX™ and 10% fetal bovine serum (FBS); AS cells were grown in Dulbecco's MEM with Glutamax and 10 % FBS. Both media were supplemented with antibiotic-antimycotic (Anti-Anti). Confluent cultures were detached from the surface using trypsin (Tryp LE Express) and plated at 10^4 cells/well in either petri dishes (Mat-Tek 35 mm, with 14mm glass microwell) for imaging, or 96-well plates for toxicity tests. Tests were carried out the next day as specified below. All cell culture media and additives were purchased from Invitrogen, Life Technologies.

Confocal microscopy

Cell trackers used were purchased from Life Technologies: CellLight® Reagents *BacMam 2.0: early Endosomes (Rab5a), late endosomes (Rab7a), ER-Tracker™ for the endoplasmic reticulum, BODIPY TR ceramide for the Golgi reticulum, mitochondria and LysoTracker™ for lysosomes, carbocyanine-based MitoTracker® (not dependent on oxidation status), and NucBlue™ Live Cell Stain (Hoechst 33342) for nuclei. These markers were used in line with manufacturer's protocols. All images were acquired on a Leica SP5 confocal system equipped with a Leica DMI 6000 inverted microscope. For the excitation of the QDs, either the blue laser or the blue lines of the argon ion laser (405 and 488nm respectively) were used. For the excitation of cellular manufacturers specifications were followed. The images were analysed using Volocity software (PerkinElmer). See full list of images at the end of ESI.

Toxicity assays

The influence of quantum dots on cell metabolism was assessed using AlamarBlue, a cytosolic substrate for reductive metabolism (resazurin to resorufin) whose fluorescence spectrum changes on reduction (Invitrogen Life Technologies). Cell numbers and proliferation were assessed by total protein, bicinchoninic acid assay (BCA, Pierce, ThermoScientific). Cells were exposed to quantum dots for 4, 24 and 48h in medium without foetal bovine serum. Results are expressed as changes in metabolism per µg protein. Figure S4 shows that toxic effects depend on oligosaccharide ligand concentration on QDs. The effects of a 24h exposure are illustrated in Figure S5a for Araki Sasaki cells. Figure S5b shows the effect of a 48h exposure on the metabolic compatibility of HeLa cells. After testing for homogeneity of variance, ANOVA or Kruskal-Wallis analysis of variance with post-hoc tests were used to assess the statistical significance of quantum dot effects.

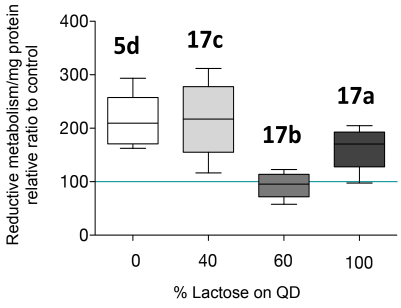

Figure S4 Effects of QDs on reductive metabolism is dependant on the concentration of ligand. Galactose (**14b**), glucose (**15b**), mannose (**16b**), lactose (**17b**), maltose (**18b**), maltotriose (**19b**), lactose/mannose (**20b**), lactose/maltotriose (**21b**) and dextran (**13**) functionalized QDs. Repeated measures ANOVA indicated that there were significant effects of glyco-QD dilution for every sugar density $p < 0.0001$. Differences between sugar densities were significant at $p < 0.0001$.

HeLa cells were exposed to Lactose-functionalised QDs for 24h. Foetal bovine serum was omitted from the medium to avoid masking of toxic effects on metabolism.

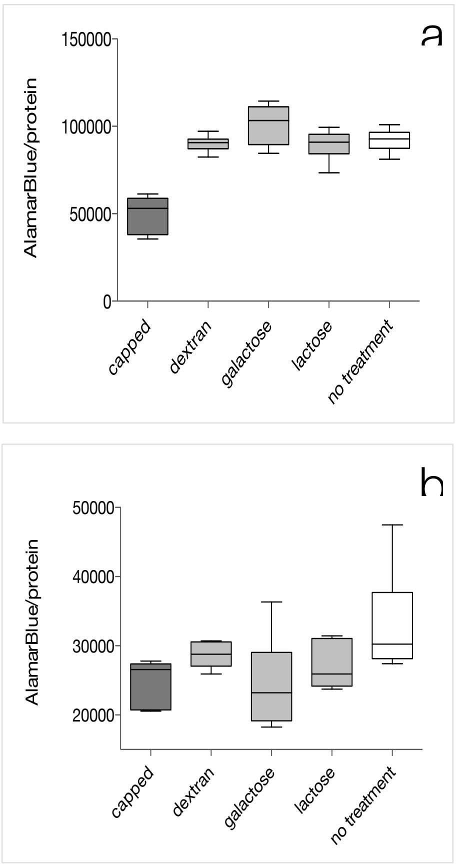

Figure S5 Reductive metabolism relatie to cell numbers after exposure to equal doses of differently functionalized Quantum dots.

a. AS cells exposed for 24 hours; cells exposed to dextran-, galactose- and lactose-functionalised QDs are not significantly different from the controls, while cells exposed to capped quantum dots have significantly lower metabolic activity than the controls ($p<0.001$).

b. HeLa cells exposed for 28 hours; dextran- and lactose-QDs have no effect on HeLa cell metabolism. Capped QDs and galactose-QDs are more toxic than no treatment ($p<0.05$).

c. Colocalization Analysis

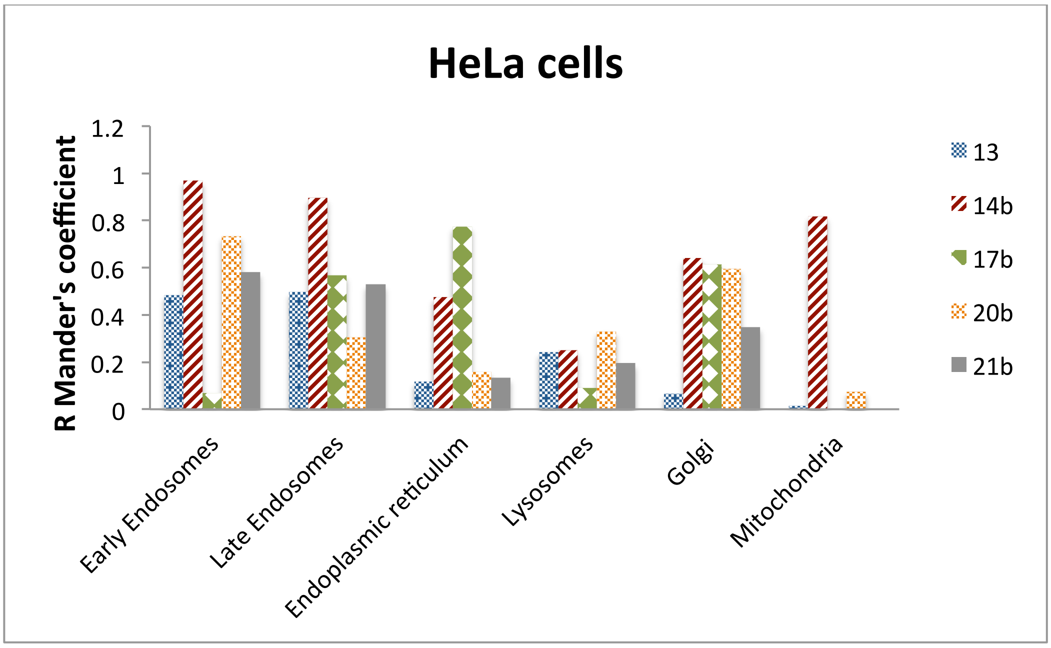

d.

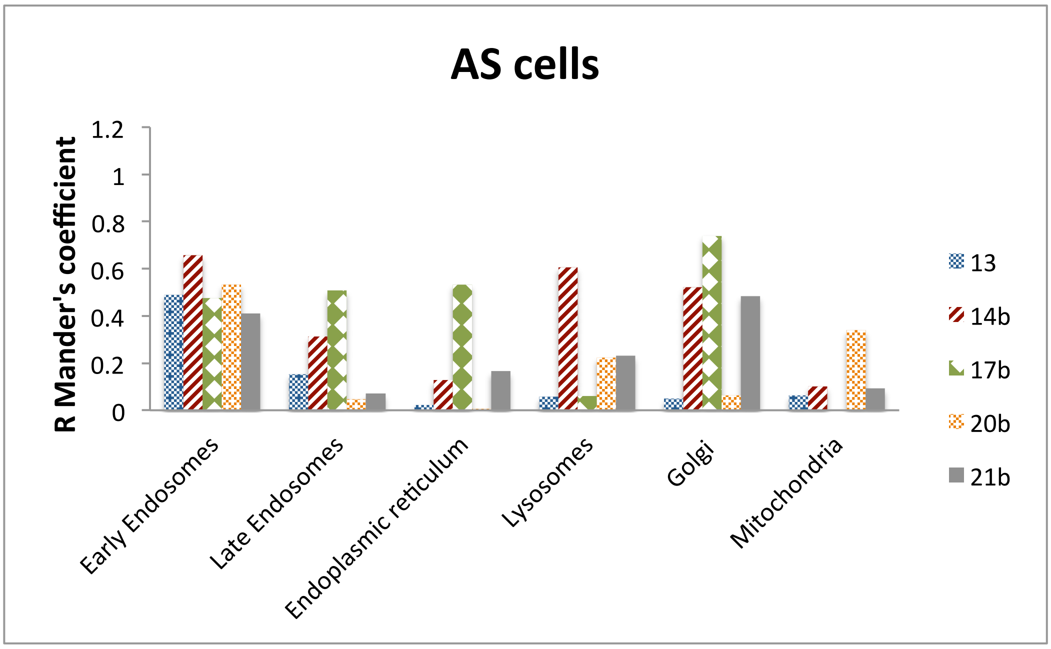

Figure S6. Manders' overlap coefficient (*R*)[6,7] for Dextran QD **13** , Galactose QD **14b**, Lactose **17b**, Lactose/Mannose QD **20b** and Lactose/Maltotriose QD **21b**: Manders' (*R*) coefficients for internalization of Galactose, Maltotriose, Lactose and Dextran in A) HeLa cells and B) AS cells. Note that most galactose-QDs were located in the early and Late endosomes in HeLa, while just over half colocalised with early endosomes in AS. The low level of colocalization of other sugar-QDs with intracellular organelles is supported by their presence in the cytosol. Images and regions of interest were analyzed for each experimental condition. The data were analyzed by Student's *t* test or one-way analysis of variance; $P < 0.05$ was considered significant.

CLEM Experimental Protocol

The Correlative Light Electron Microscopy (CLEM) approach is described in detail in van Weering et al., 2010.[8] In brief, cells were grown on special gridded glass bottom dishes. QDs were added to the cells for 2h and analysed under a Leica SP5 Confocal microscope registering both the fluorescence from the QDs as well as the bright field image with the finder pattern and outline of the cells. After fixation and processing for Electron Microscopy, ultrathin sections of the cells were made. Some sections were cut at 70 nm and stained for conventional TEM and Dark Field STEM imaging, and some specimens were cut at 300 nm, left unstained and imaged by TEM, STEM and Electron Tomography. The electron microscopy was done on a FEI Tecnai 20 TEM at 200kV fitted with a Fischione High Angle Anular Dark Field (HAADF) detector. This latter technique is extremely sensitive to atomic number contrast so that the QDs show up as particularly bright spots. The electron tomography has been used to confirm that the QDs are truly inside the cell and to further investigate the nature of some of the cellular structures enclosing them. This data is not presented here. The images were TEM images were recorded on an FEI 4k x 4k Eagle CCD camera.

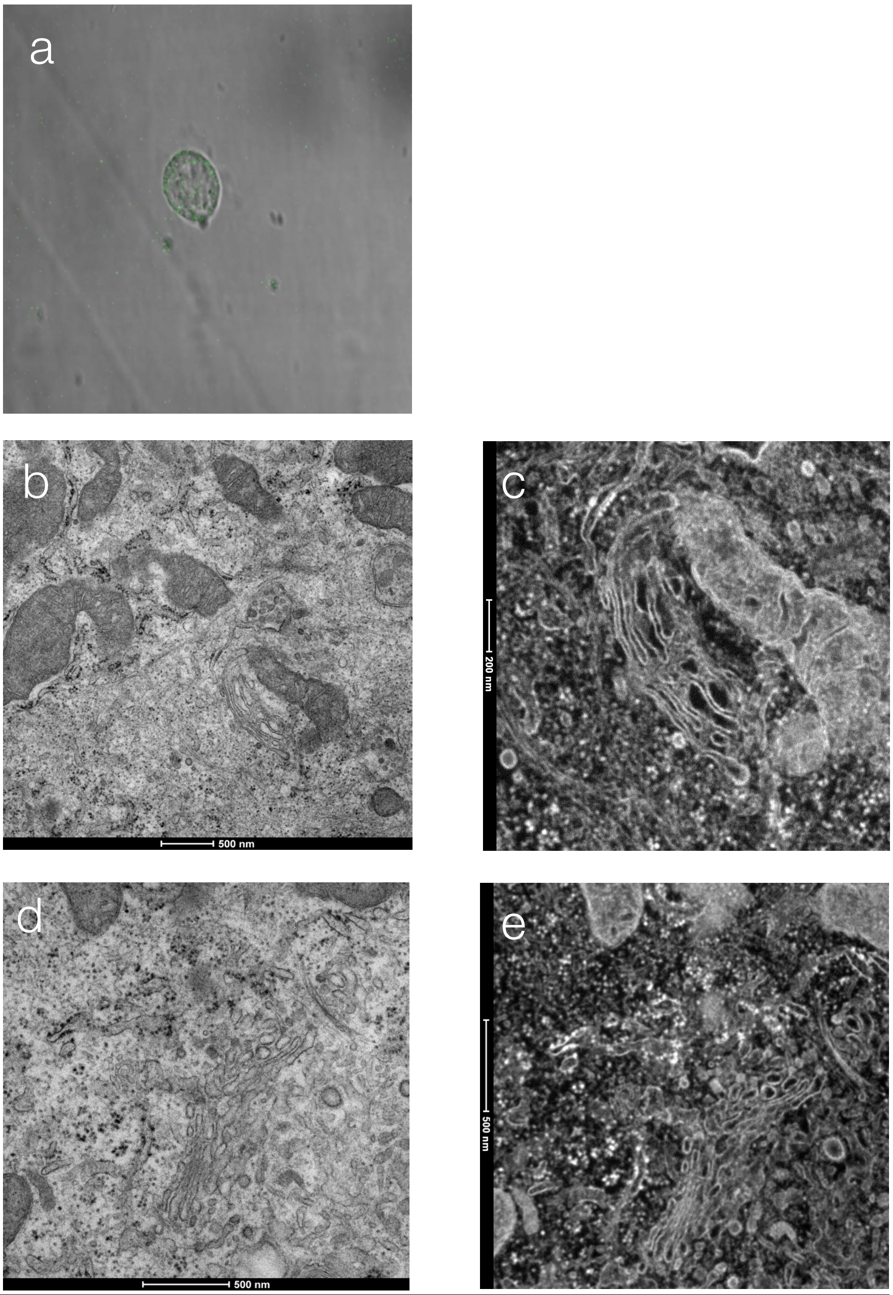

Figure S7. Correlative microscopy of AS cell with Lactose (**17b**) Qds

a. Confocal image of the cell with most Lactose-QDs (Particle size ~ 15nm) bound to ER membranes
b. General view
c. STEM of QDs in Golgi vesicles
d. ER and Golgi: QDs on ER not in Golgi (BF)
e. STEM image of d.

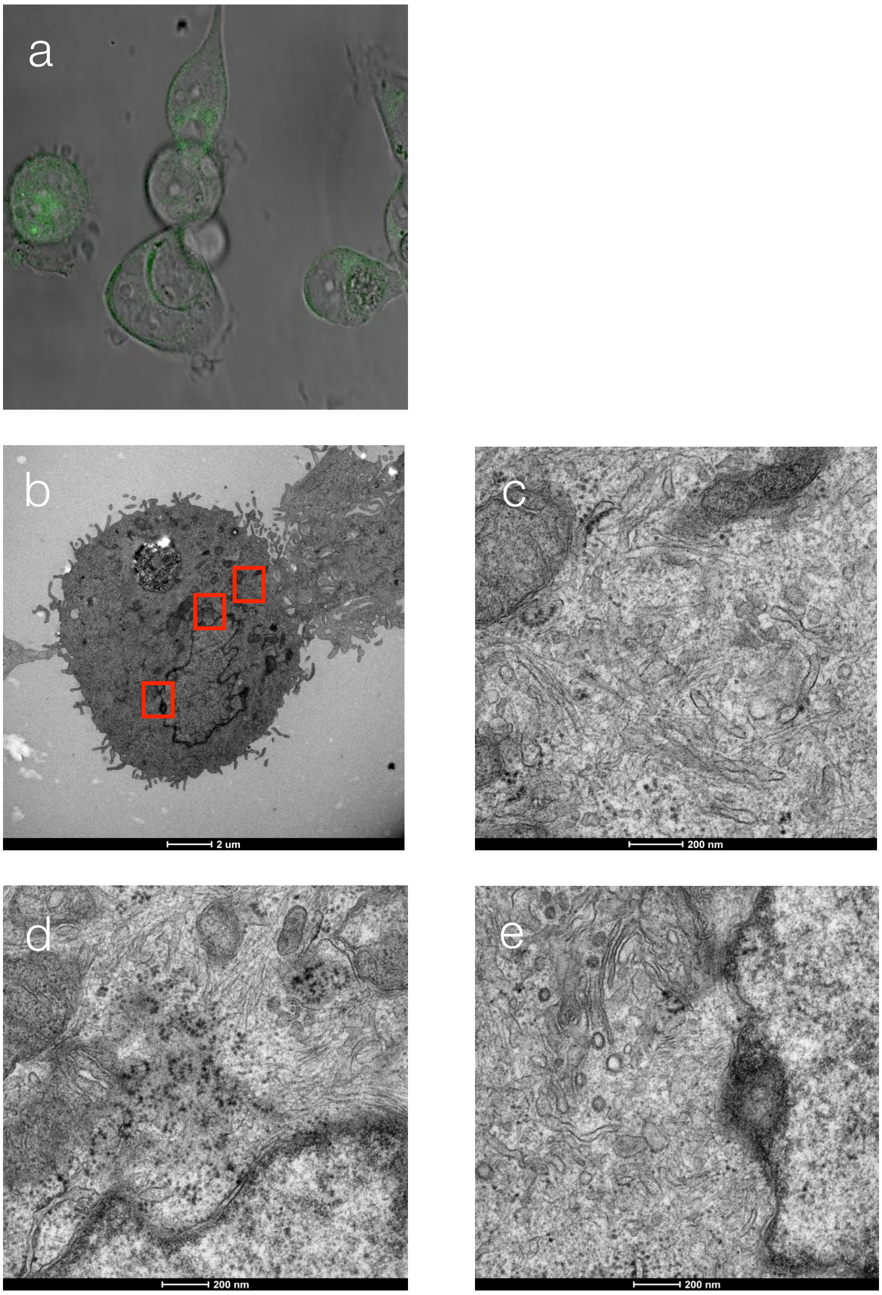

Figure S8. Correlative microscopy of AS cells with Lactose-Maltotriose (**21b**) bifunctional QDs

a. Confocal image. Note the large vesicles, less ER localization than with Lactose (**17b**) QD's, and worm-like aggregates in the cytoplasm
b. B. General view of a cell (HAADF). The three inserts are magnified, top right to bottom left in the next panels.
c. QDs in Golgi (BF)
d. QDs in the cytosol (BF)
e. QDs in Golgi

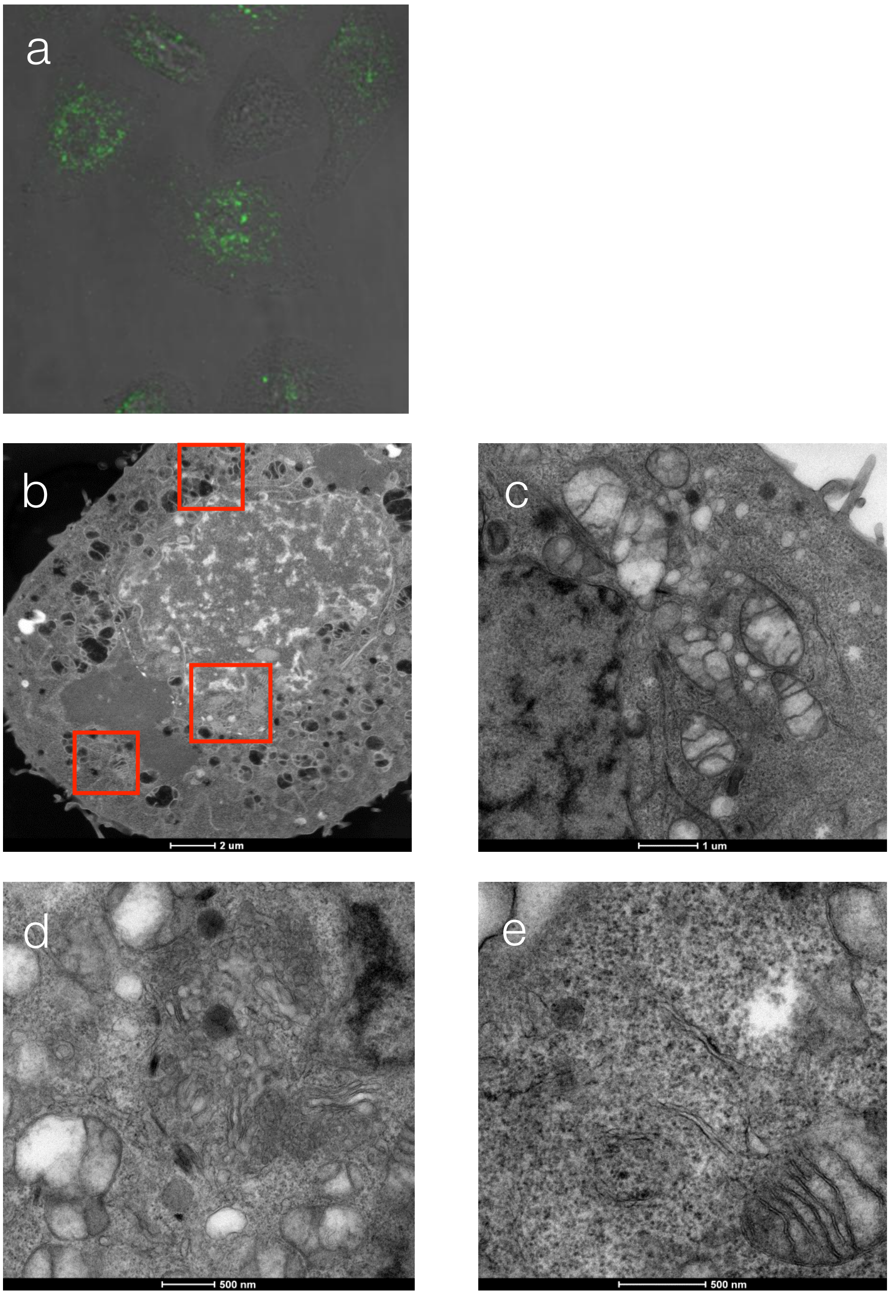

Figure S9. Correlative microscopy of HeLa cells with Lactose (**17b**) QDs

a. confocal image QDs are more localized in specific areas than in AS. Note the perinuclear and ER localization
b. general view (HAADF); inserts are magnified top to bottom in the following panels
c. QDs in ER (BF)
d. QDs in Golgi (BF)
e. QDs in ER (BF)

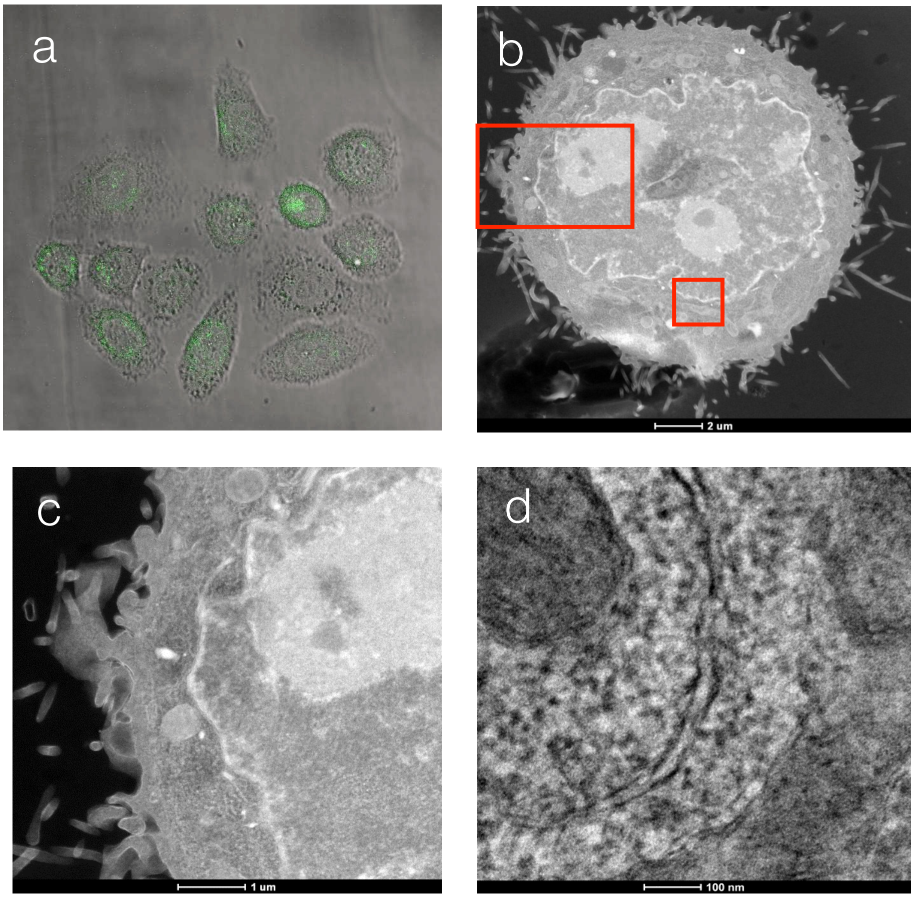

Figure S10 Correlative micrscopy of HeLa cells with bifunctional Lactose-Maltotriose QDs

a. confocal image
b. general view (HAADF)
c. perinuclear QDs
d. QDs on ER

References:

(1) Powers, S. P.; Foo, I.; Pinon, D.; Klueppelberg, U. G.; Hedstrom, J. F.; Miller, L. J. *Biochemistry* **1991**, *30*, 676.

(2) Liu, W.; Howarth, M.; Greytak, A. B.; Zheng, Y.; Nocera, D. G.; Ting, A. Y.; Bawendi, M. G. *J Am Chem Soc* **2008**, *130*, 1274.

(3) Wakao, M.; Sumida, Y. In http://www.sumobrain.com/patents/JP2011209282.html CORP, K. U. S.-B., Ed. Japan, 2011; Vol. JP2011209282.

(4) Bejugam, M.; Flitsch, S. L. *Org Lett* **2004**, *6*, 4001.

(5) Likhosherstov, L. M.; Novikova, O. S.; Derevitskaja, V. A.; Kochetkov, N. K. *Carbohydr Res* **1986**, *146*, C1.

(6) Manders, E. M. M.; Verbeek, F. J.; Aten, J. A. *J Microsc-Oxford* **1993**, *169*, 375.

(7) Zinchuk, V.; Zinchuk, O. *Curr Protoc Cell Biol* **2008**, *Chapter 4*, Unit 4 19.

(8) van Weering, J. R. T.; Brown, E.; Sharp, T. H.; Mantell, J.; Cullen, P. J.; Verkade, P. Method Cell Biol. **2010**, 96, 619.

Confocal Images

Colocalization Experiments HeLa

Early Endosomes

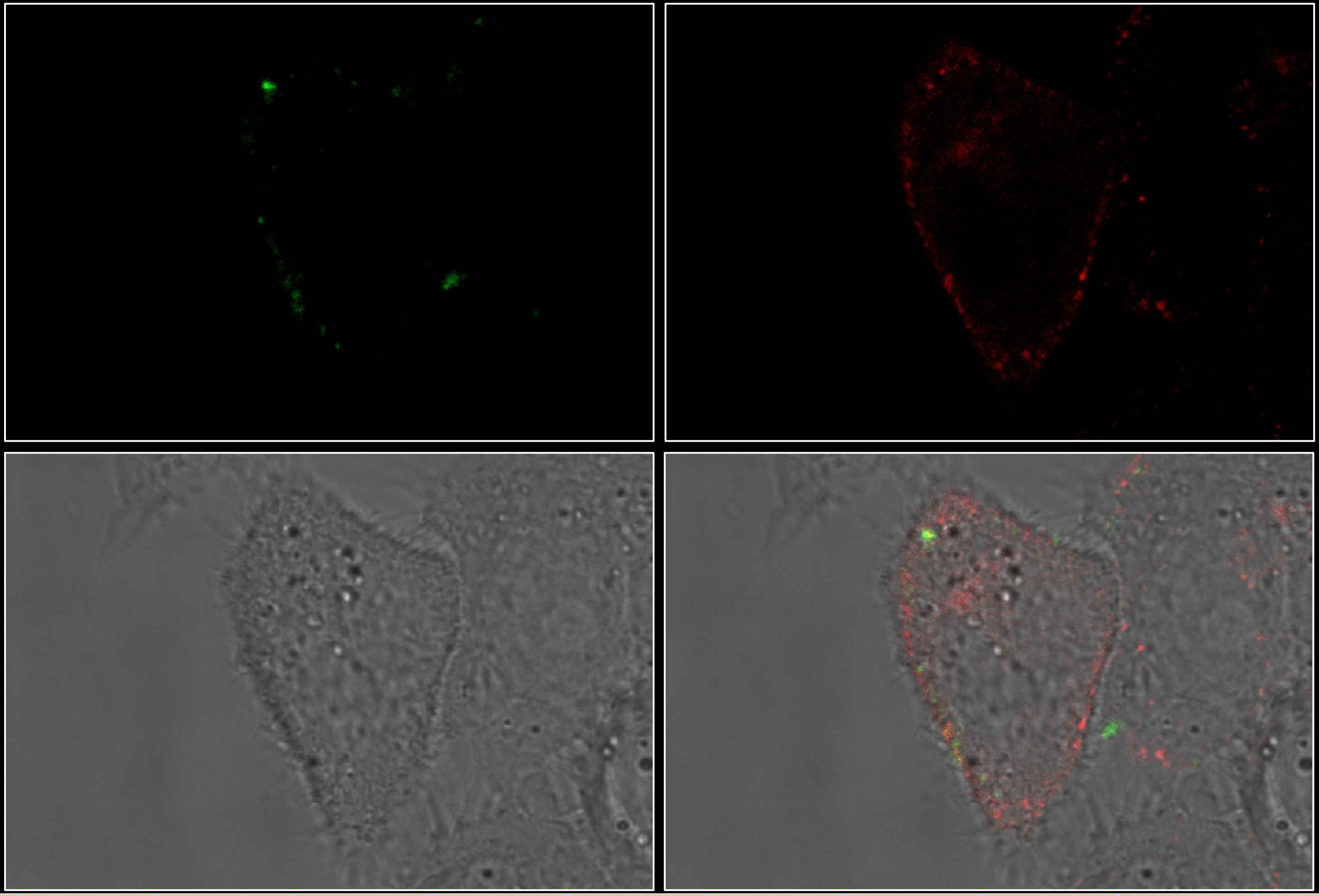

Pearsons Correlation	Colocalization Coefficient Mx	Colocalization Coefficient My	Overlap Coefficient R	Overlap Coefficient Kx	Overlap Coefficient Ky	X Min Threshold	X Max Threshold	Y Min Threshold	Y Max Threshold	Voxel Ratio Ch.X/Ch.Y	Global Pearsons Correlation
0.195	1	1	0.474	1.633	0.139	1	255	1	255	1	0.195

Colocalization Experiments HeLa

Dextran-QD (13)

Late Endosomes

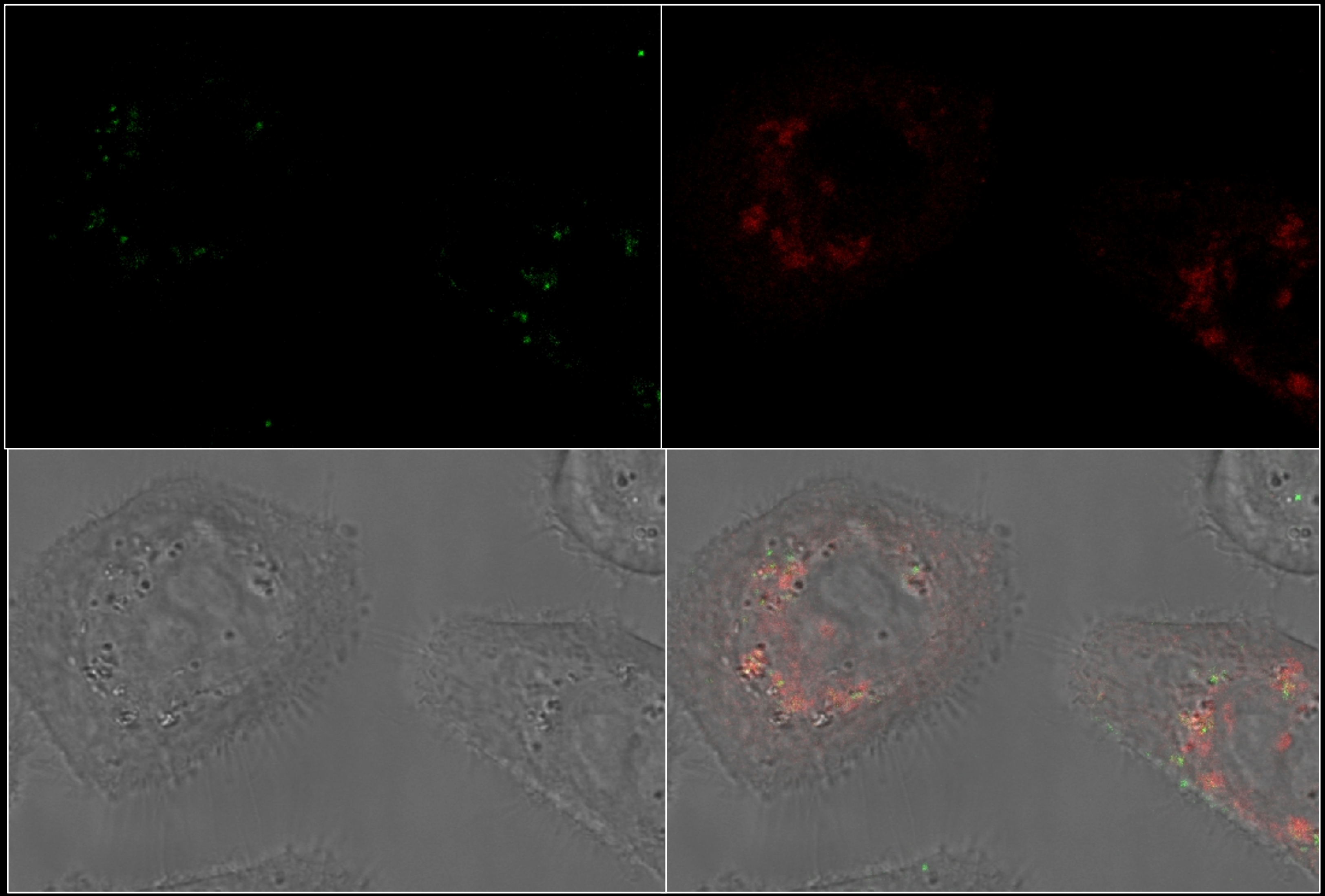

Pearsons Correlation	Colocalization Coefficient Mx	Colocalization Coefficient My	Overlap Coefficient R	Overlap Coefficient Kx	Overlap Coefficient Ky	X Min Threshold	X Max Threshold	Y Min Threshold	Y Max Threshold	Voxel Ratio Ch.X/Ch.Y	Global Pearsons Correlation
0.299	1	1	0.533	1.58	0.185	1	201	1	201	1	0.299

Colocalization Experiments HeLa

Dextran-QD (13)

Lysosomes

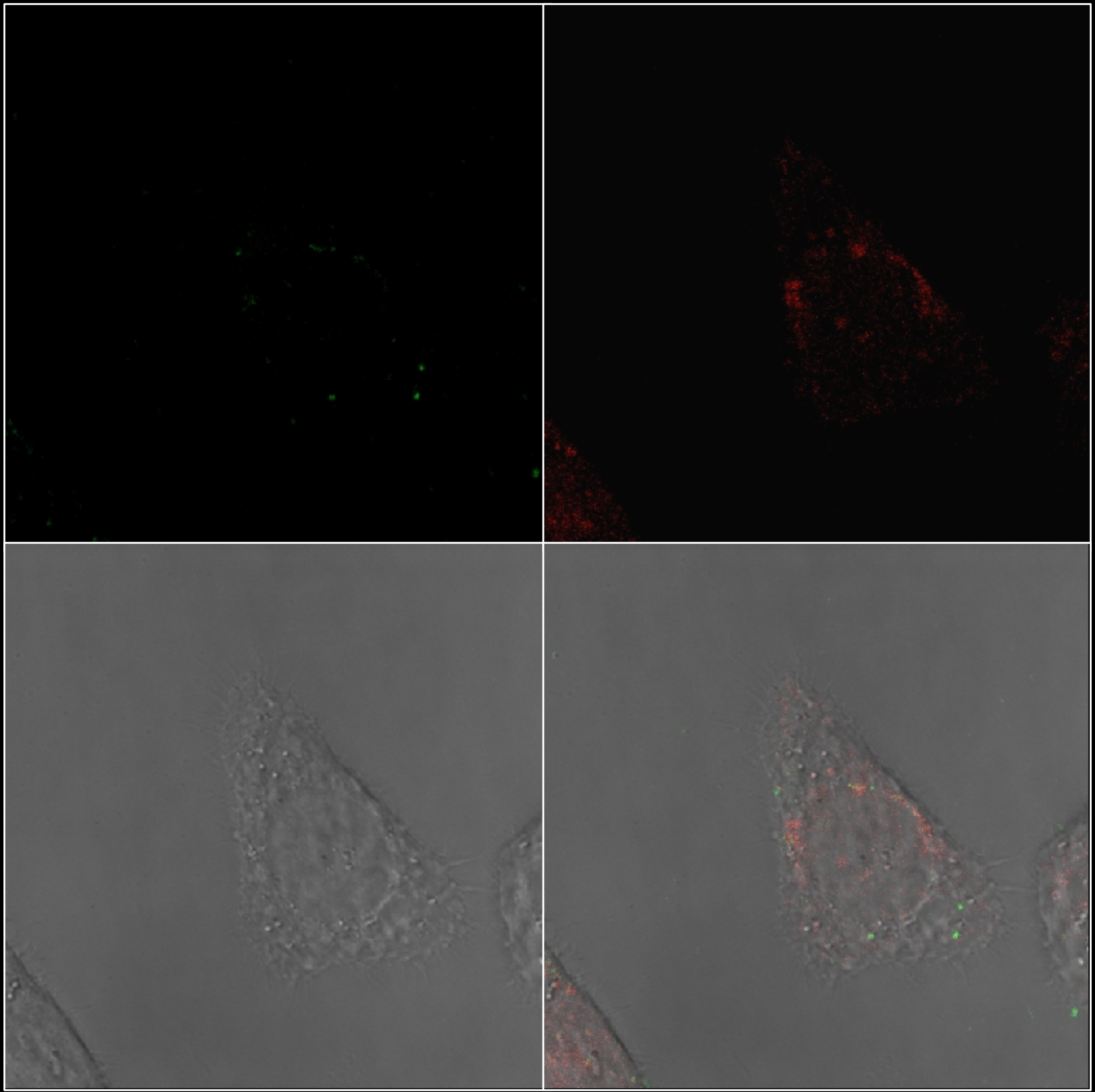

Pearsons Correlation	Colocalization Coefficient Mx	Colocalization Coefficient My	Overlap Coefficient R	Overlap Coefficient Kx	Overlap Coefficient Ky	X Min Threshold	X Max Threshold	Y Min Threshold	Y Max Threshold	Voxel Ratio Ch.X/Ch.Y	Global Pearsons Correlation
0.116	0.391	0.225	0.227	0.596	0.087	5	170	18	170	0.6	0.137

Colocalization Experiments HeLa

Dextran-QD (13)

ER

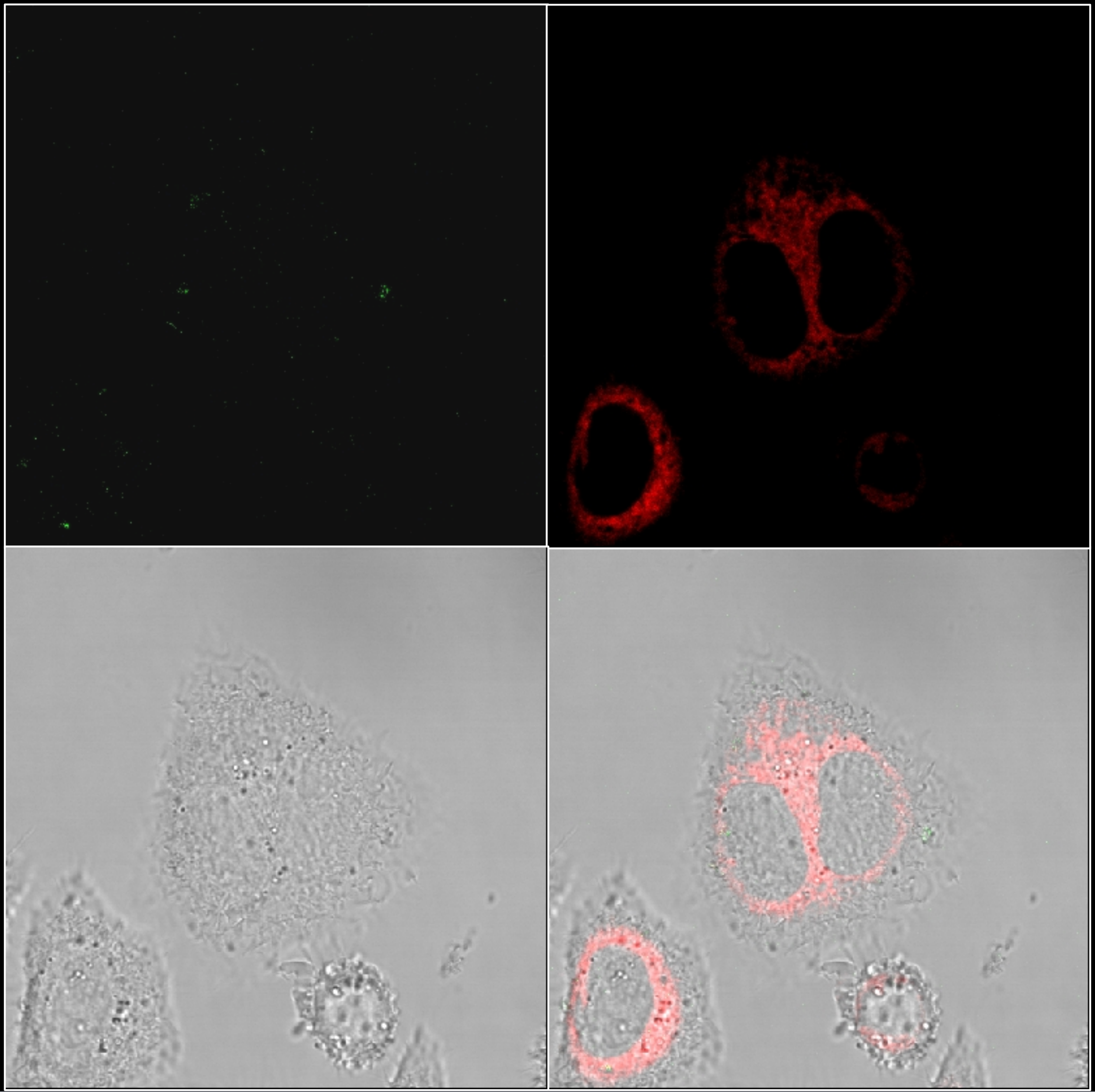

Pearsons Correlation	Colocalization Coefficient Mx	Colocalization Coefficient My	Overlap Coefficient R	Overlap Coefficient Kx	Overlap Coefficient Ky	X Min Threshold	X Max Threshold	Y Min Threshold	Y Max Threshold	Voxel Ratio Ch.X/Ch.Y	Global Pearsons Correlation
-0.064	0.382	0.056	0.122	1.235	0.012	12	255	41	255	0.144	0.063

Colocalization Experiments HeLa

Dextran-QD (13)

Golgi

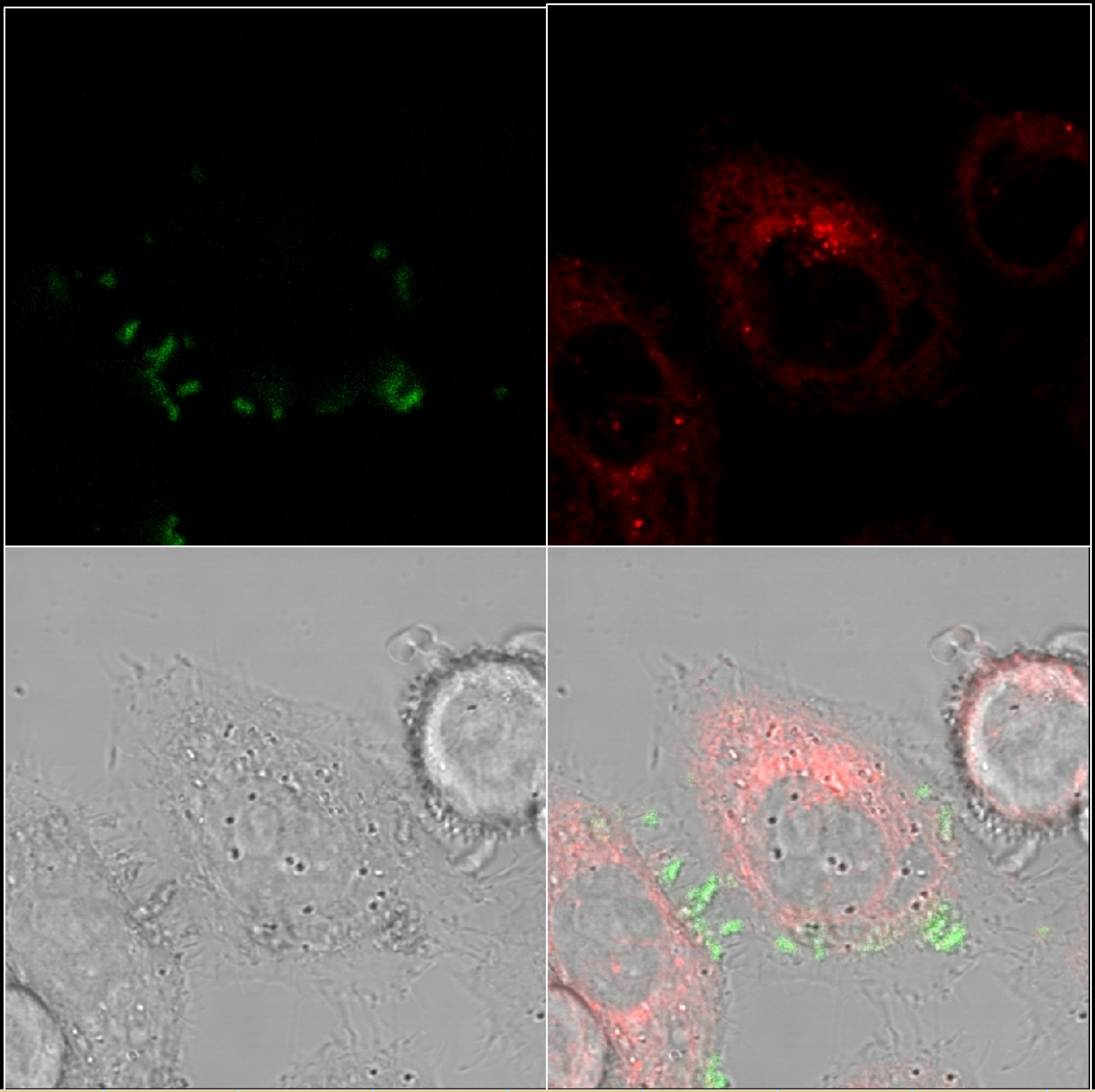

Pearsons Correlation	Colocalization Coefficient Mx	Colocalization Coefficient My	Overlap Coefficient R	Overlap Coefficient Kx	Overlap Coefficient Ky	X Min Threshold	X Max Threshold	Y Min Threshold	Y Max Threshold	Voxel Ratio Ch.X/Ch.Y	Global Pearsons Correlation
-0.147	0.121	0.123	0.08	0.139	0.058	11	255	27	255	0.857	-0.04

Colocalization Experiments HeLa

Dextran-QD (13)

Mitochondria

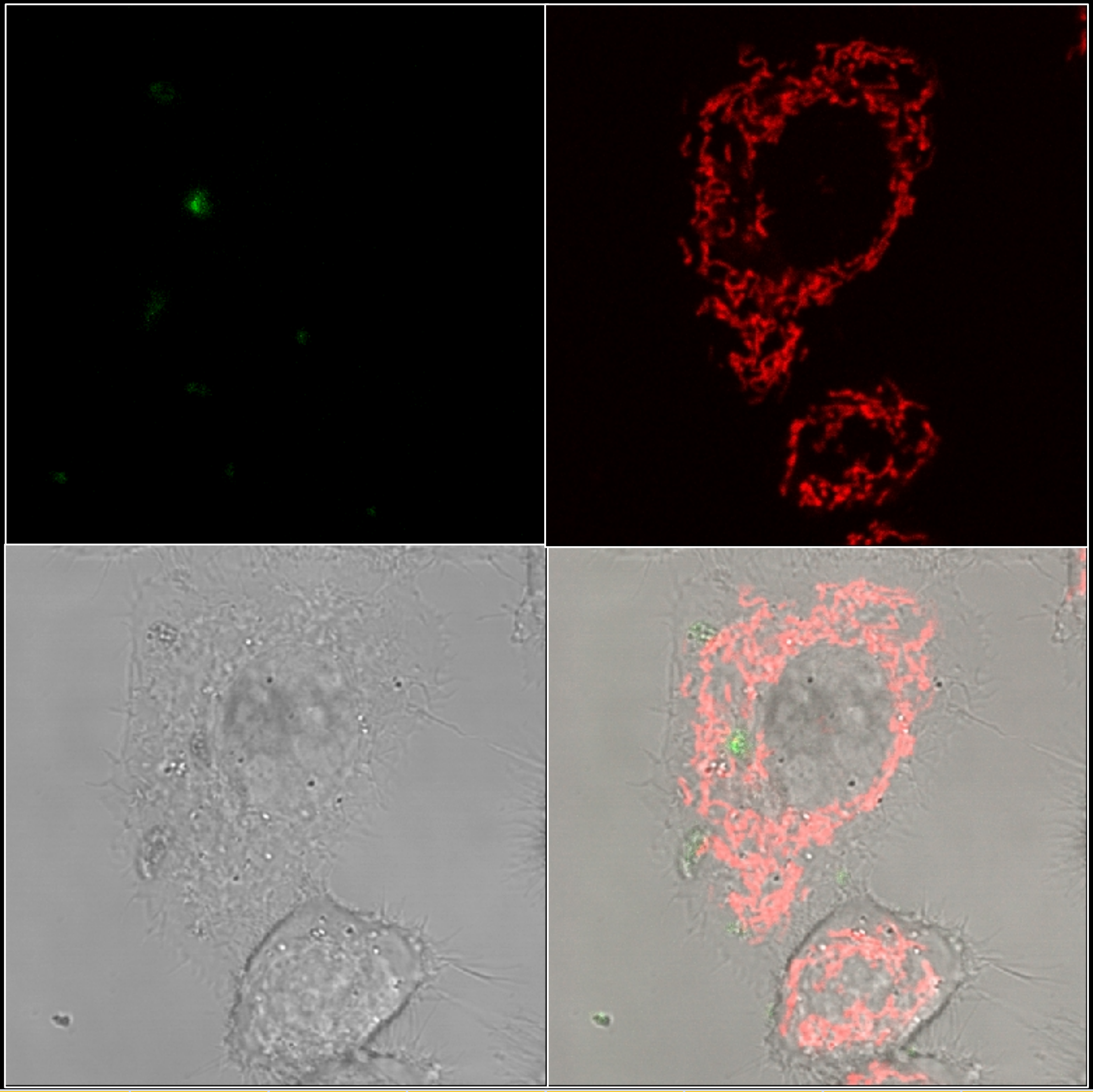

Pearsons Correlation	Colocalization Coefficient Mx	Colocalization Coefficient My	Overlap Coefficient R	Overlap Coefficient Kx	Overlap Coefficient Ky	X Min Threshold	X Max Threshold	Y Min Threshold	Y Max Threshold	Voxel Ratio Ch.X/Ch.Y	Global Pearsons Correlation
-0.152	0.046	0.029	0.023	0.16	0.005	12	255	70	255	0.517	-0.055

Colocalization Experiments AS

Dextran-QD (13)

Early Endosomes

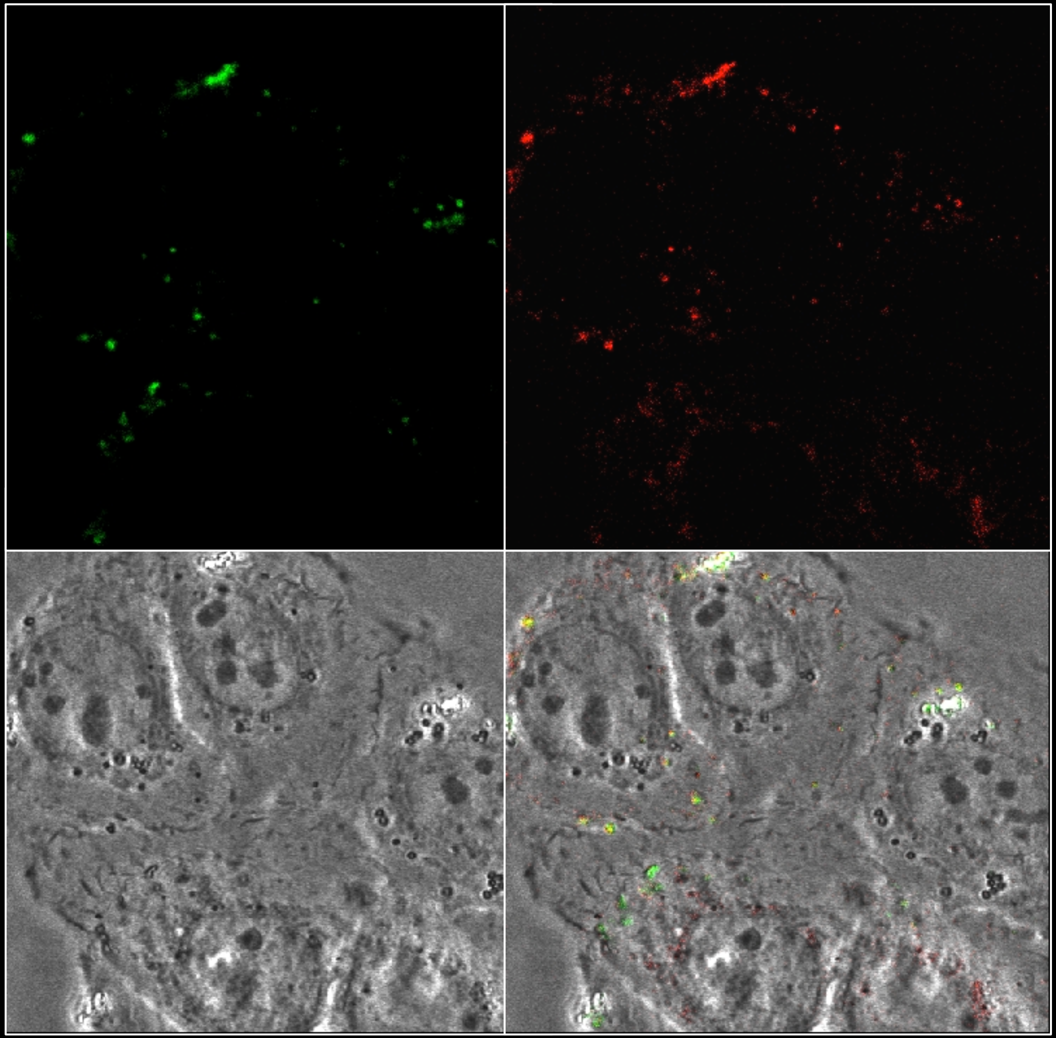

Pearsons Correlation	Colocalization Coefficient Mx	Colocalization Coefficient My	Overlap Coefficient R	Overlap Coefficient Kx	Overlap Coefficient Ky	X Min Threshold	X Max Threshold	Y Min Threshold	Y Max Threshold	Voxel Ratio Ch.X/Ch.Y	Global Pearsons Correlation
0.4365	0.479	0.824	0.577	0.438	0.773	6	255	5	255	2.83	0.504

Colocalization Experiments AS

Dextran-QD (13)

Late Endosomes

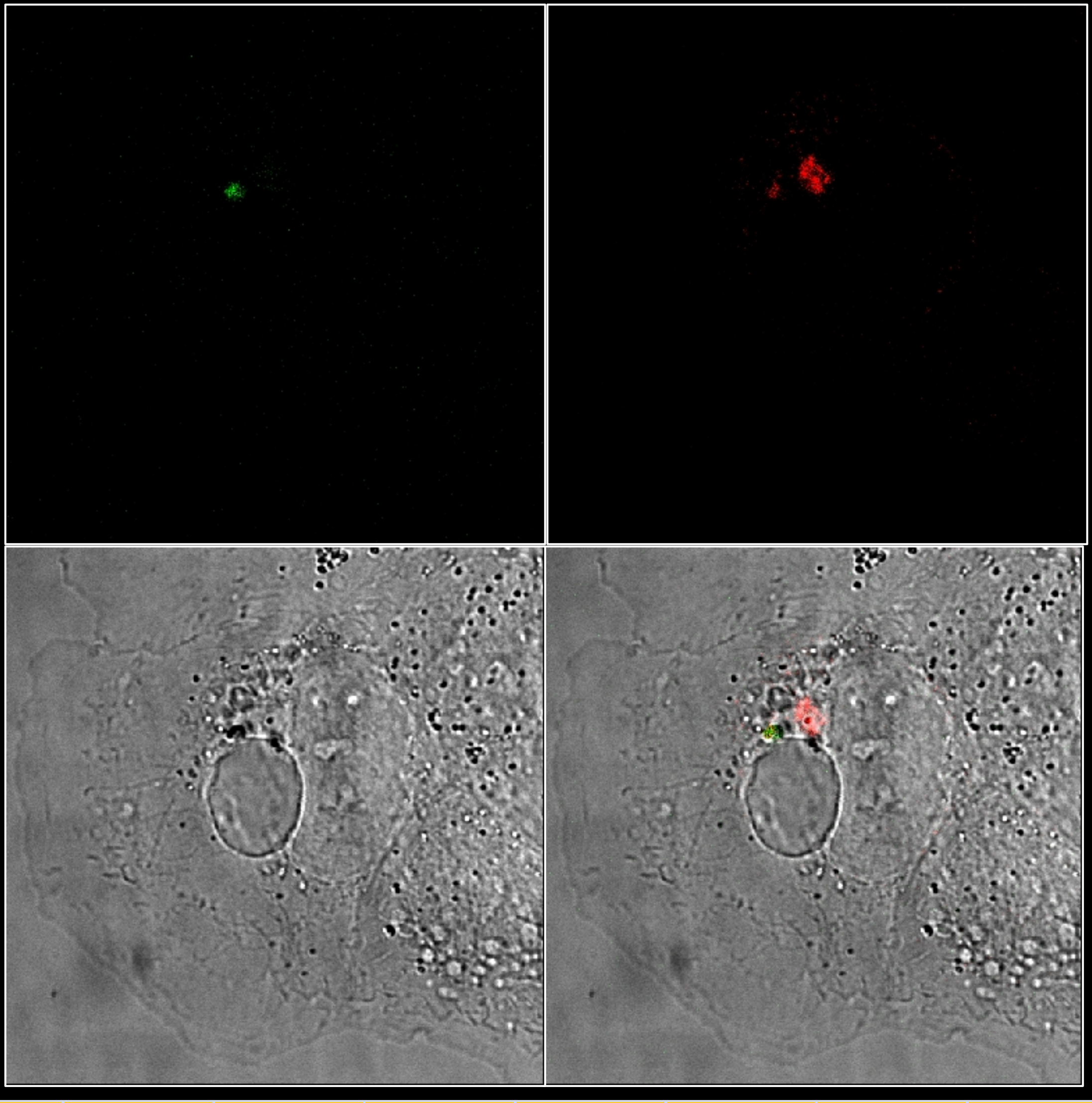

Pearsons Correlation	Colocalization Coefficient Mx	Colocalization Coefficient My	Overlap Coefficient R	Overlap Coefficient Kx	Overlap Coefficient Ky	X Min Threshold	X Max Threshold	Y Min Threshold	Y Max Threshold	Voxel Ratio Ch.X/Ch.Y	Global Pearsons Correlation
0.1525	1	1	0.197	0.7165	0.0542	0	255	0	255	1	0.1525

Colocalization Experiments AS

Dextran-QD (13)

Late Endosomes

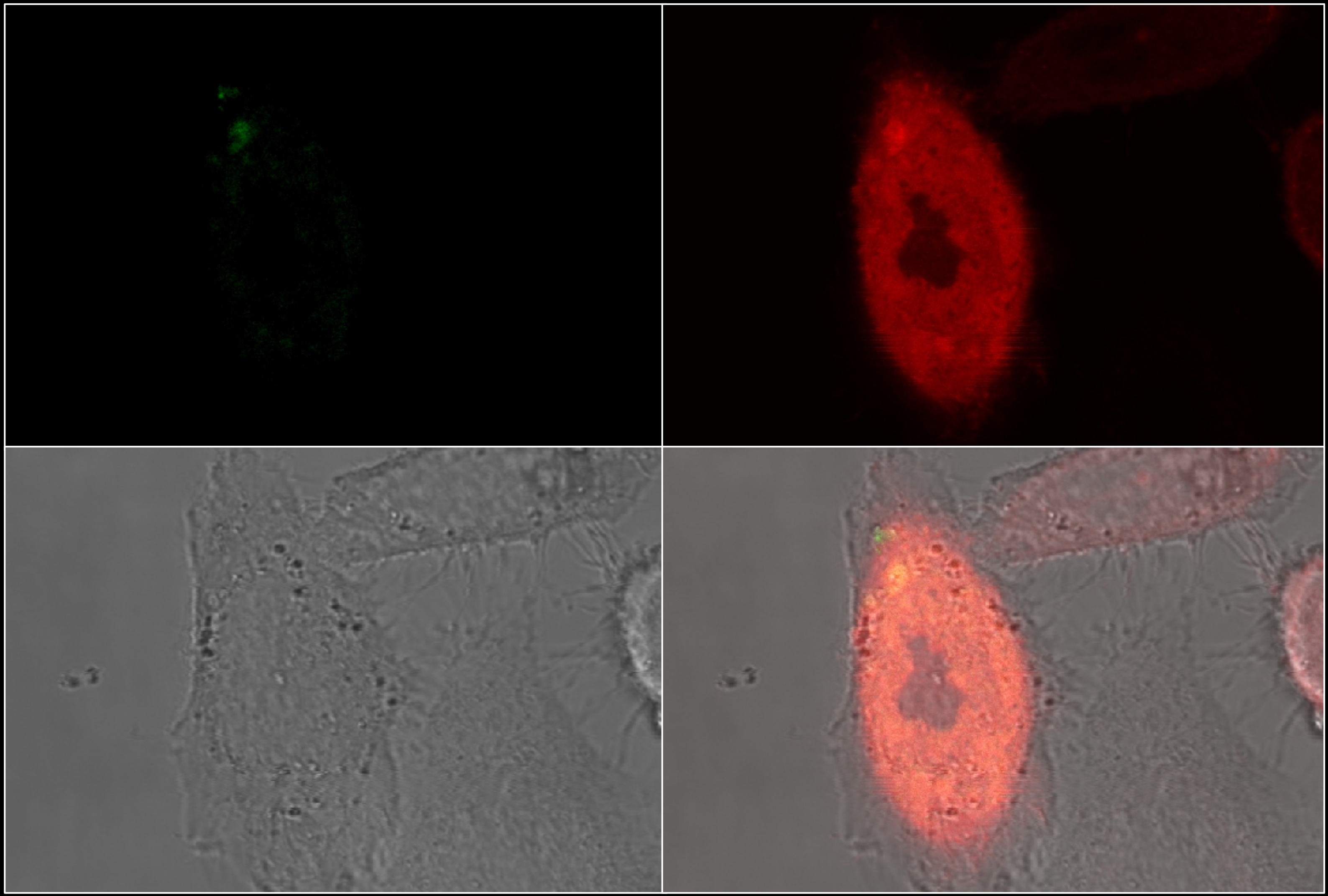

Colocalization Experiments AS

Dextran-QD (13)

Lysosomes

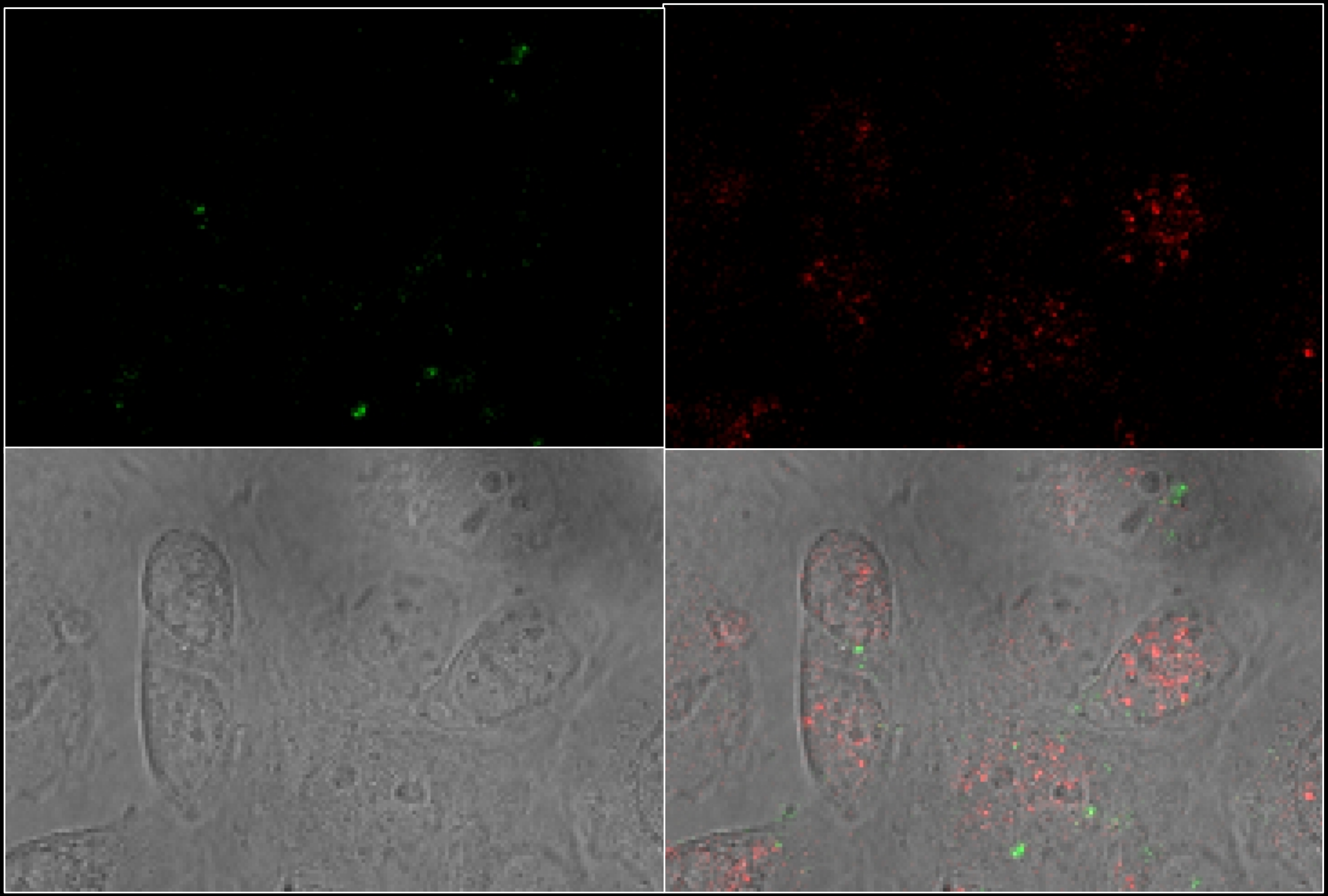

Pearsons Correlation	Colocalization Coefficient Mx	Colocalization Coefficient My	Overlap Coefficient R	Overlap Coefficient Kx	Overlap Coefficient Ky	X Min Threshold	X Max Threshold	Y Min Threshold	Y Max Threshold	Voxel Ratio Ch.X/Ch.Y	Global Pearsons Correlation
-0.044	0.031	0.234	0.06	0.025	0.149	29	255	22	255	6.865	0.018

S31

Colocalization Experiments AS

Dextran-QD (13)

ER

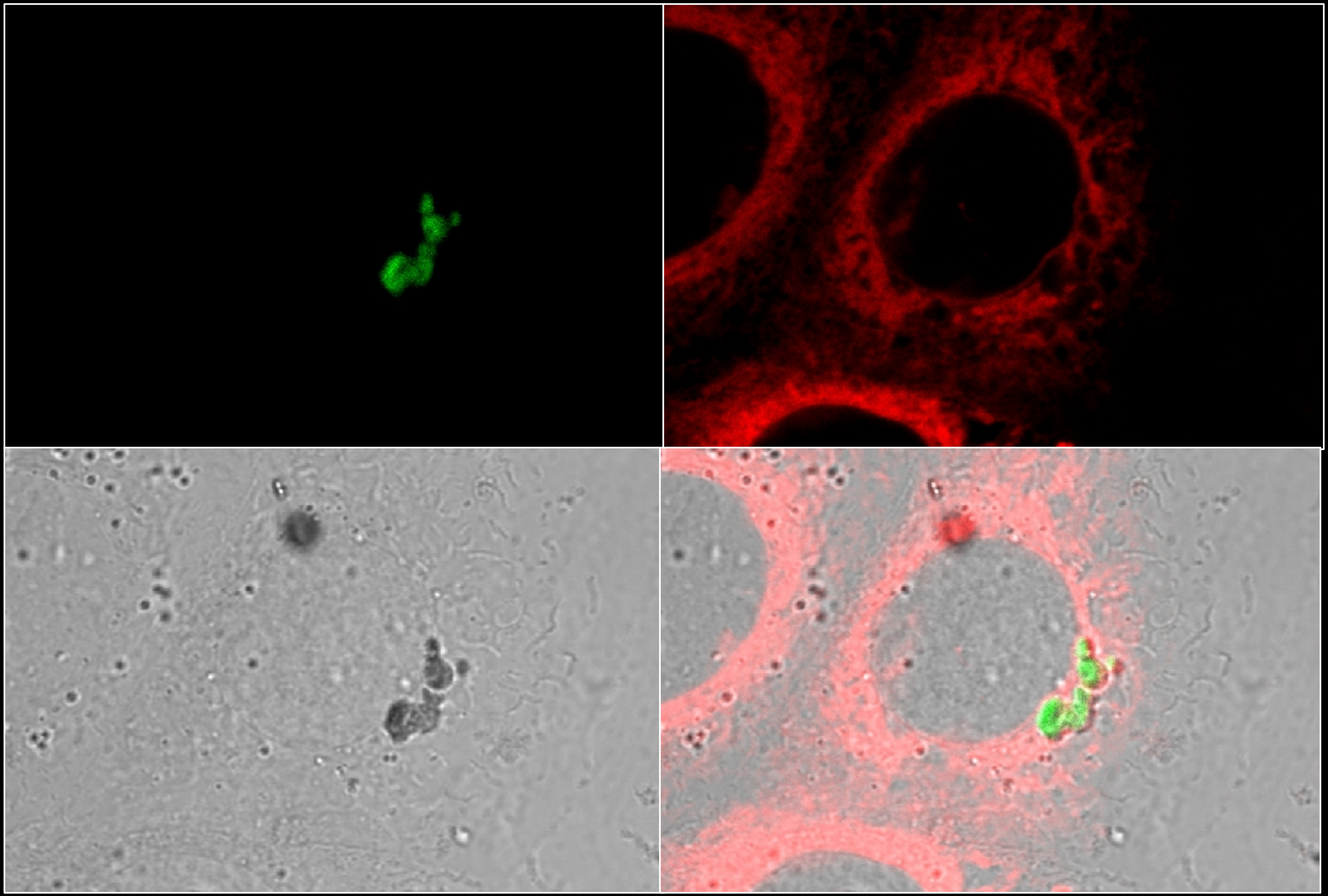

Pearsons Correlation	Colocalization Coefficient Mx	Colocalization Coefficient My	Overlap Coefficient R	Overlap Coefficient Kx	Overlap Coefficient Ky	X Min Threshold	X Max Threshold	Y Min Threshold	Y Max Threshold	Voxel Ratio Ch.X/Ch.Y	Global Pearsons Correlation
-0.272	0.116	0.213	0.081	0.151	0.073	18	255	96	255	1.263	-0.068

Colocalization Experiments AS

Dextran-QD (13)

Golgi

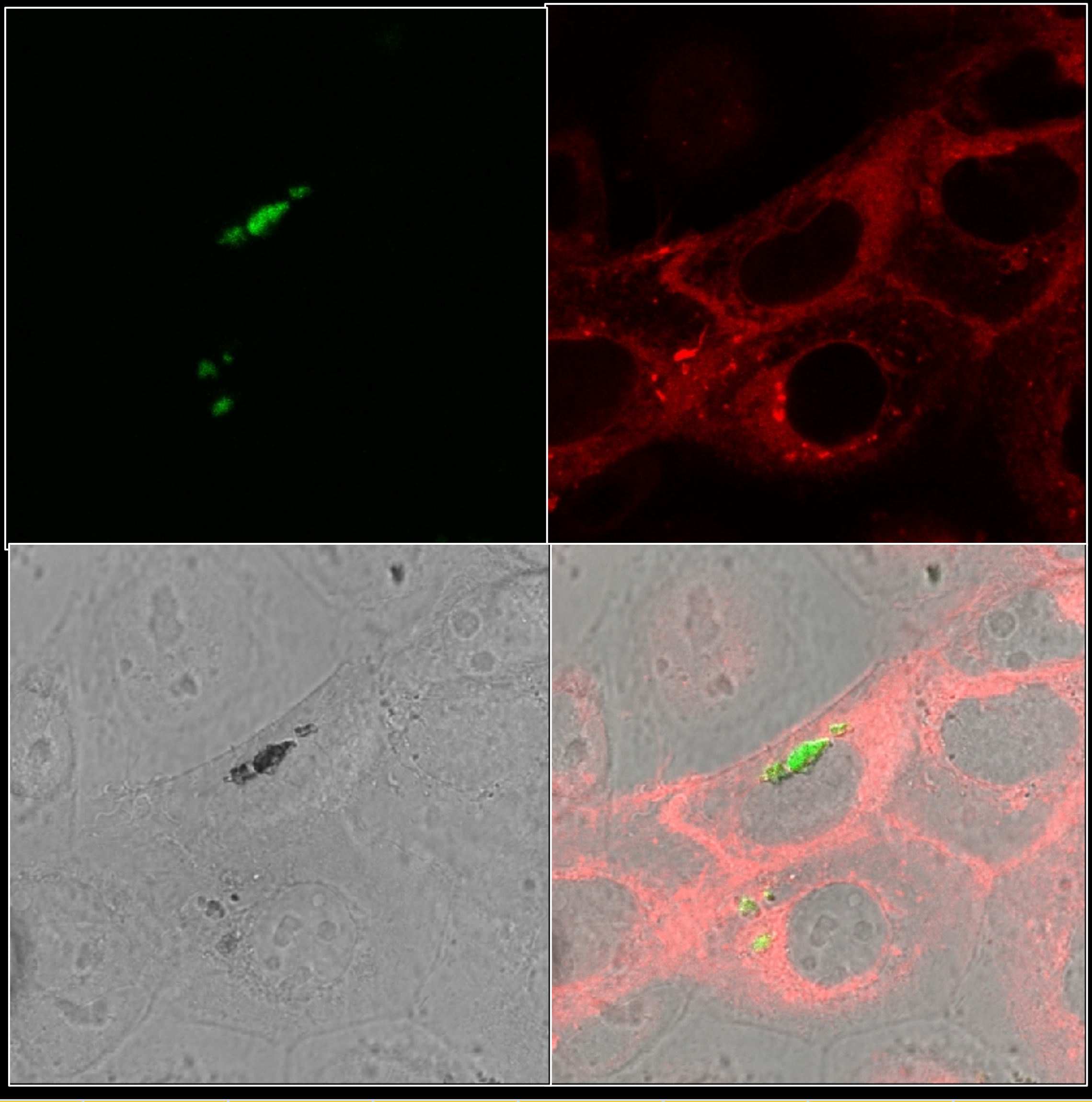

Pearsons Correlation	Colocalization Coefficient Mx	Colocalization Coefficient My	Overlap Coefficient R	Overlap Coefficient Kx	Overlap Coefficient Ky	X Min Threshold	X Max Threshold	Y Min Threshold	Y Max Threshold	Voxel Ratio Ch.X/Ch.Y	Global Pearsons Correlation
-0.271	0.401	0.266	0.199	0.479	0.11	13	255	68	255	0.801	-0.157

Colocalization Experiments AS

Dextran-QD (13)

Mitochondria

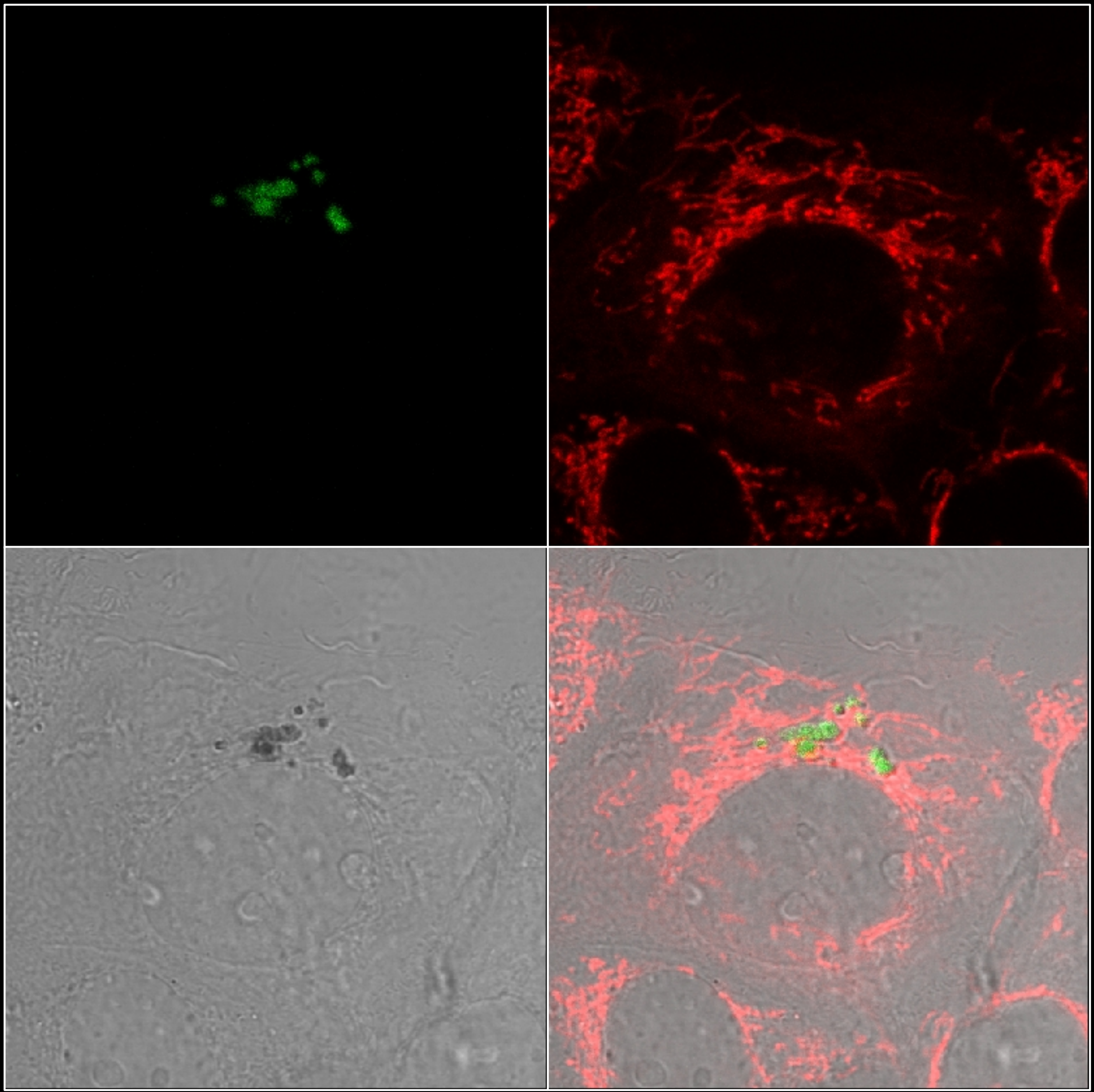

Pearsons Correlation	Colocalization Coefficient Mx	Colocalization Coefficient My	Overlap Coefficient R	Overlap Coefficient Kx	Overlap Coefficient Ky	X Min Threshold	X Max Threshold	Y Min Threshold	Y Max Threshold	Voxel Ratio Ch.X/Ch.Y	Global Pearsons Correlation
-0.134	0.214	0.109	0.099	0.469	0.024	14	255	78	255	545	-0.059

Colocalization Experiments HeLa

Gal-QD (14b)

Early Endosomes

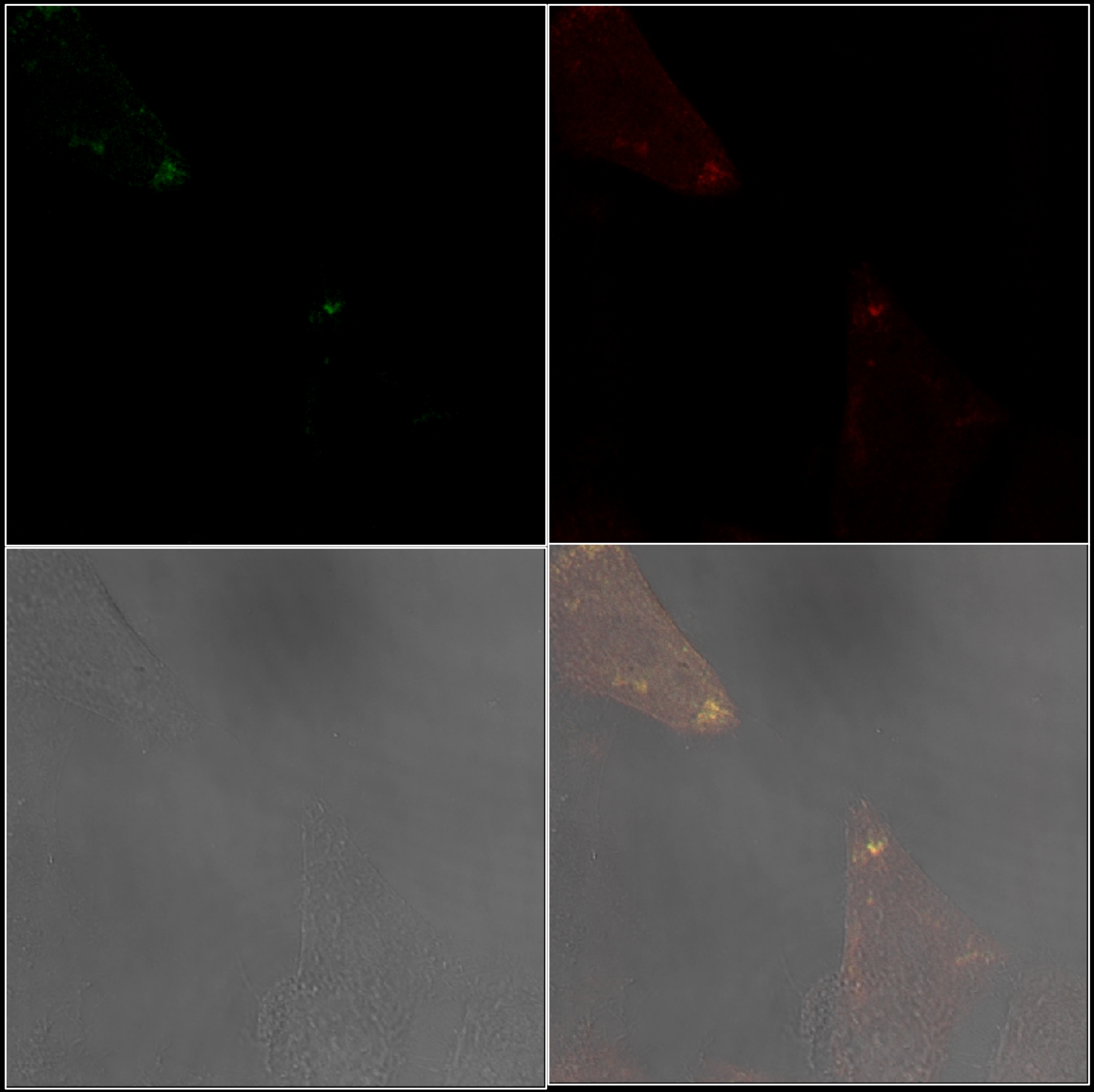

Pearsons Correlation	Colocalization Coefficient Mx	Colocalization Coefficient My	Overlap Coefficient R	Overlap Coefficient Kx	Overlap Coefficient Ky	X Min Threshold	X Max Threshold	Y Min Threshold	Y Max Threshold	Voxel Ratio Ch.X/Ch.Y	Global Pearsons Correlation
0.753	1	1	0.952	0.896	1.012	6	91	6	91	1	0.753

Colocalization Experiments HeLa

Gal-QD (14b)

Late Endosomes

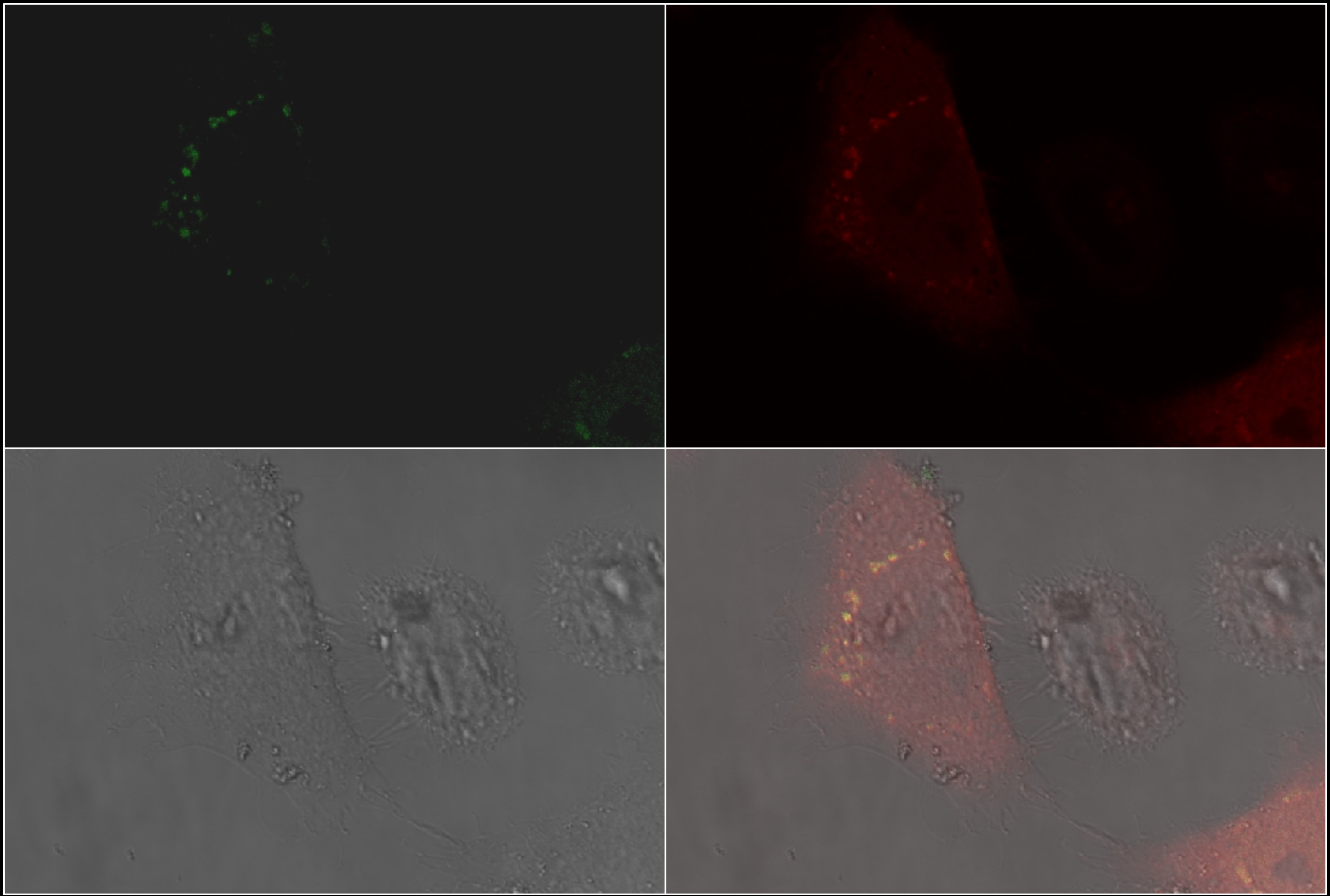

Pearsons Correlation	Colocalization Coefficient Mx	Colocalization Coefficient My	Overlap Coefficient R	Overlap Coefficient Kx	Overlap Coefficient Ky	X Min Threshold	X Max Threshold	Y Min Threshold	Y Max Threshold	Voxel Ratio Ch.X/Ch.Y	Global Pearsons Correlation
0.9027	1	1	0.91	1.697	0.505	5	203	5	203	1	0.9027

Colocalization Experiments HeLa

Gal-QD (14b)

Lysosomes

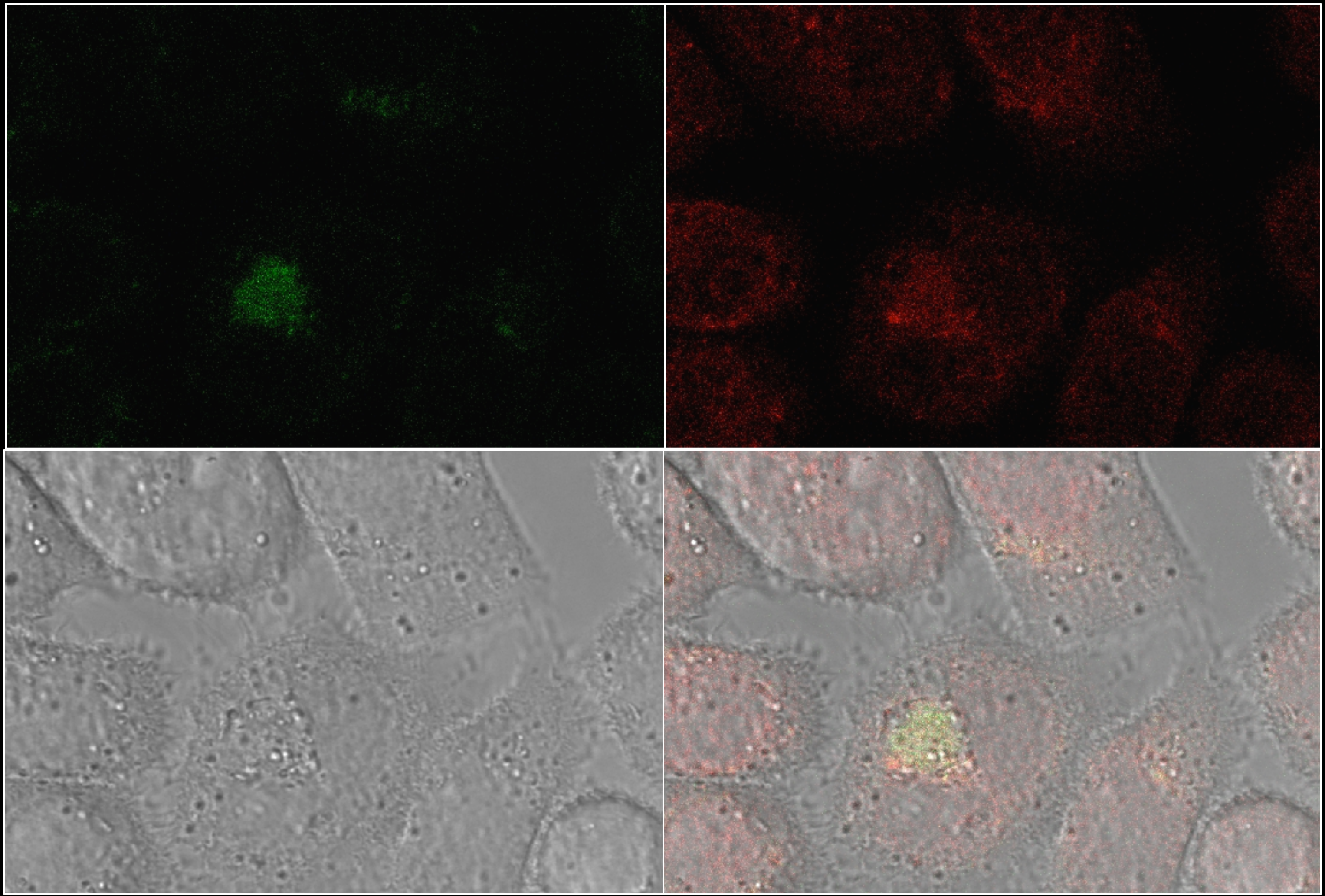

Pearsons Correlation	Colocalization Coefficient Mx	Colocalization Coefficient My	Overlap Coefficient R	Overlap Coefficient Kx	Overlap Coefficient Ky	X Min Threshold	X Max Threshold	Y Min Threshold	Y Max Threshold	Voxel Ratio Ch.X/Ch.Y	Global Pearsons Correlation
0.095	0.3195	0.444	0.339	0.135	0.899	26	227	9	227	1.42	0.204

Colocalization Experiments HeLa

Gal-QD (14b)

ER

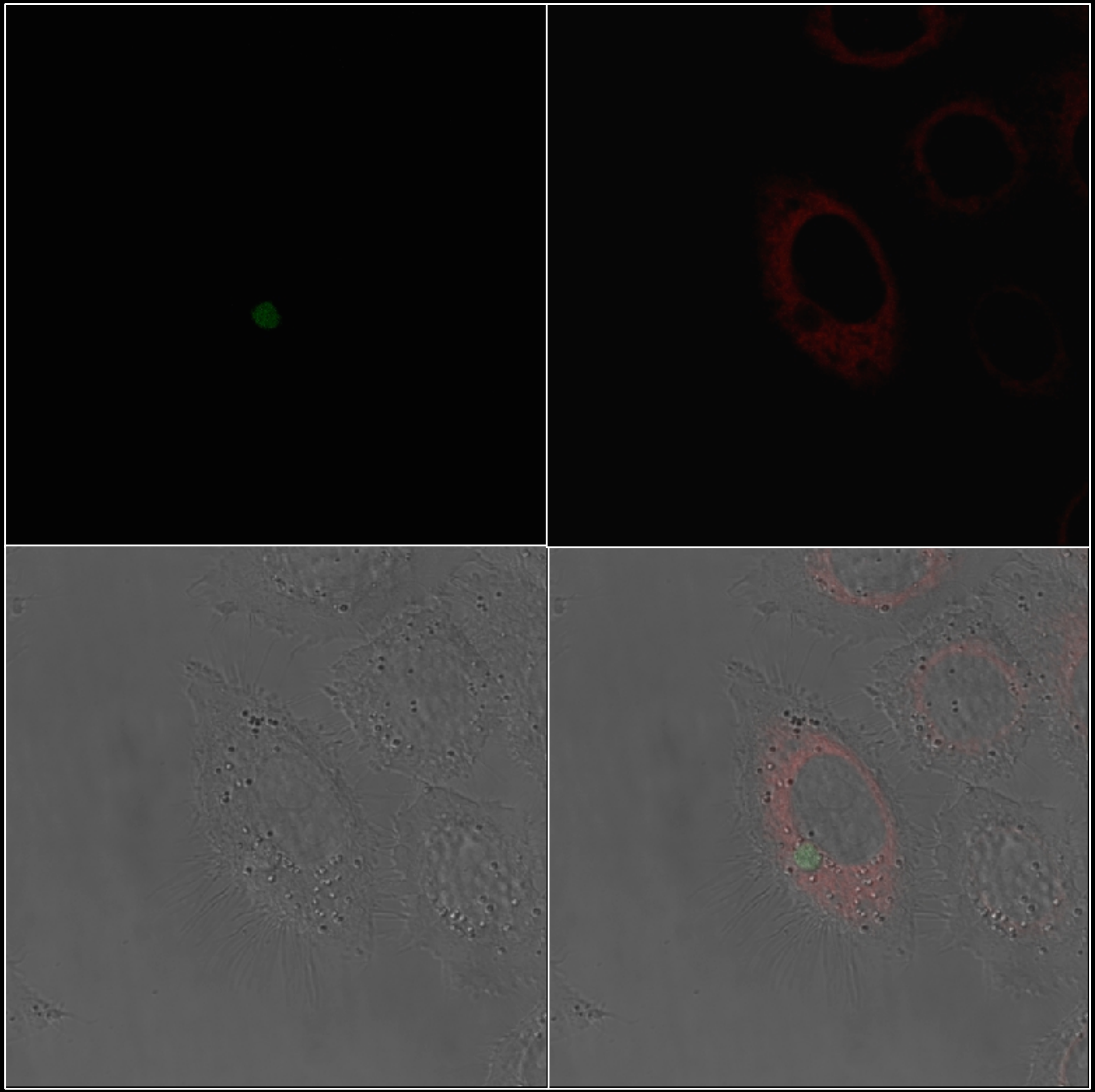

Pearsons Correlation	Colocalization Coefficient Mx	Colocalization Coefficient My	Overlap Coefficient R	Overlap Coefficient Kx	Overlap Coefficient Ky	X Min Threshold	X Max Threshold	Y Min Threshold	Y Max Threshold	Voxel Ratio Ch.X/Ch.Y	Global Pearsons Correlation
-0.124	0.6635	1	0.578	0.774	0.448	7	68	5	68	2.075	-0.0725

Colocalization Experiments HeLa

Gal-QD (14b)

Golgi

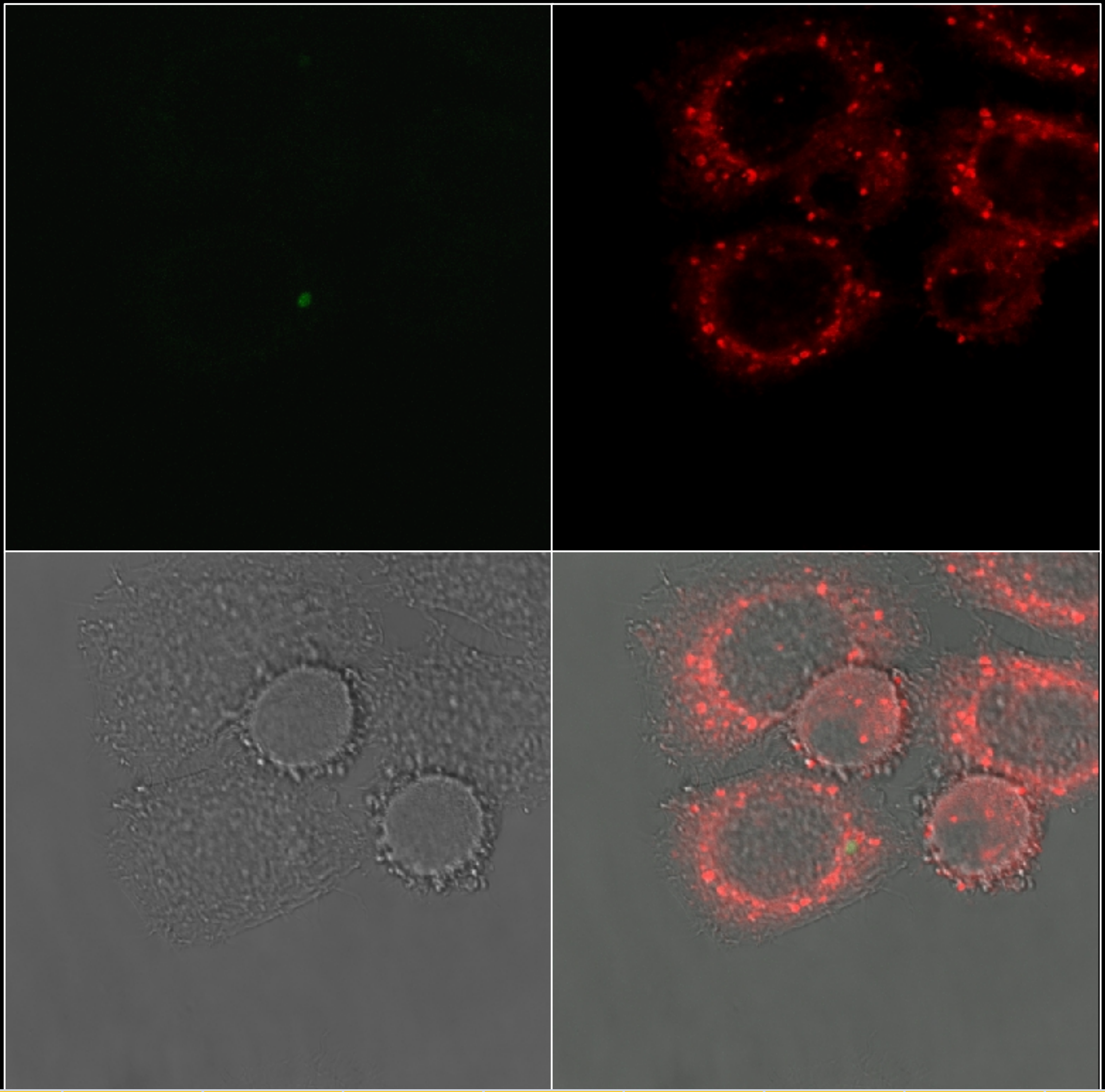

Pearsons Correlation	Colocalization Coefficient Mx	Colocalization Coefficient My	Overlap Coefficient R	Overlap Coefficient Kx	Overlap Coefficient Ky	X Min Threshold	X Max Threshold	Y Min Threshold	Y Max Threshold	Voxel Ratio Ch.X/Ch.Y	Global Pearsons Correlation
-0.0115	0.9875	1	0.694	3.06	0.1605	8	156	3	255	1.015	-0.011

Colocalization Experiments HeLa

Gal-QD (14b)

Mitochondria

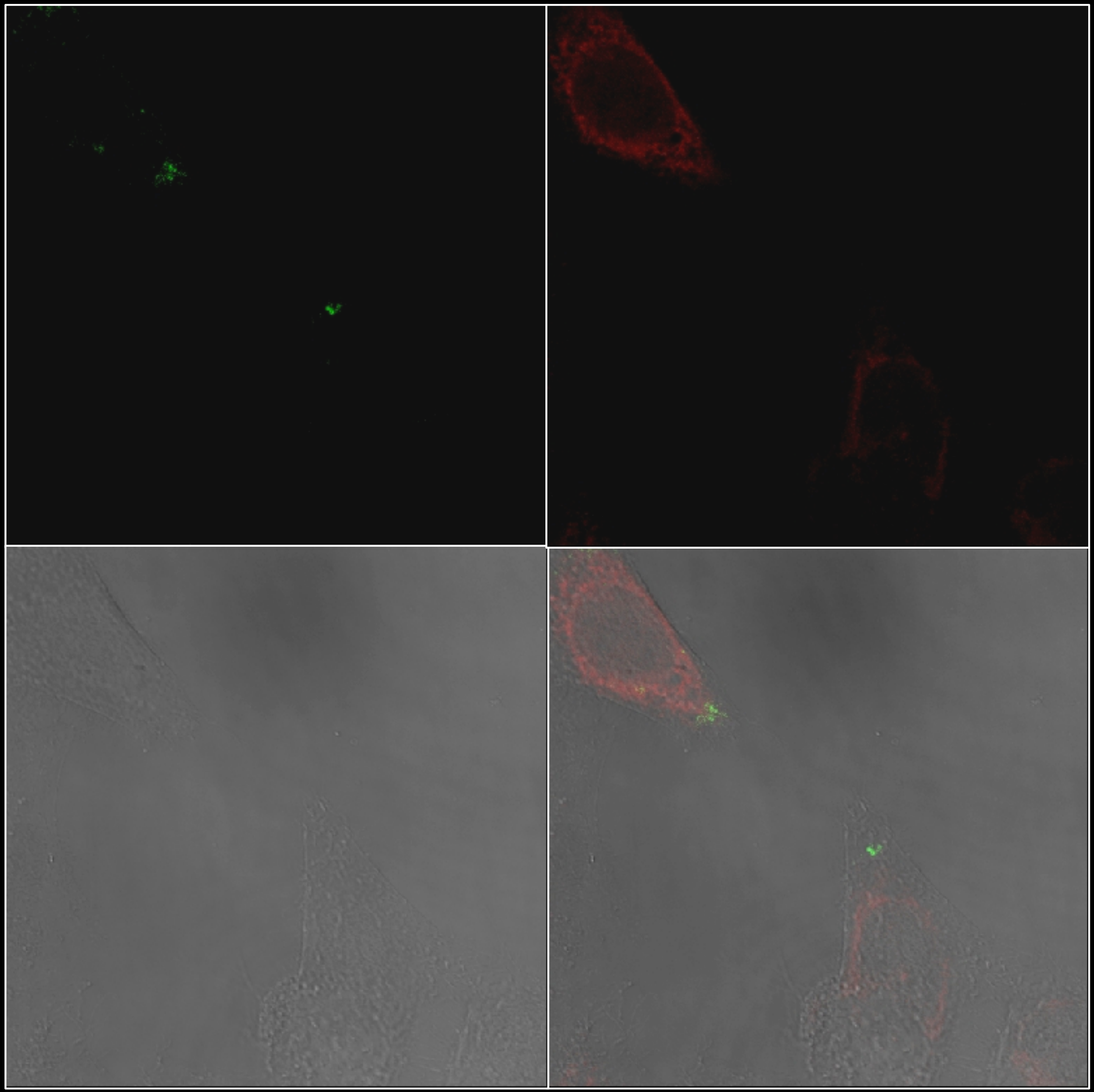

Pearsons Correlation	Colocalization Coefficient Mx	Colocalization Coefficient My	Overlap Coefficient R	Overlap Coefficient Kx	Overlap Coefficient Ky	X Min Threshold	X Max Threshold	Y Min Threshold	Y Max Threshold	Voxel Ratio Ch.X/Ch.Y	Global Pearsons Correlation
0.382	0.887	1	0.861	0.789	0.982	6	91	7	91	1.207	0.386

Colocalization Experiments AS

Gal-QD (14b)

Early Endosomes

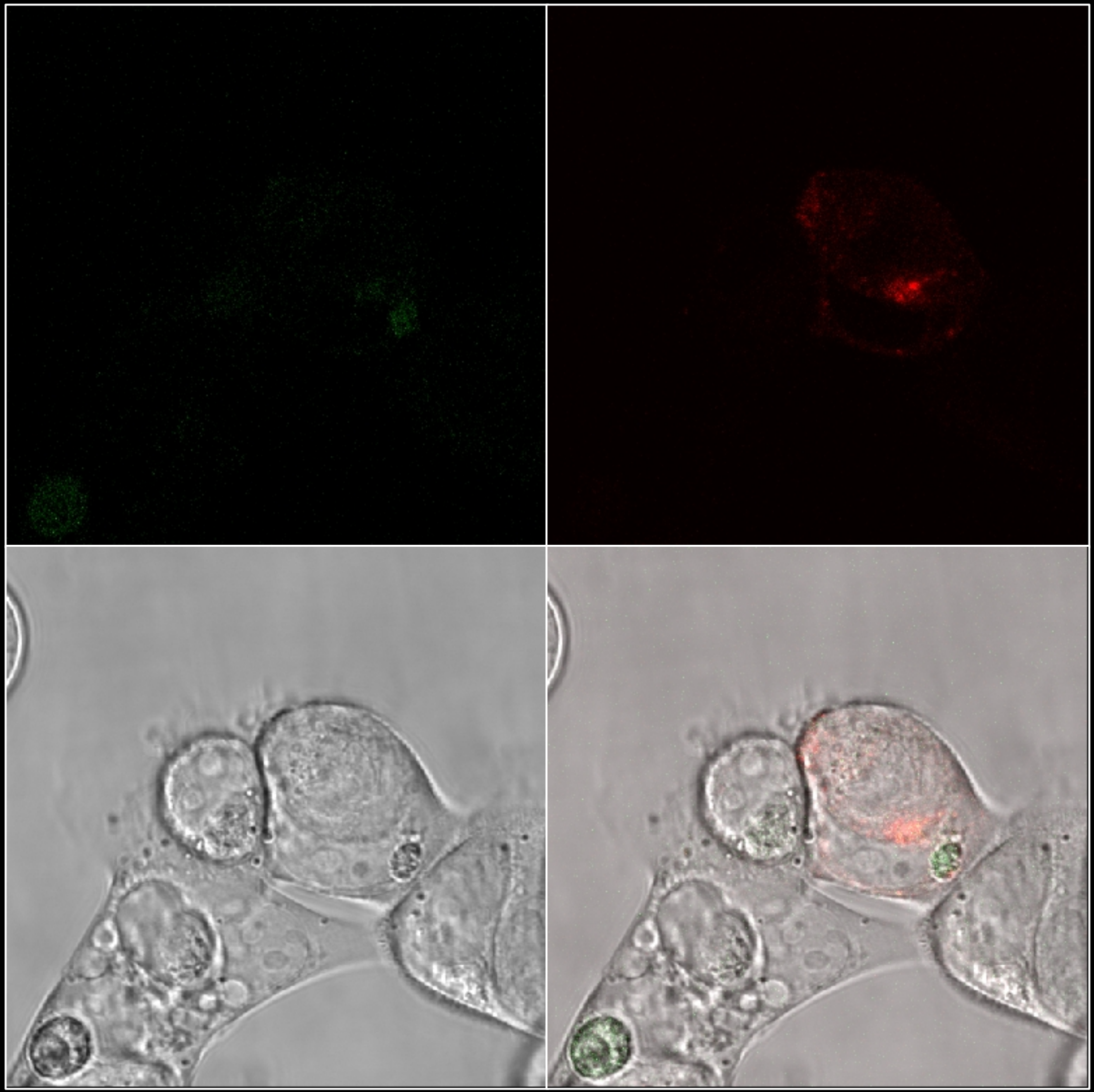

Pearsons Correlation	Colocalization Coefficient Mx	Colocalization Coefficient My	Overlap Coefficient R	Overlap Coefficient Kx	Overlap Coefficient Ky	X Min Threshold	X Max Threshold	Y Min Threshold	Y Max Threshold	Voxel Ratio Ch.X/Ch.Y	Global Pearsons Correlation
0.1825	1	1	0.629	1.2675	0.345	6	255	6	255	1	0.1825

Colocalization Experiments AS

Gal-QD (14b)

Late Endosomes

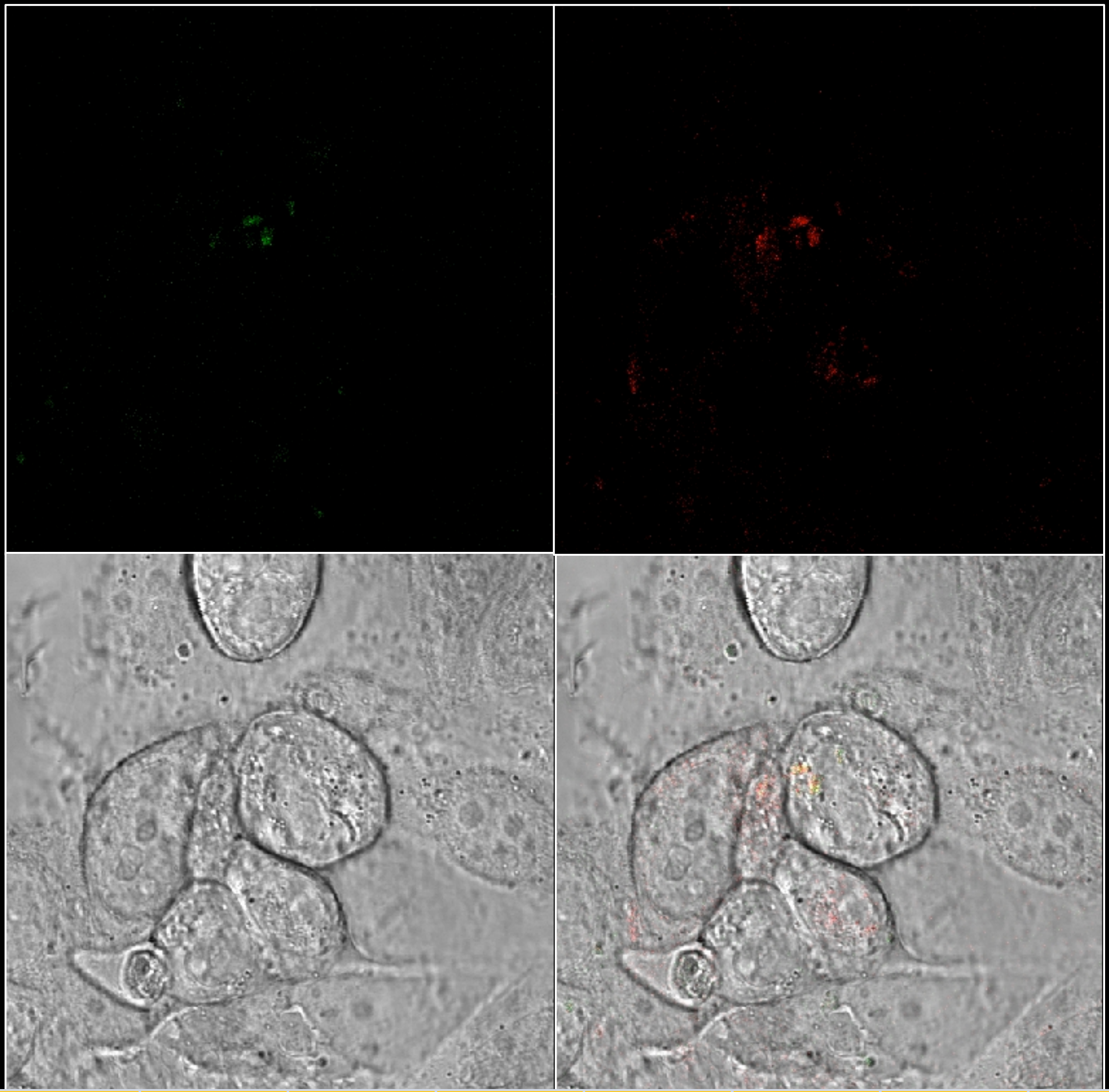

Pearsons Correlation	Colocalization Coefficient Mx	Colocalization Coefficient My	Overlap Coefficient R	Overlap Coefficient Kx	Overlap Coefficient Ky	X Min Threshold	X Max Threshold	Y Min Threshold	Y Max Threshold	Voxel Ratio Ch.X/Ch.Y	Global Pearsons Correlation
0.283	0.3855	0.993	0.4765	0.7335	0.328	8	255	3	255	4.66	0.281

Colocalization Experiments AS

Gal-QD (14b)

Lysosomes

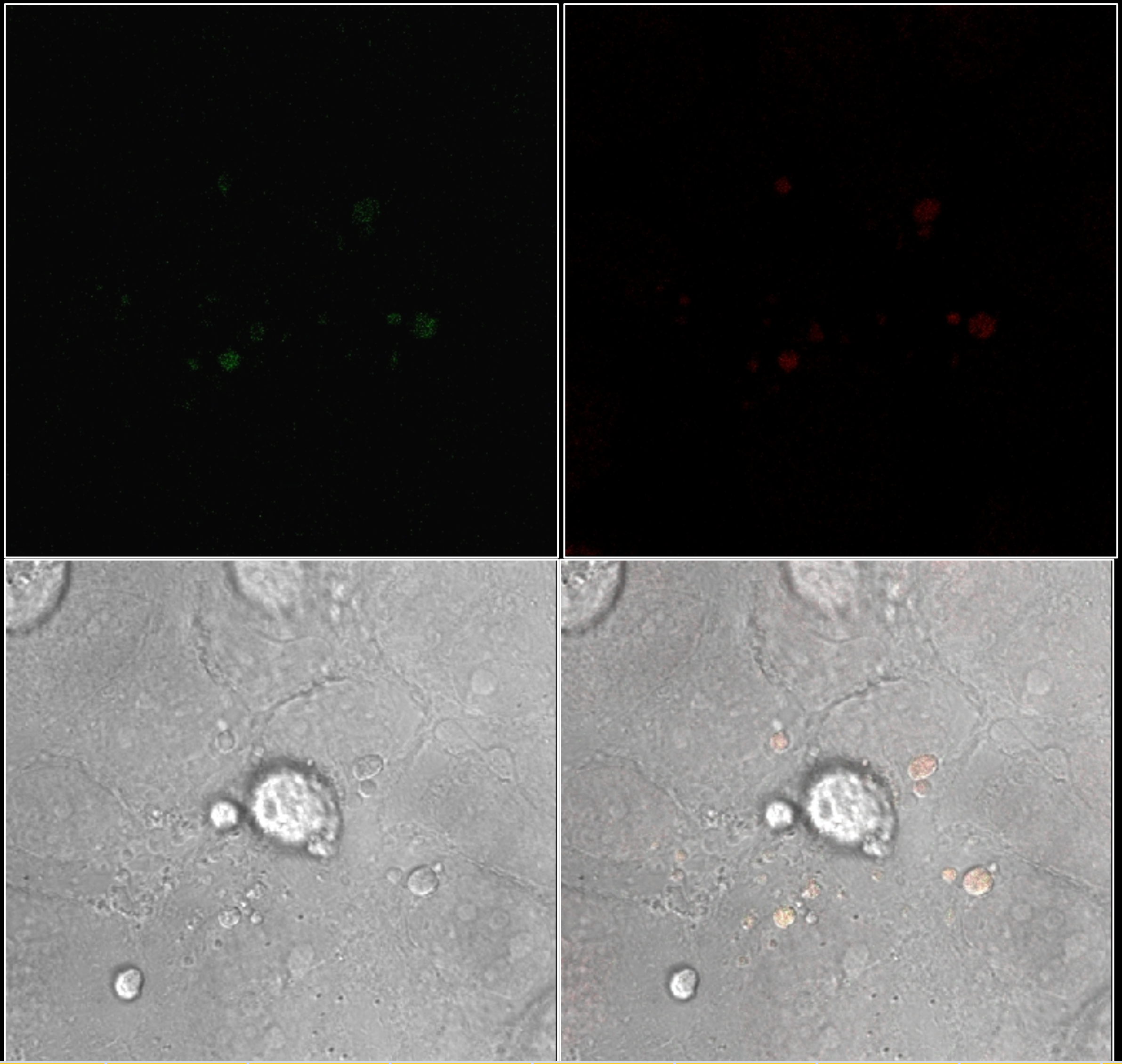

Pearsons Correlation	Colocalization Coefficient Mx	Colocalization Coefficient My	Overlap Coefficient R	Overlap Coefficient Kx	Overlap Coefficient Ky	X Min Threshold	X Max Threshold	Y Min Threshold	Y Max Threshold	Voxel Ratio Ch.X/Ch.Y	Global Pearsons Correlation
0.412	0.676	1	0.698	1.163	0.42	5	184	8	184	1.718	0.412

Colocalization Experiments AS

Gal-QD (14b)

ER

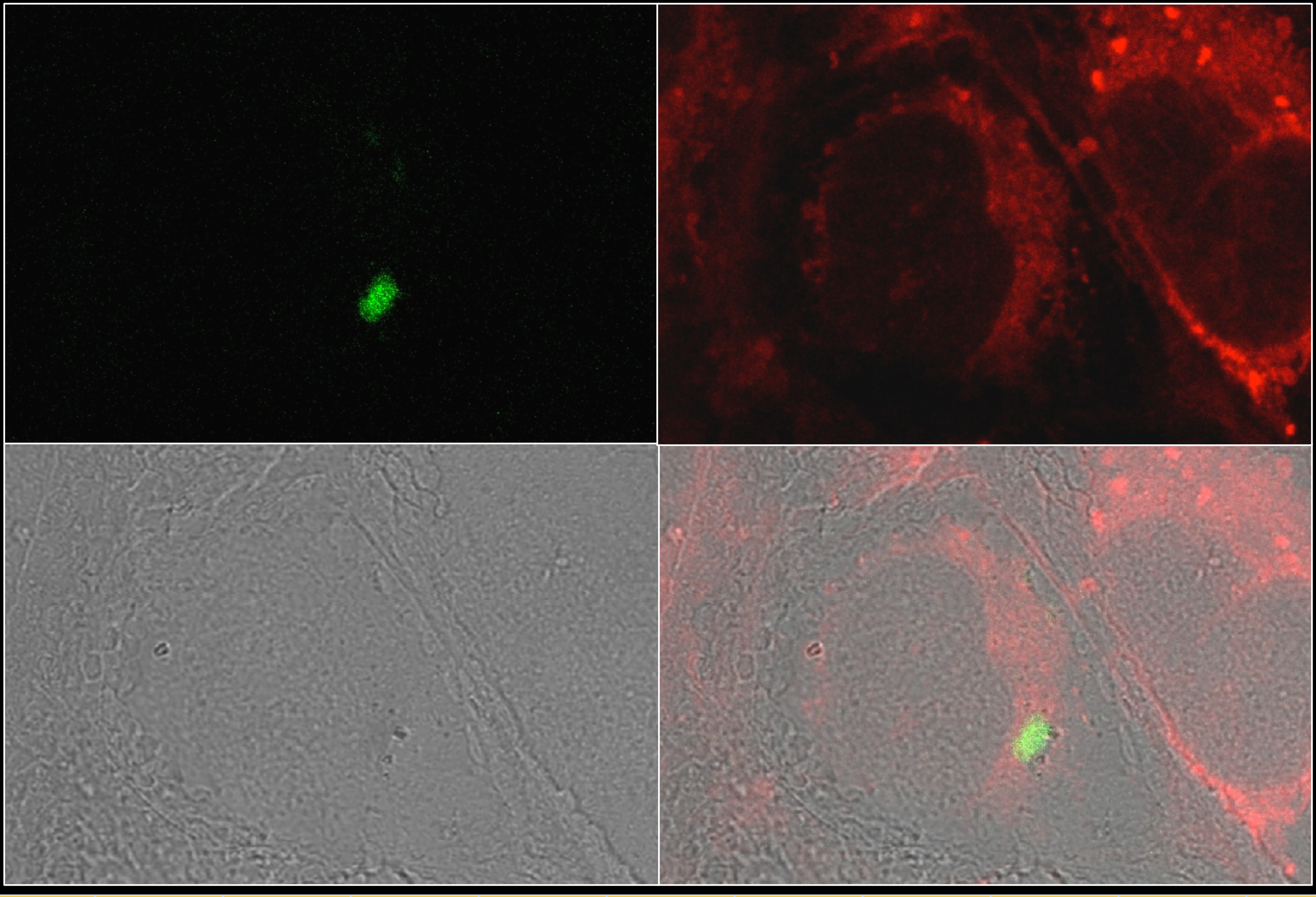

Pearsons Correlation	Colocalization Coefficient Mx	Colocalization Coefficient My	Overlap Coefficient R	Overlap Coefficient Kx	Overlap Coefficient Ky	X Min Threshold	X Max Threshold	Y Min Threshold	Y Max Threshold	Voxel Ratio Ch.X/Ch.Y	Global Pearsons Correlation
-0.0795	0.4175	0.13275	0.2025	1.02	0.04525	24	250	48	250	0.3095	-0.11395

Colocalization Experiments AS

Gal-QD (14b)

Golgi

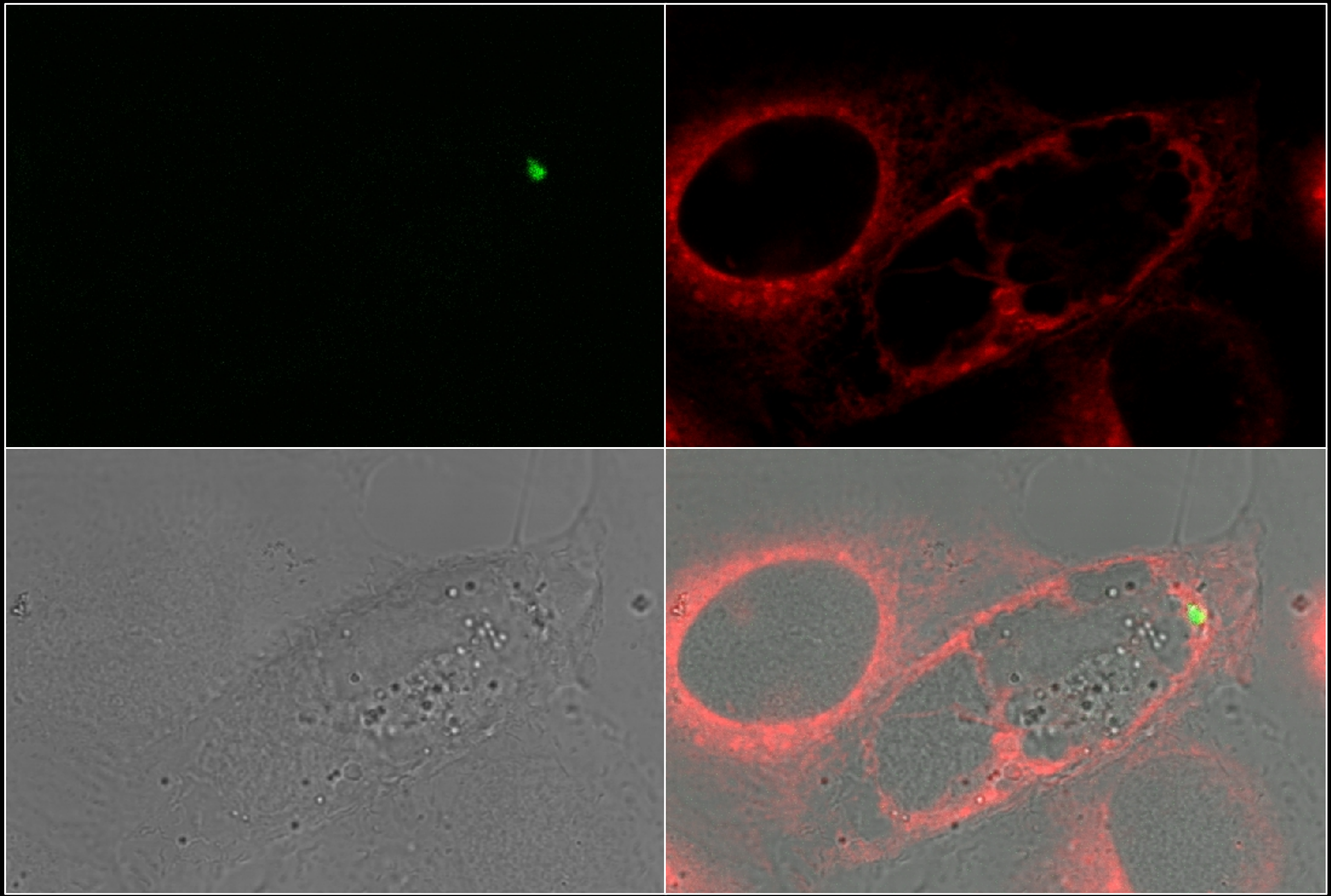

Pearsons Correlation	Colocalization Coefficient Mx	Colocalization Coefficient My	Overlap Coefficient R	Overlap Coefficient Kx	Overlap Coefficient Ky	X Min Threshold	X Max Threshold	Y Min Threshold	Y Max Threshold	Voxel Ratio Ch.X/Ch.Y	Global Pearsons Correlation
-0.0035	1	1	0.47	1.563	0.258	2	255	2	255	1	-0.0035

Colocalization Experiments AS

Mitochondria

Gal-QD (14b)

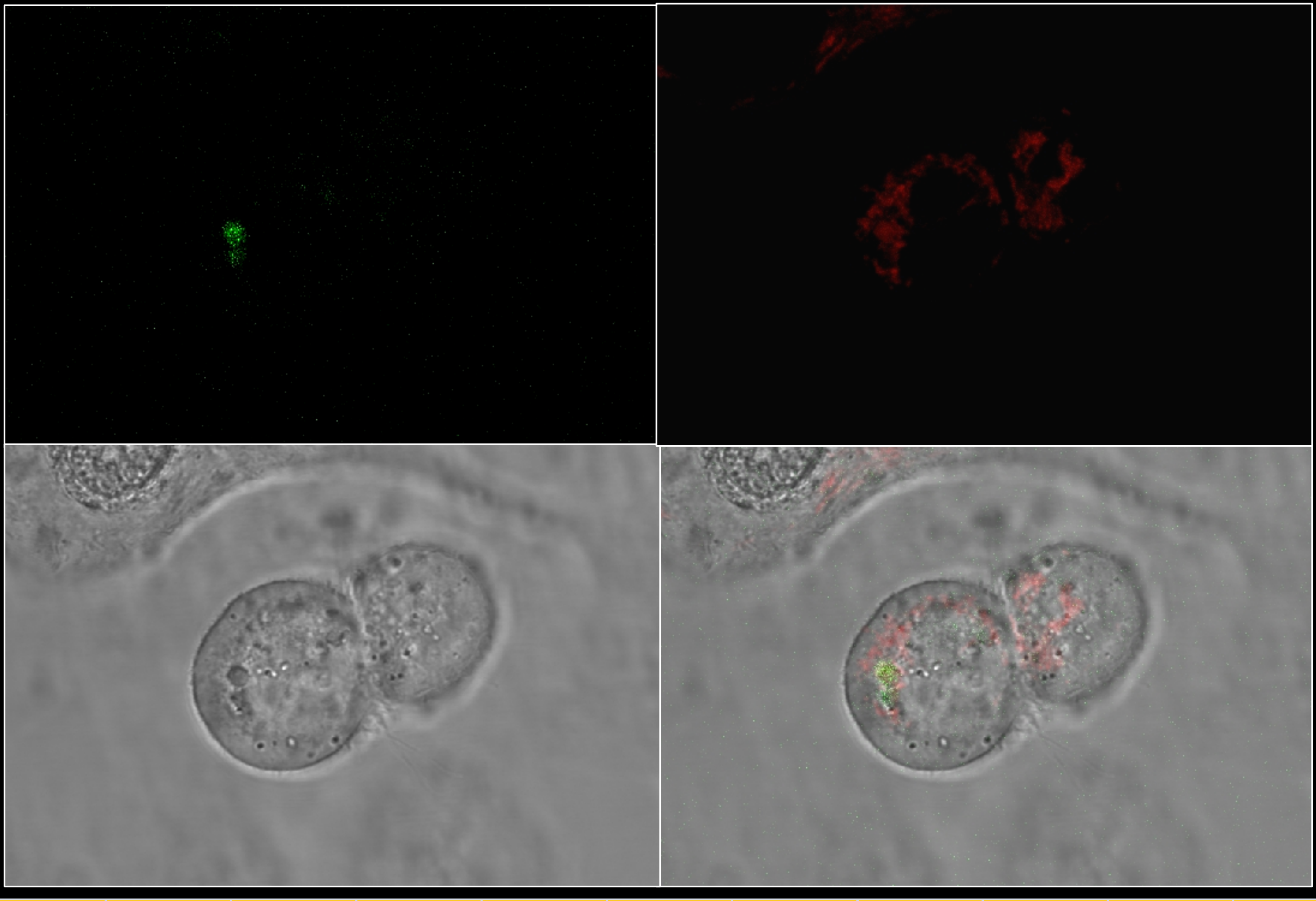

Pearsons Correlation	Colocalization Coefficient Mx	Colocalization Coefficient My	Overlap Coefficient R	Overlap Coefficient Kx	Overlap Coefficient Ky	X Min Threshold	X Max Threshold	Y Min Threshold	Y Max Threshold	Voxel Ratio Ch.X/Ch.Y	Global Pearsons Correlation
0.2565	0.5285	0.08635	0.2095	0.852	0.0518	10	91	10	91	0.166	0.16945

Colocalization Experiments HeLa

Lactose-QD (17b)

Early Endosomes

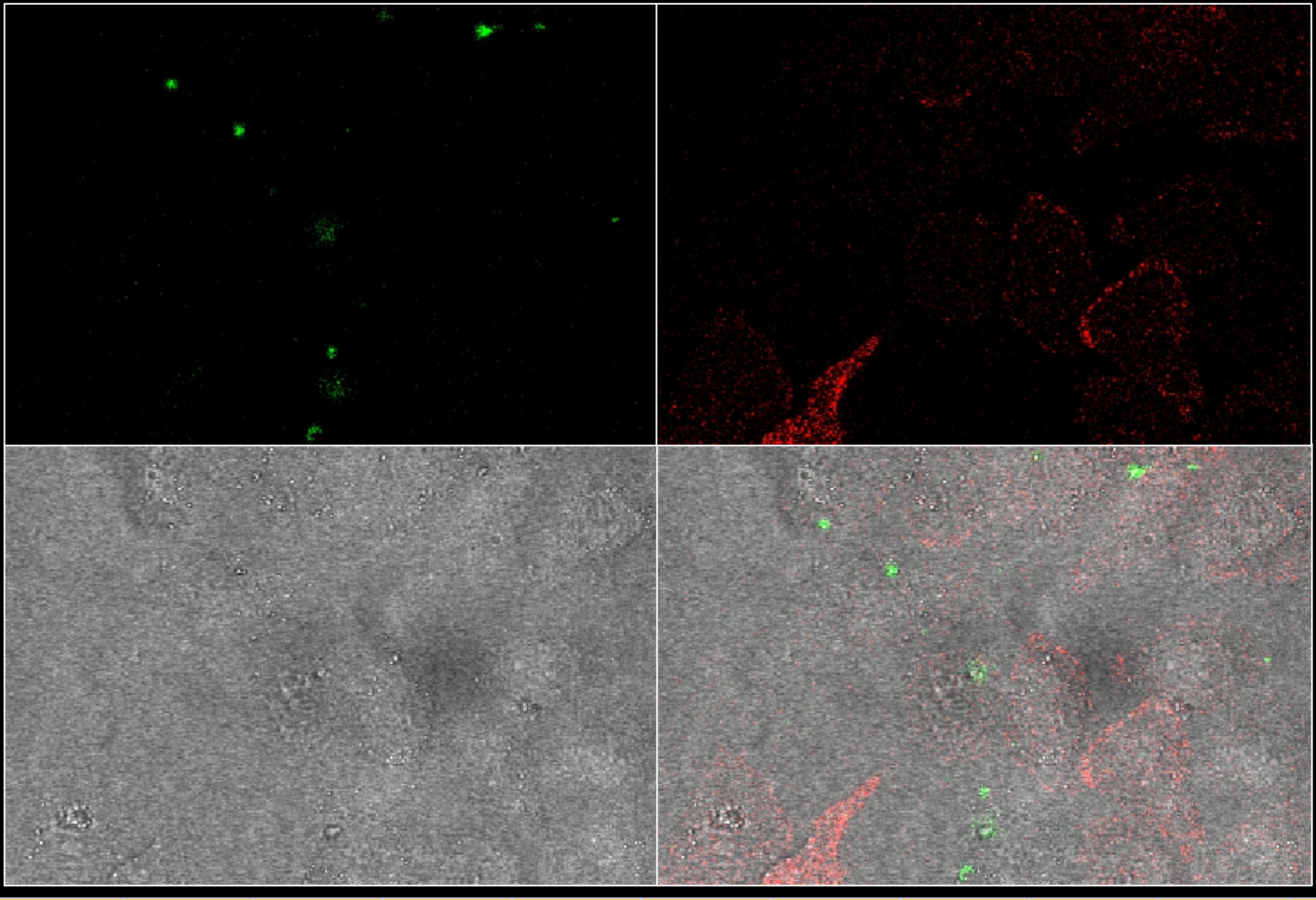

Pearsons Correlation	Colocalization Coefficient Mx	Colocalization Coefficient My	Overlap Coefficient R	Overlap Coefficient Kx	Overlap Coefficient Ky	X Min Threshold	X Max Threshold	Y Min Threshold	Y Max Threshold	Voxel Ratio Ch.X/Ch.Y	Global Pearsons Correlation
-0.05205	0.1095	0.0995	0.0668	0.05155	0.0867	17	255	11	255	1.405	0.05075

Colocalization Experiments HeLa

Lactose-QD (17b)

Late Endosomes

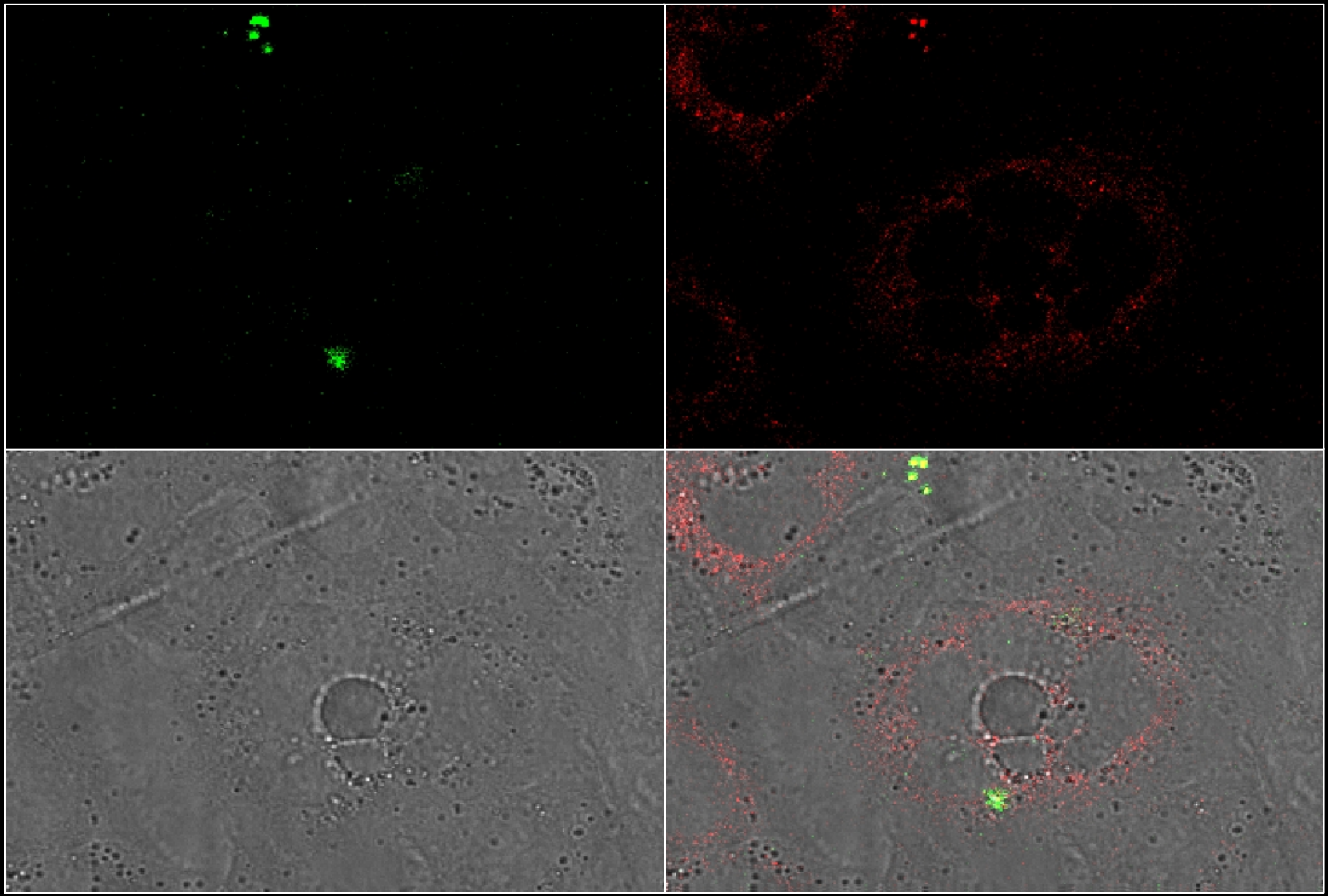

Pearsons Correlation	Colocalization Coefficient Mx	Colocalization Coefficient My	Overlap Coefficient R	Overlap Coefficient Kx	Overlap Coefficient Ky	X Min Threshold	X Max Threshold	Y Min Threshold	Y Max Threshold	Voxel Ratio Ch.X/Ch.Y	Global Pearsons Correlation
0.4465	0.03995	0.4445	0.1785	0.0698	0.474	17	255	15	255	9.85	0.1581

Colocalization Experiments HeLa

Lactose-QD (17b)

Late Endosomes

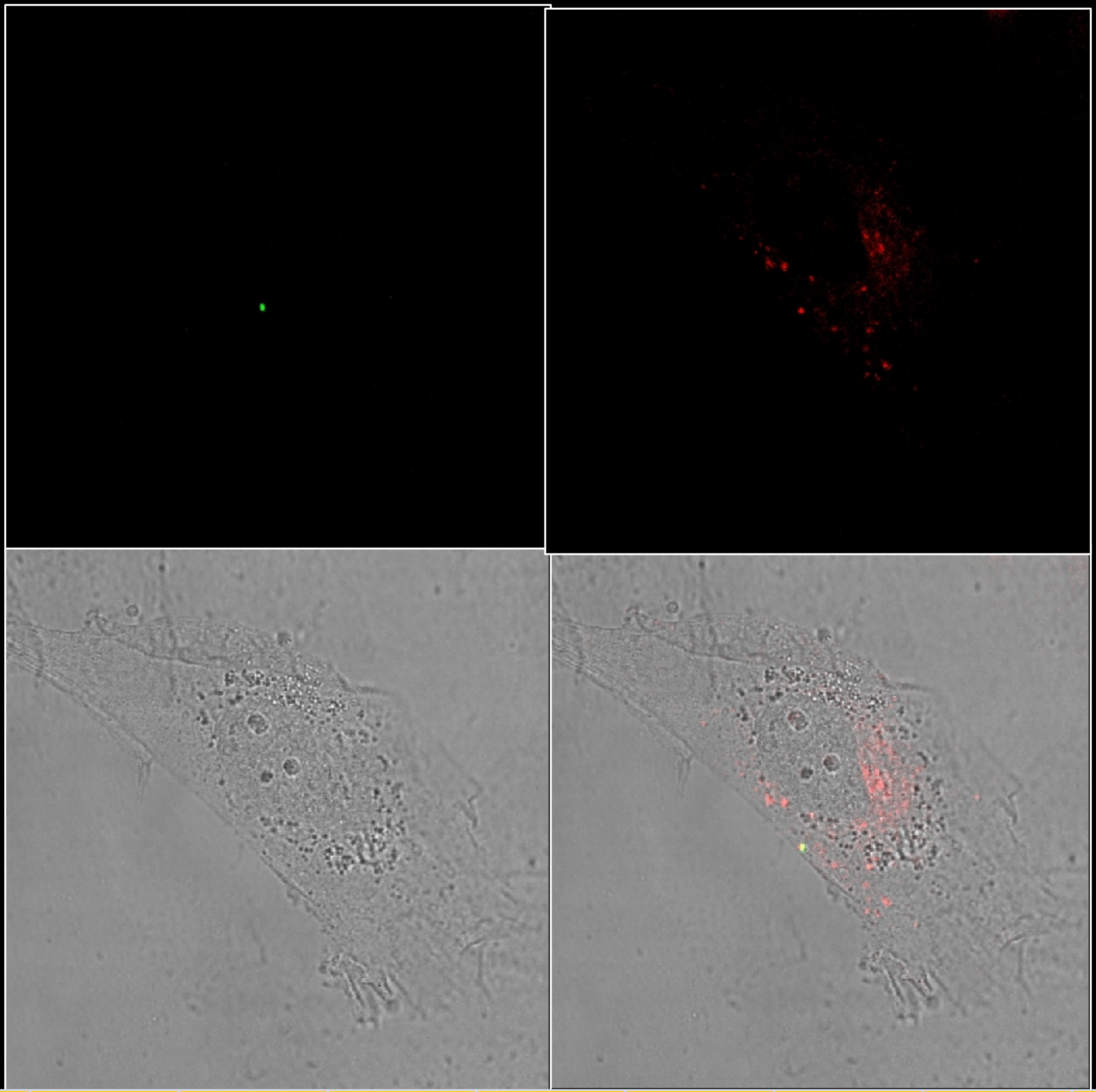

Pearsons Correlation	Colocalization Coefficient Mx	Colocalization Coefficient My	Overlap Coefficient R	Overlap Coefficient Kx	Overlap Coefficient Ky	X Min Threshold	X Max Threshold	Y Min Threshold	Y Max Threshold	Voxel Ratio Ch.X/Ch.Y	Global Pearsons Correlation
0.695	0.176	0.0389	0.146	0.566	0.0377	11	255	23	255	0.246	0.129

Colocalization Experiments HeLa

Lactose-QD (17b)

Late Endosomes 4 C

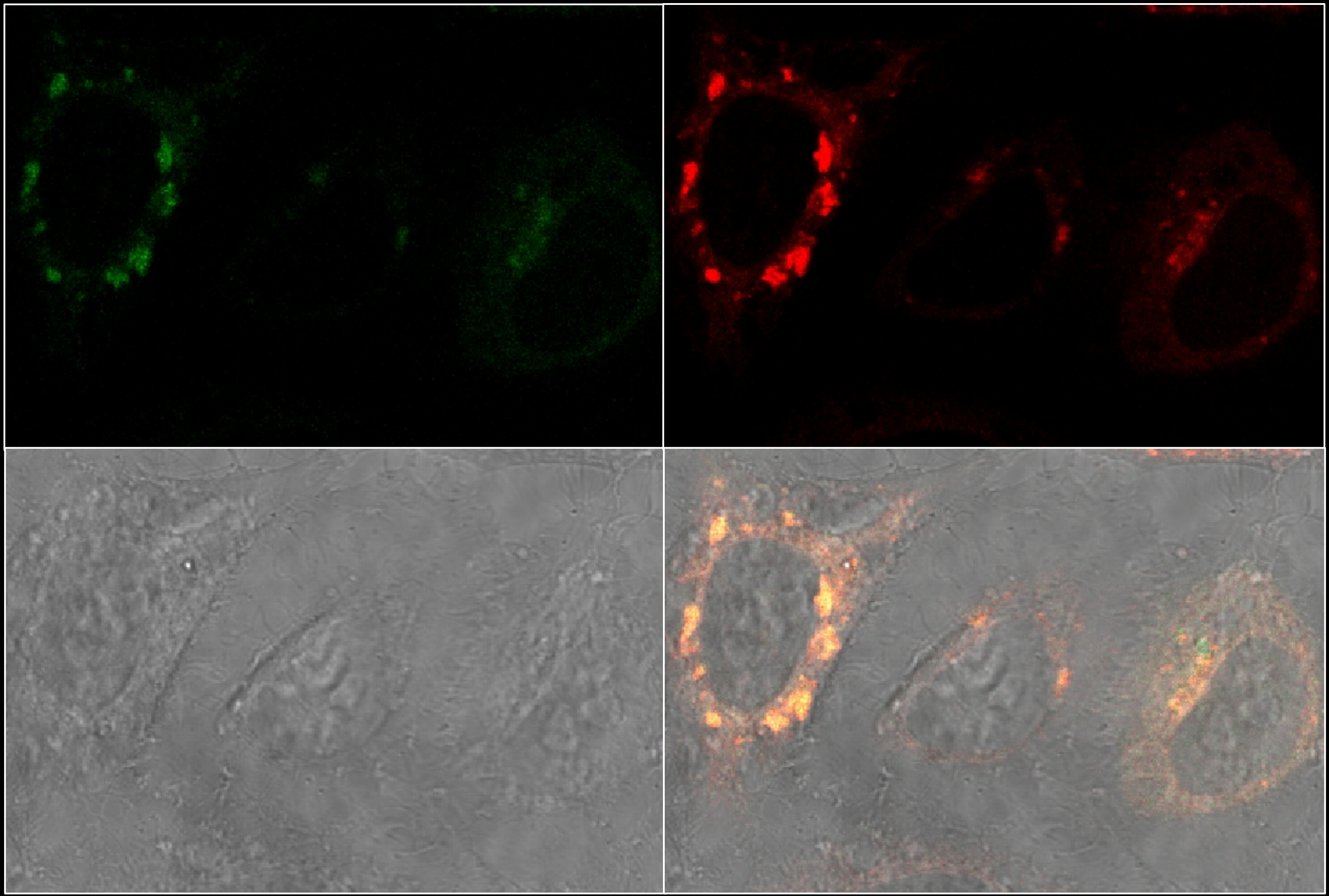

Pearsons Correlation	Colocalization Coefficient Mx	Colocalization Coefficient My	Overlap Coefficient R	Overlap Coefficient Kx	Overlap Coefficient Ky	X Min Threshold	X Max Threshold	Y Min Threshold	Y Max Threshold	Voxel Ratio Ch.X/Ch.Y	Global Pearsons Correlation
0.693	1	1	0.745	2.393	0.241	0	255	0	255	1	0.693

Colocalization Experiments HeLa

Lactose-QD (17b)

Lysosomes

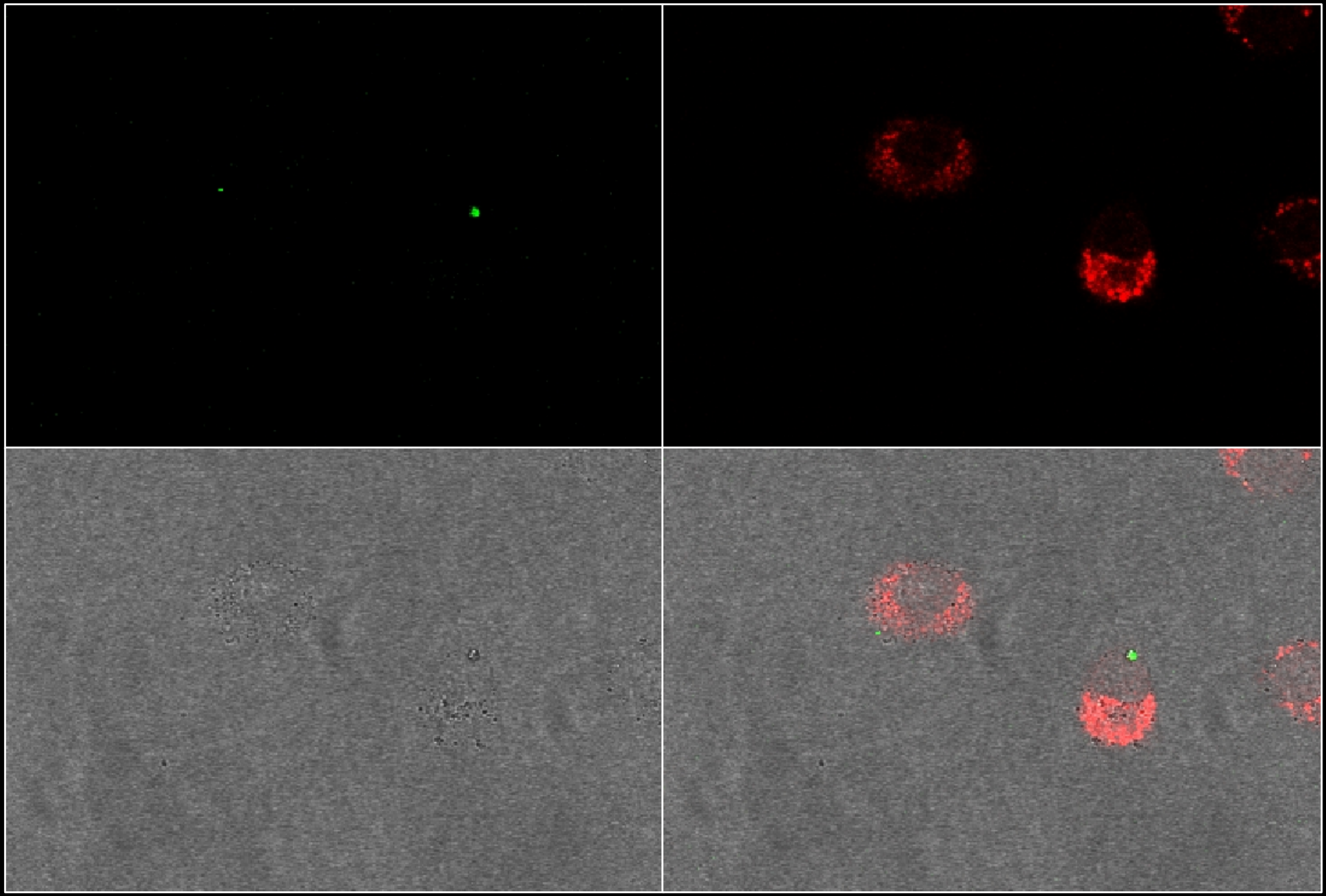

Pearsons Correlation	Colocalization Coefficient Mx	Colocalization Coefficient My	Overlap Coefficient R	Overlap Coefficient Kx	Overlap Coefficient Ky	X Min Threshold	X Max Threshold	Y Min Threshold	Y Max Threshold	Voxel Ratio Ch.X/Ch.Y	Global Pearsons Correlation
-0.1609	0.2665	0.10015	0.08825	0.454	0.01715	9	255	38	255	0.319	0.06

Colocalization Experiments HeLa

Lactose-QD (17b)

Nucleus

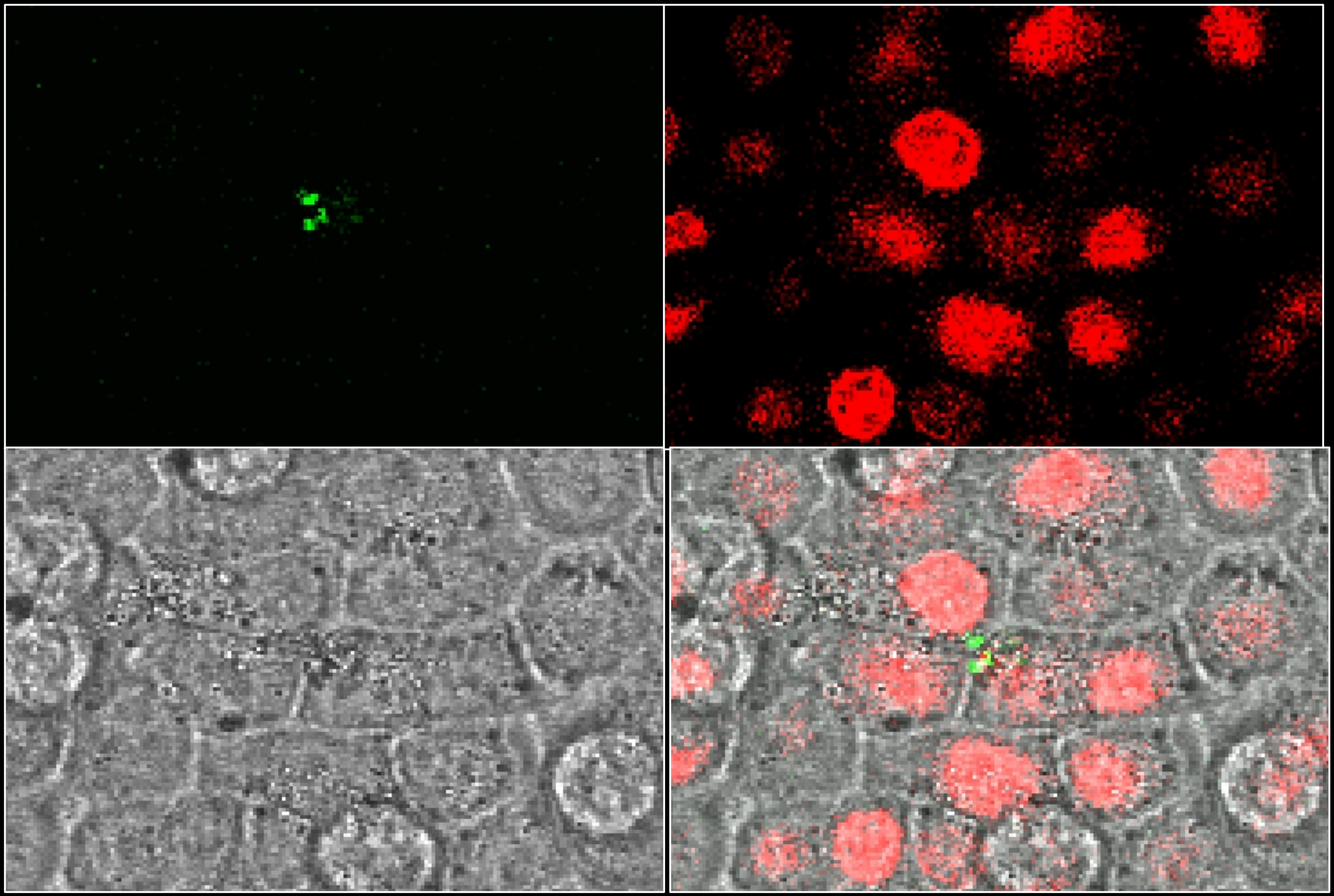

Colocalization Experiments HeLa

Lactose-QD (17b)

ER 37C

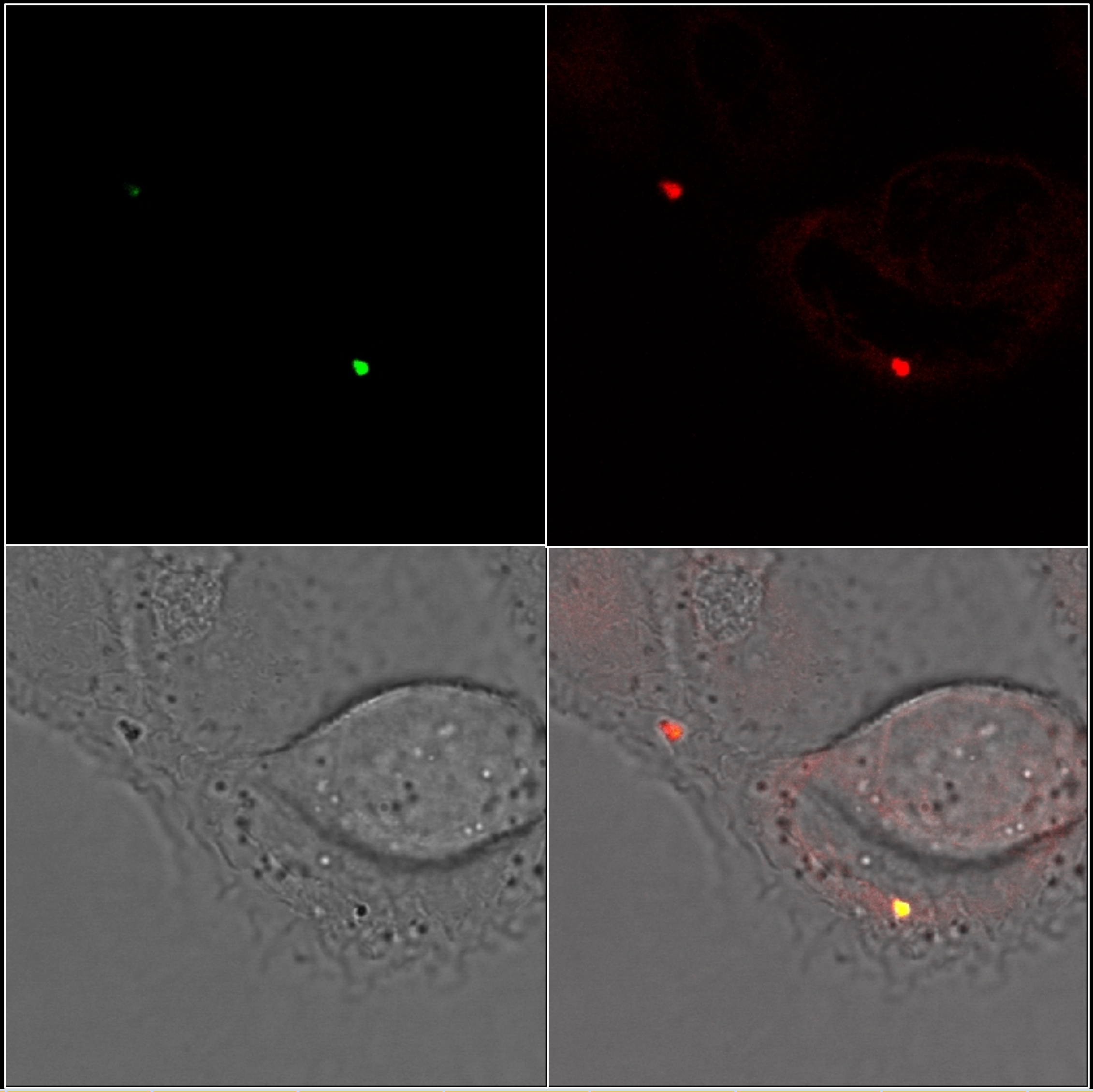

Pearsons Correlation	Colocalization Coefficient Mx	Colocalization Coefficient My	Overlap Coefficient R	Overlap Coefficient Kx	Overlap Coefficient Ky	X Min Threshold	X Max Threshold	Y Min Threshold	Y Max Threshold	Voxel Ratio Ch.X/Ch.Y	Global Pearsons Correlation
0.838	0.777	1	0.892	1.35	0.633	1.333	255	1	255	1.963	0.87

Colocalization Experiments HeLa

Lactose-QD (17b)

ER 37C

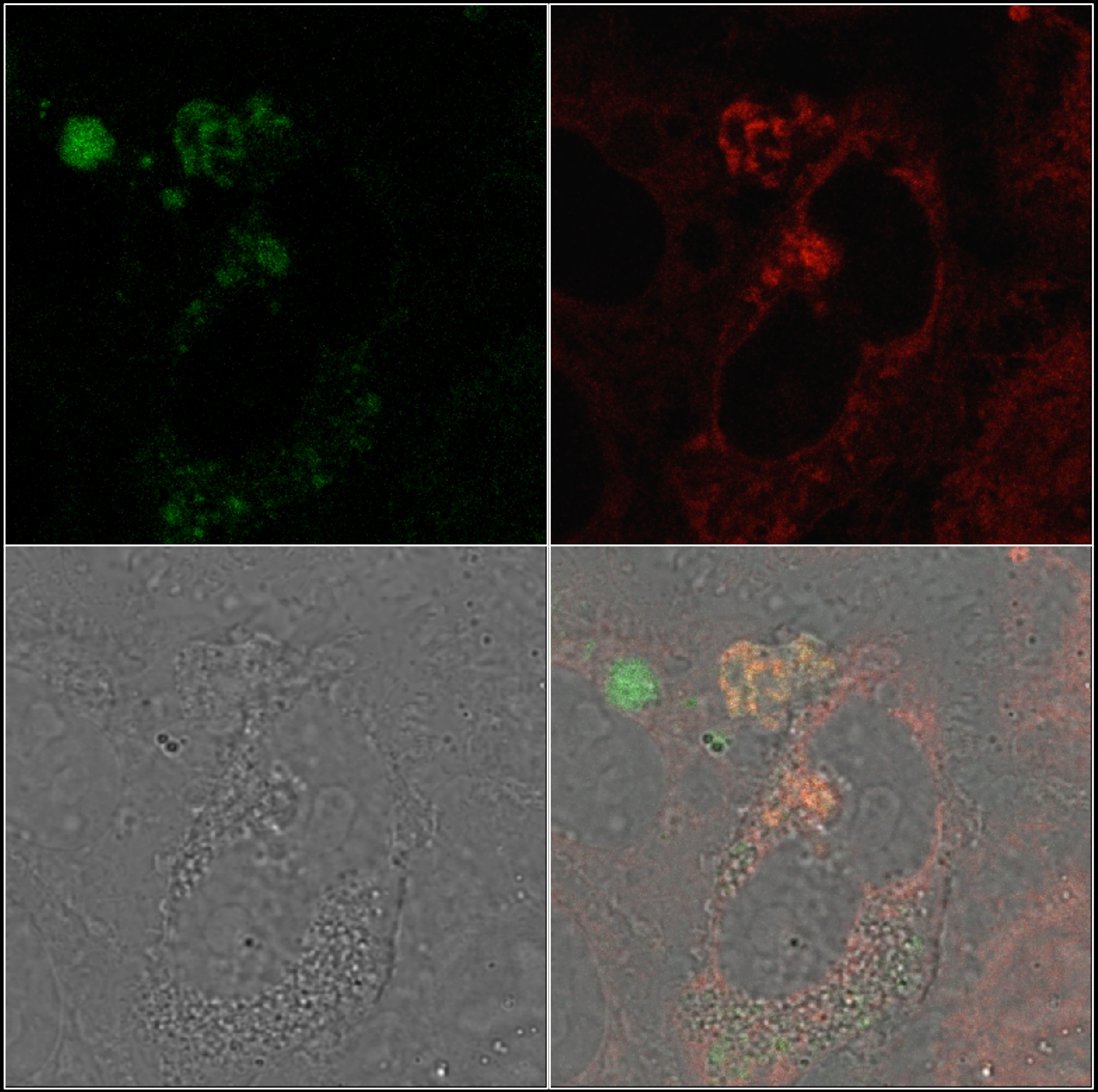

Pearsons Correlation	Colocalization Coefficient Mx	Colocalization Coefficient My	Overlap Coefficient R	Overlap Coefficient Kx	Overlap Coefficient Ky	X Min Threshold	X Max Threshold	Y Min Threshold	Y Max Threshold	Voxel Ratio Ch.X/Ch.Y	Global Pearsons Correlation
0.309	0.74	0.643	0.616	5.227	0.074	2	171	13	171	0.833	0.387

Colocalization Experiments HeLa

Lactose-QD (17b)

ER 4C

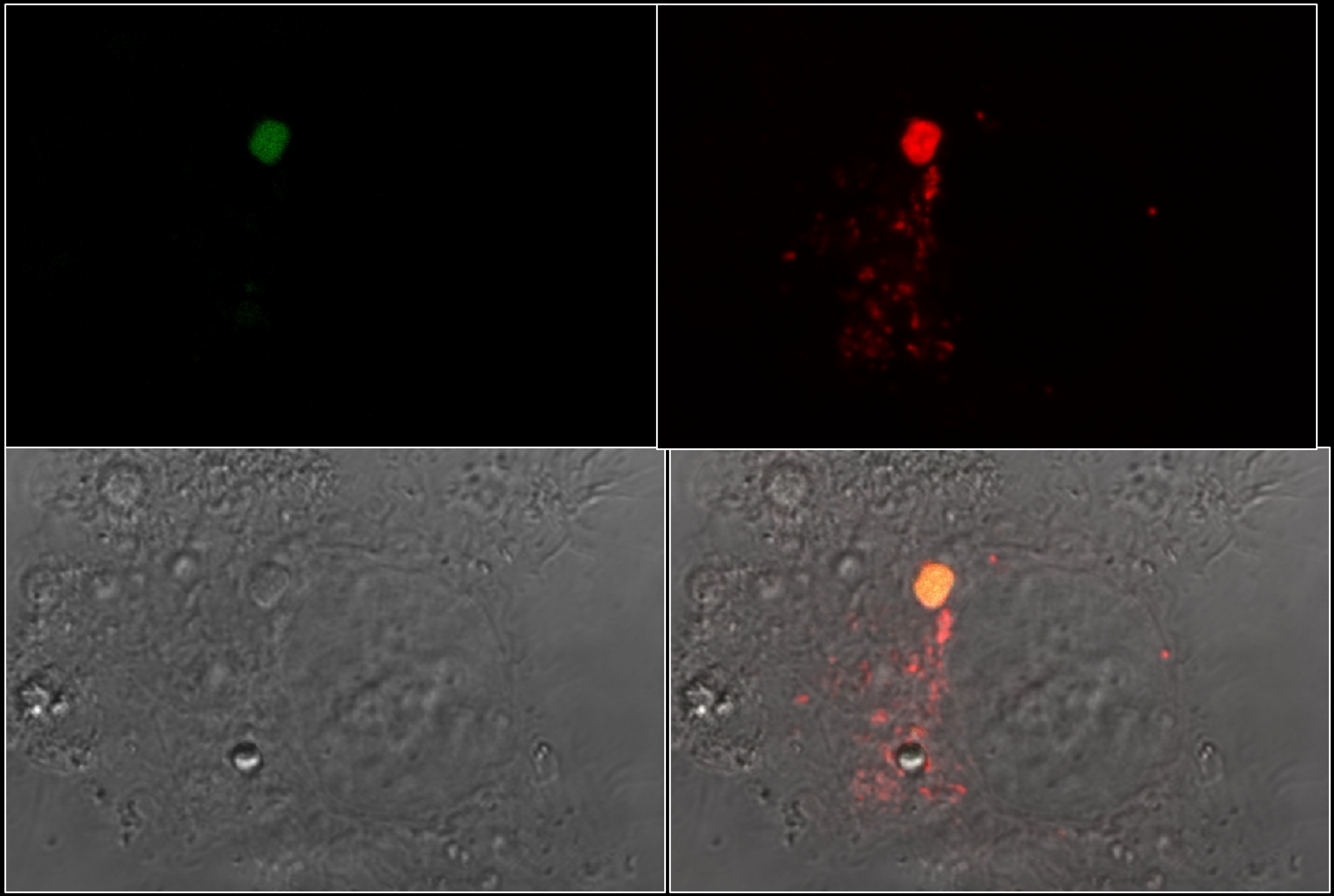

Pearsons Correlation	Colocalization Coefficient Mx	Colocalization Coefficient My	Overlap Coefficient R	Overlap Coefficient Kx	Overlap Coefficient Ky	X Min Threshold	X Max Threshold	Y Min Threshold	Y Max Threshold	Voxel Ratio Ch.X/Ch.Y	Global Pearsons Correlation
0.8735	0.6345	0.7565	0.7625	2.755	0.217	10	255	8	255	1.1605	0.7345

Colocalization Experiments HeLa

Lactose-QD (17b)

Golgi

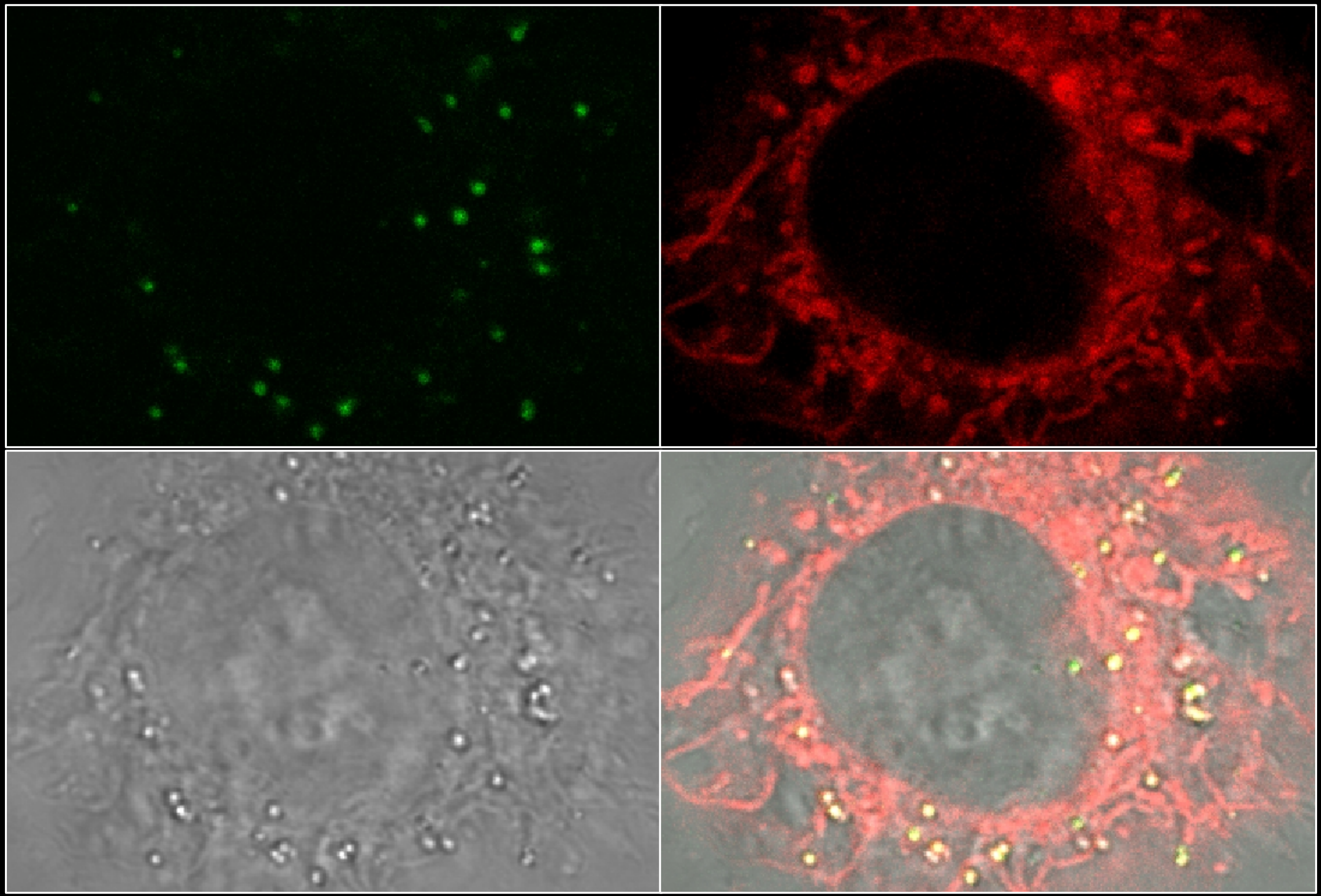

Pearsons Correlation	Colocalization Coefficient Mx	Colocalization Coefficient My	Overlap Coefficient R	Overlap Coefficient Kx	Overlap Coefficient Ky	X Min Threshold	X Max Threshold	Y Min Threshold	Y Max Threshold	Voxel Ratio Ch.X/Ch.Y	Global Pearsons Correlation
0.335	1	1	0.642	3.87	0.106	2	255	2	255	1	0.335

Colocalization Experiments HeLa

Lactose-QD (17b)

Golgi

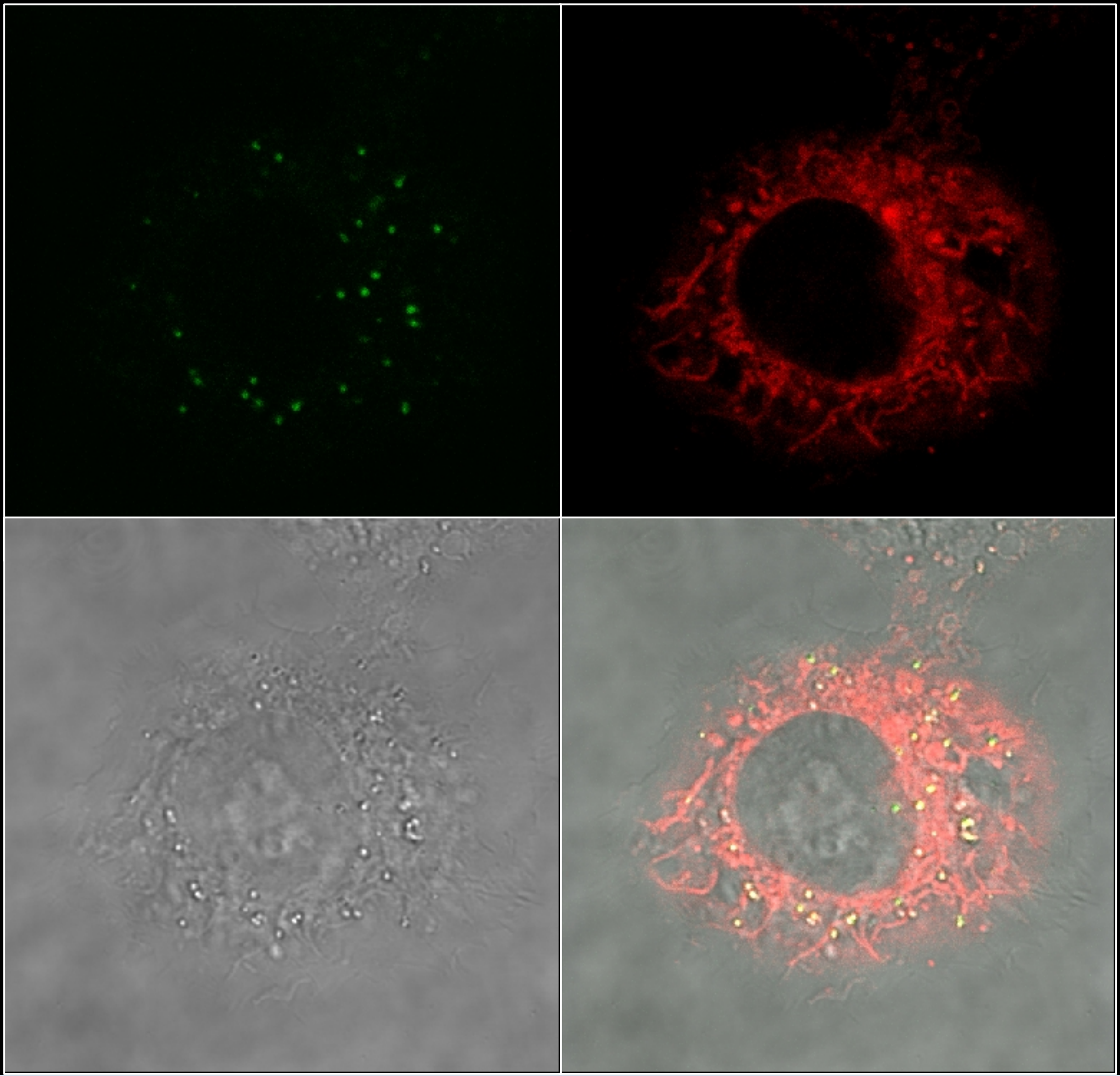

Pearsons Correlation	Colocalization Coefficient Mx	Colocalization Coefficient My	Overlap Coefficient R	Overlap Coefficient Kx	Overlap Coefficient Ky	X Min Threshold	X Max Threshold	Y Min Threshold	Y Max Threshold	Voxel Ratio Ch.X/Ch.Y	Global Pearsons Correlation
0.463	1	1	0.676	2.543	0.213	2	255	2	255	1	0.463

Colocalization Experiments AS

Lactose-QD (17b)

Early Endosomes

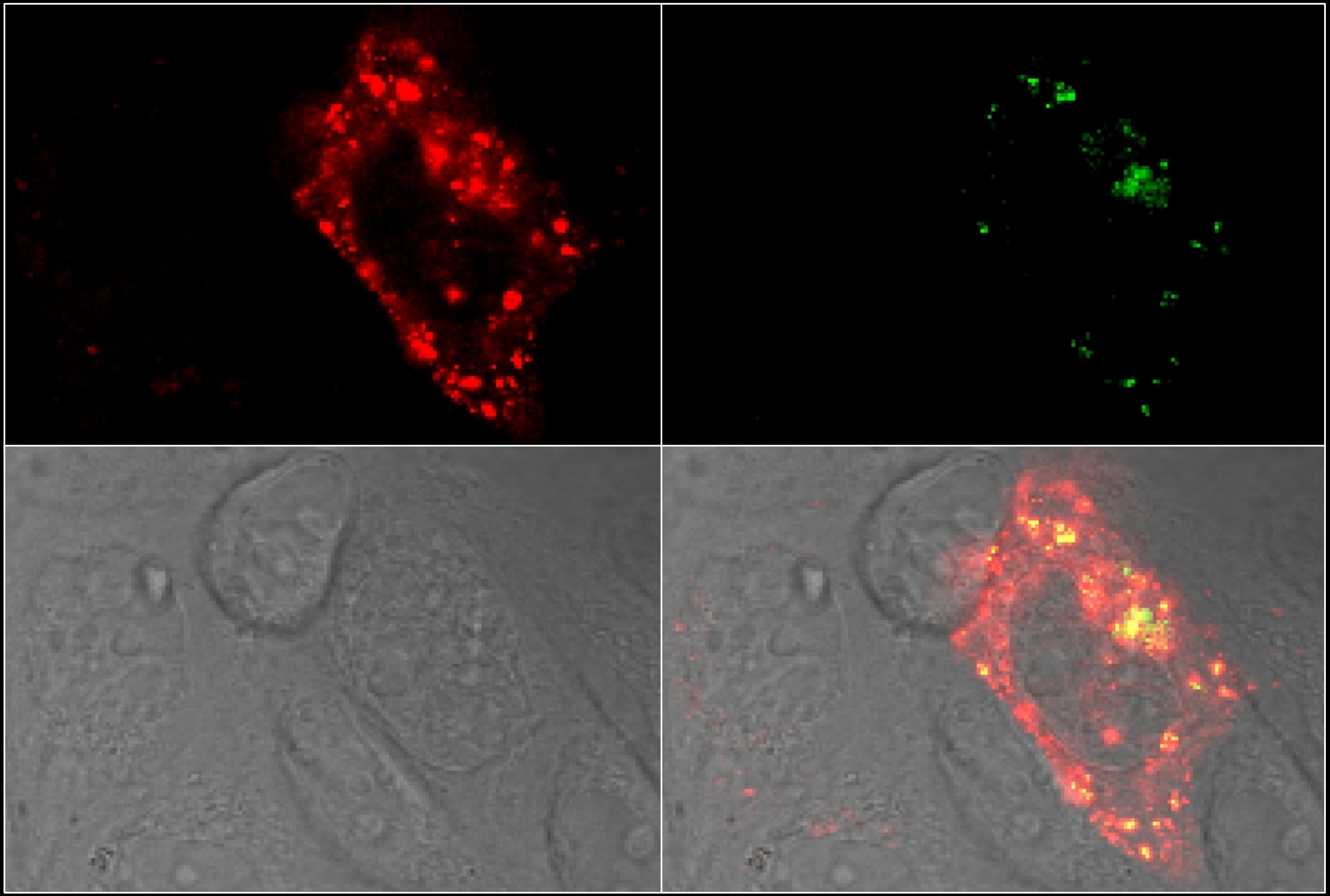

S58

Pearsons Correlation	Colocalization Coefficient	Colocalization Coefficient	Overlap Coefficient R	Overlap Coefficient Kx	Overlap Coefficient Ky	X Min Threshold	X Max Threshold	Y Min Threshold	Y Max Threshold	Voxel Ratio Ch.X/Ch.Y	Global Pearsons
0.6575	0.834	0.649	0.6165	0.5335	0.739	19	251	11	251	509.8075	0.5095

Colocalization Experiments AS

Lactose-QD (17b)

Late Endosomes

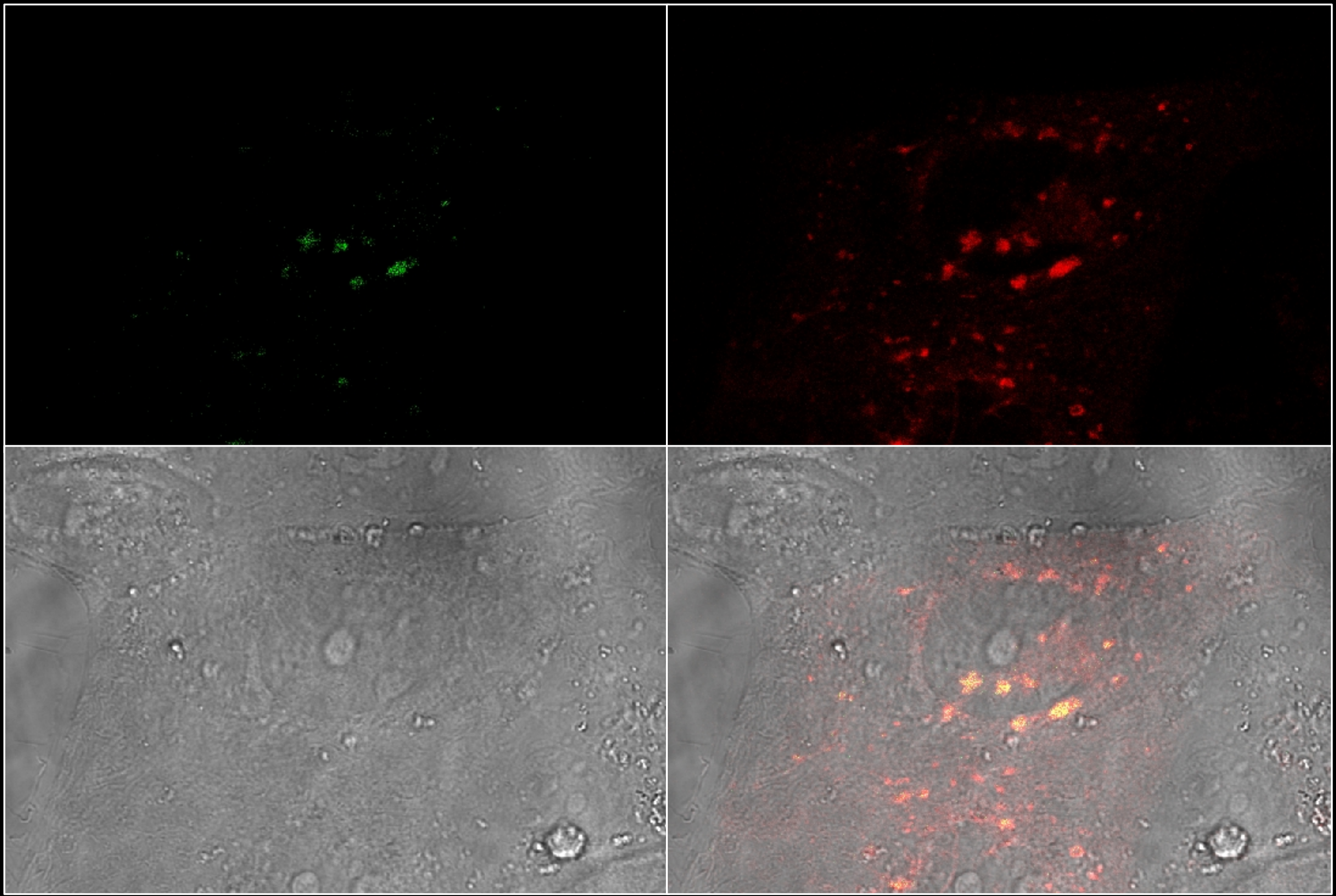

Pearsons Correlation	Colocalization Coefficient Mx	Colocalization Coefficient My	Overlap Coefficient R	Overlap Coefficient Kx	Overlap Coefficient Ky	X Min Threshold	X Max Threshold	Y Min Threshold	Y Max Threshold	Voxel Ratio Ch.X/Ch.Y	Global Pearsons Correlation
0.5165	1	1	0.5965	1.955	0.1815	0	255	0	255	1	0.5165

Colocalization Experiments AS

Lactose-QD (17b)

Lysosomes

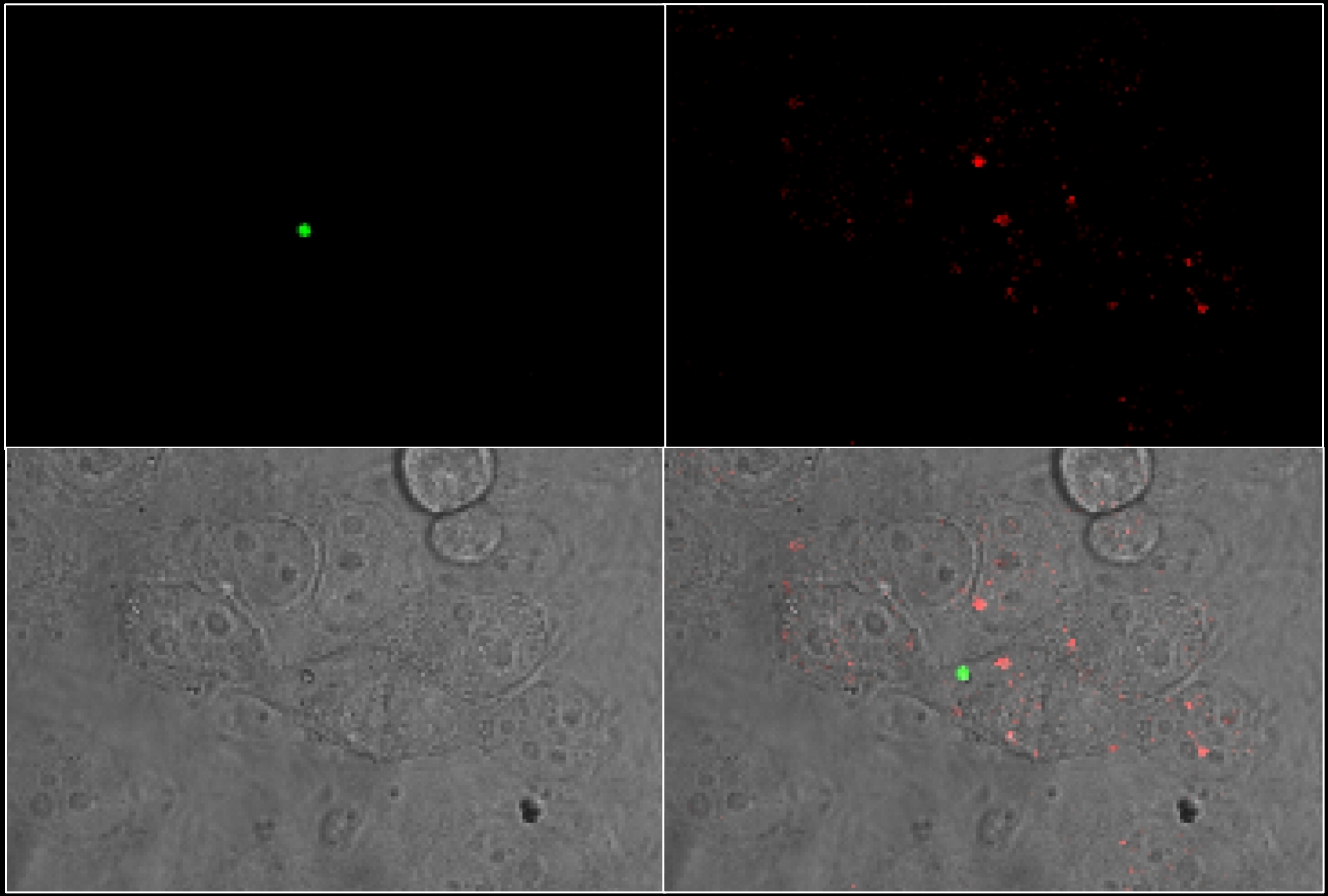

Pearsons Correlation	Colocalization Coefficient Mx	Colocalization Coefficient My	Overlap Coefficient R	Overlap Coefficient Kx	Overlap Coefficient Ky	X Min Threshold	X Max Threshold	Y Min Threshold	Y Max Threshold	Voxel Ratio Ch.X/Ch.Y	Global Pearsons Correlation
0.2225	0.0221	0.653	0.06685	0.01685	0.2665	13	255	5	255	17.63	0.03725

Colocalization Experiments AS

Lactose-QD (17b)

Lysosomes

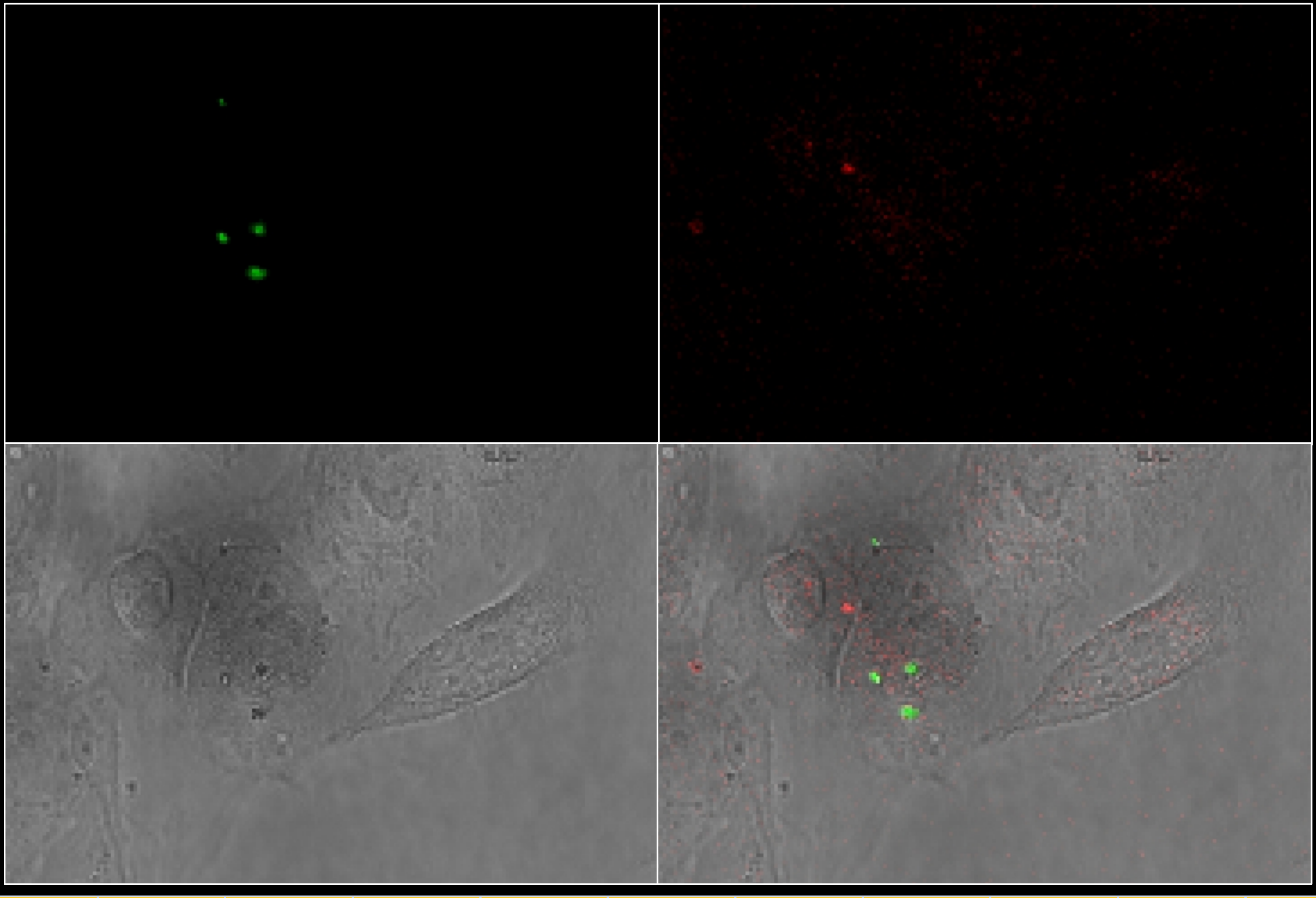

Pearsons Correlation	Colocalization Coefficient Mx	Colocalization Coefficient My	Overlap Coefficient R	Overlap Coefficient Kx	Overlap Coefficient Ky	X Min Threshold	X Max Threshold	Y Min Threshold	Y Max Threshold	Voxel Ratio Ch.X/Ch.Y	Global Pearsons Correlation
0.0417	0.04895	0.683	0.087	0.0445	0.4515	12	255	5	255	13.86	0.11405

Colocalization Experiments AS

Lactose-QD (17b)

Nucleus

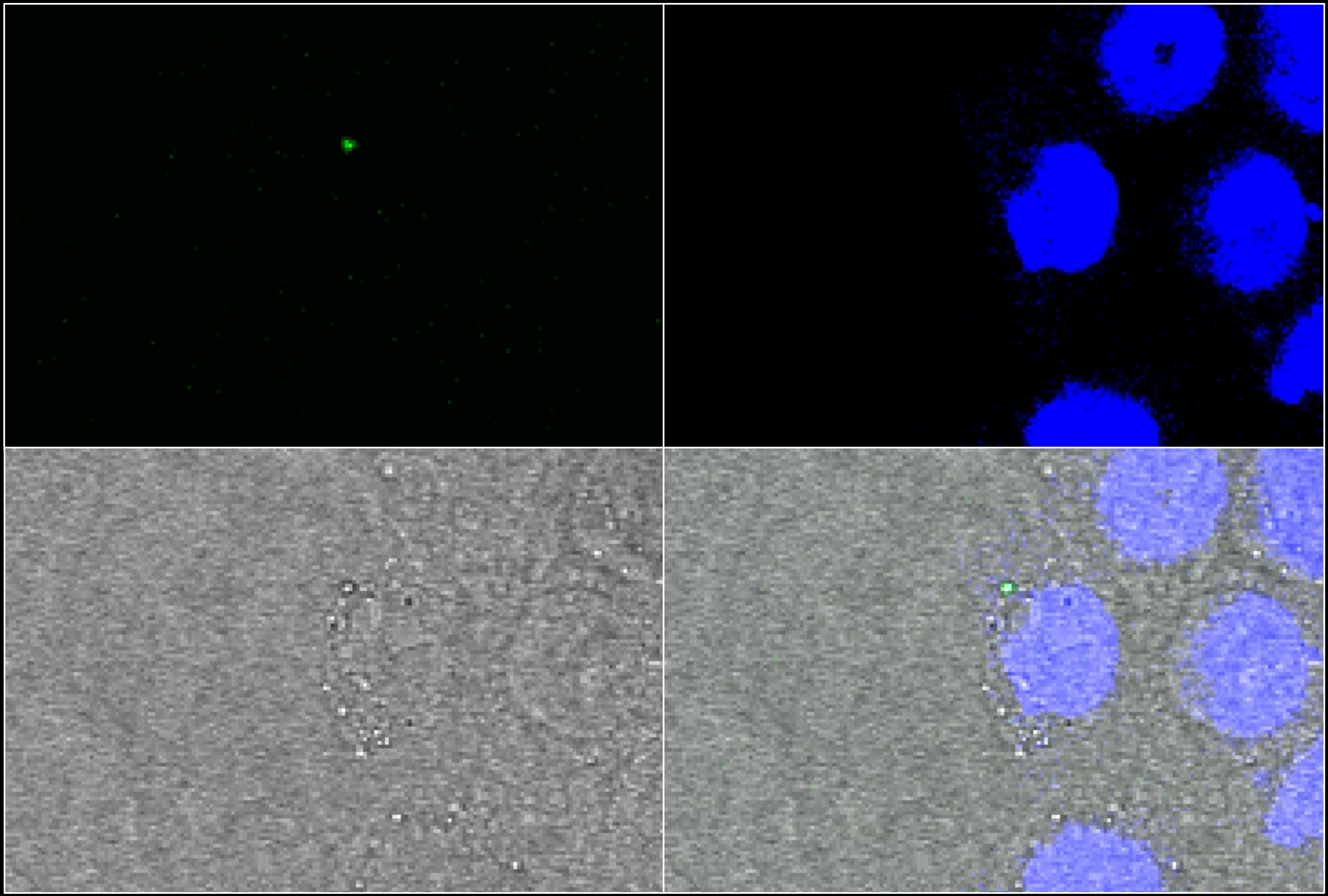

Colocalization Experiments AS

Lactose-QD (17b)

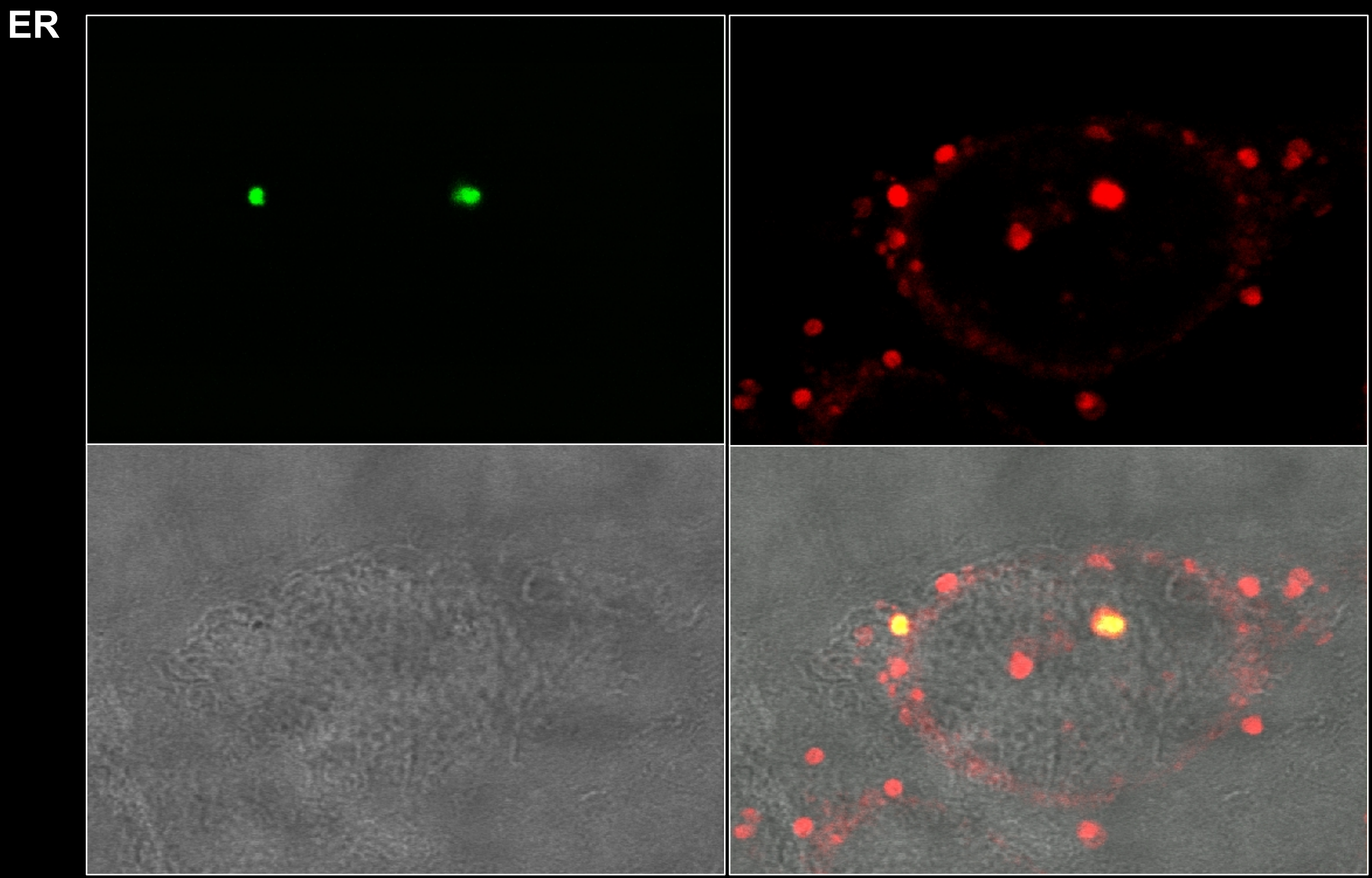

Pearsons Correlation	Colocalization Coefficient Mx	Colocalization Coefficient My	Overlap Coefficient R	Overlap Coefficient Kx	Overlap Coefficient Ky	X Min Threshold	X Max Threshold	Y Min Threshold	Y Max Threshold	Voxel Ratio Ch.X/Ch.Y	Global Pearsons Correlation
0.56	0.787	1	0.763	1.248	0.467	12	255	15	255	1.64	0.543

Colocalization Experiments AS

Lactose-QD (17b)

Golgi

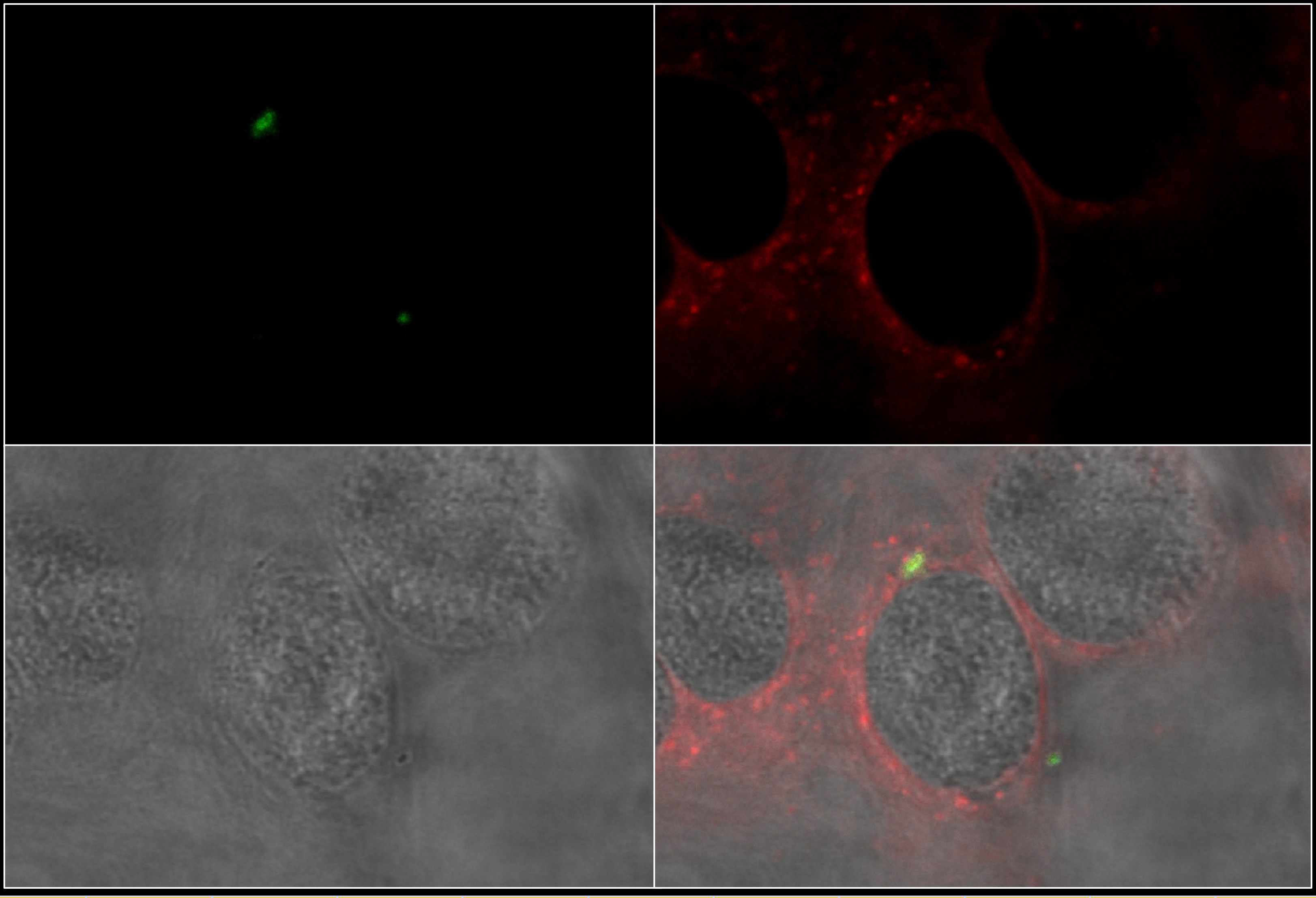

Pearsons Correlation	Colocalization Coefficient Mx	Colocalization Coefficient My	Overlap Coefficient R	Overlap Coefficient Kx	Overlap Coefficient Ky	X Min Threshold	X Max Threshold	Y Min Threshold	Y Max Threshold	Voxel Ratio Ch.X/Ch.Y	Global Pearsons Correlation
0.176	0.711	1	0.7055	1.555	0.3215	5	173	8	173	1.475	0.2155

Colocalization Experiments AS

Lactose/Mann-QD (20b)

Early Endosomes

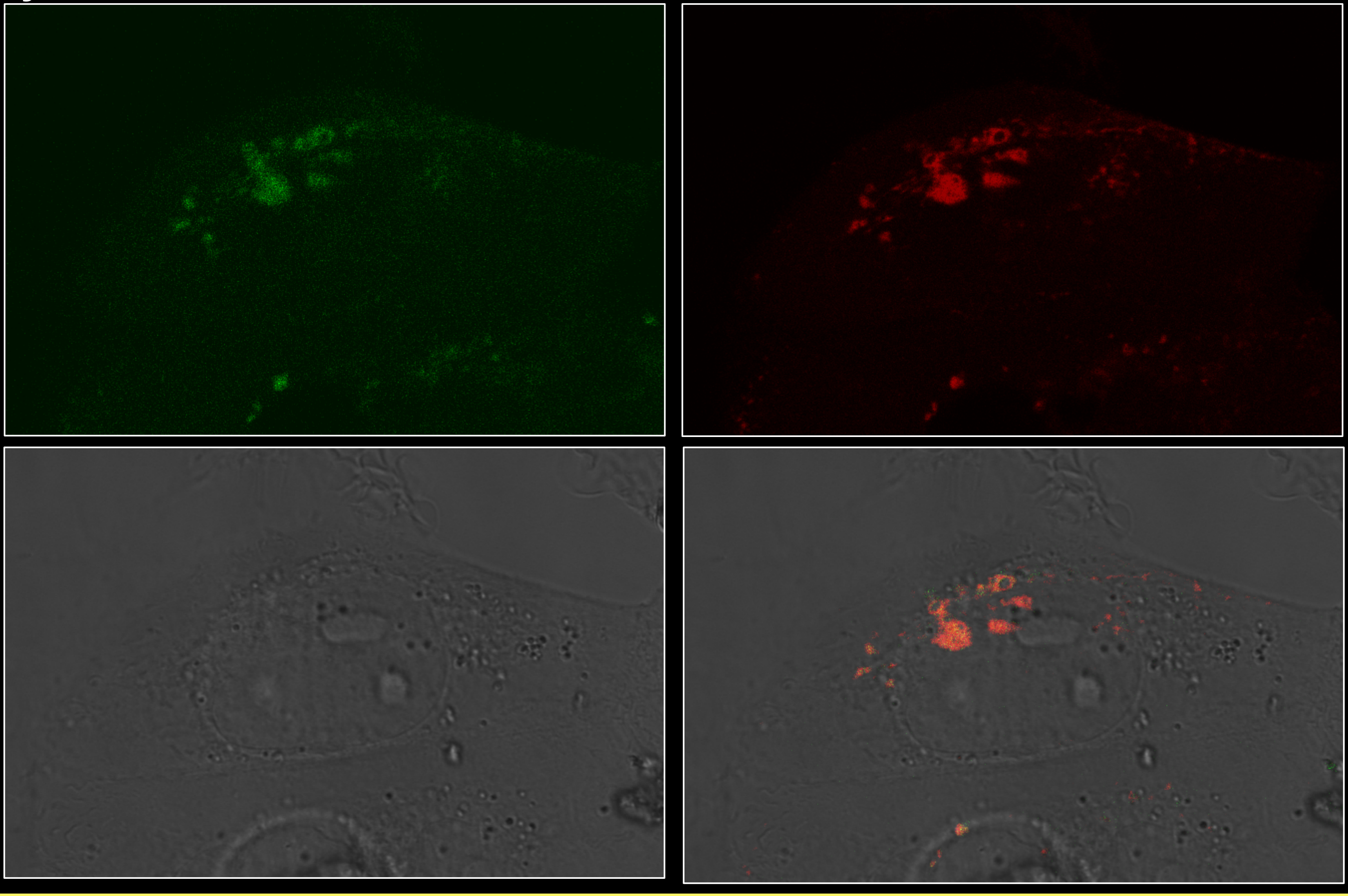

Pearsons Correlation	Colocalization Coefficient M1	Colocalization Coefficient M2	Overlap Coefficient R	Overlap Coefficient k1	Overlap Coefficient k2	Ch.1 Threshold	Ch.2 Threshold	Voxel Ratio Ch.1 / Ch.2	Global Pearsons Correlation
0.63	0.649	0.532	0.655	1.54	0.279	9	10	0.631	0.708

Colocalization Experiments AS

Lactose/Mann-QD (20b)

Late Endosomes

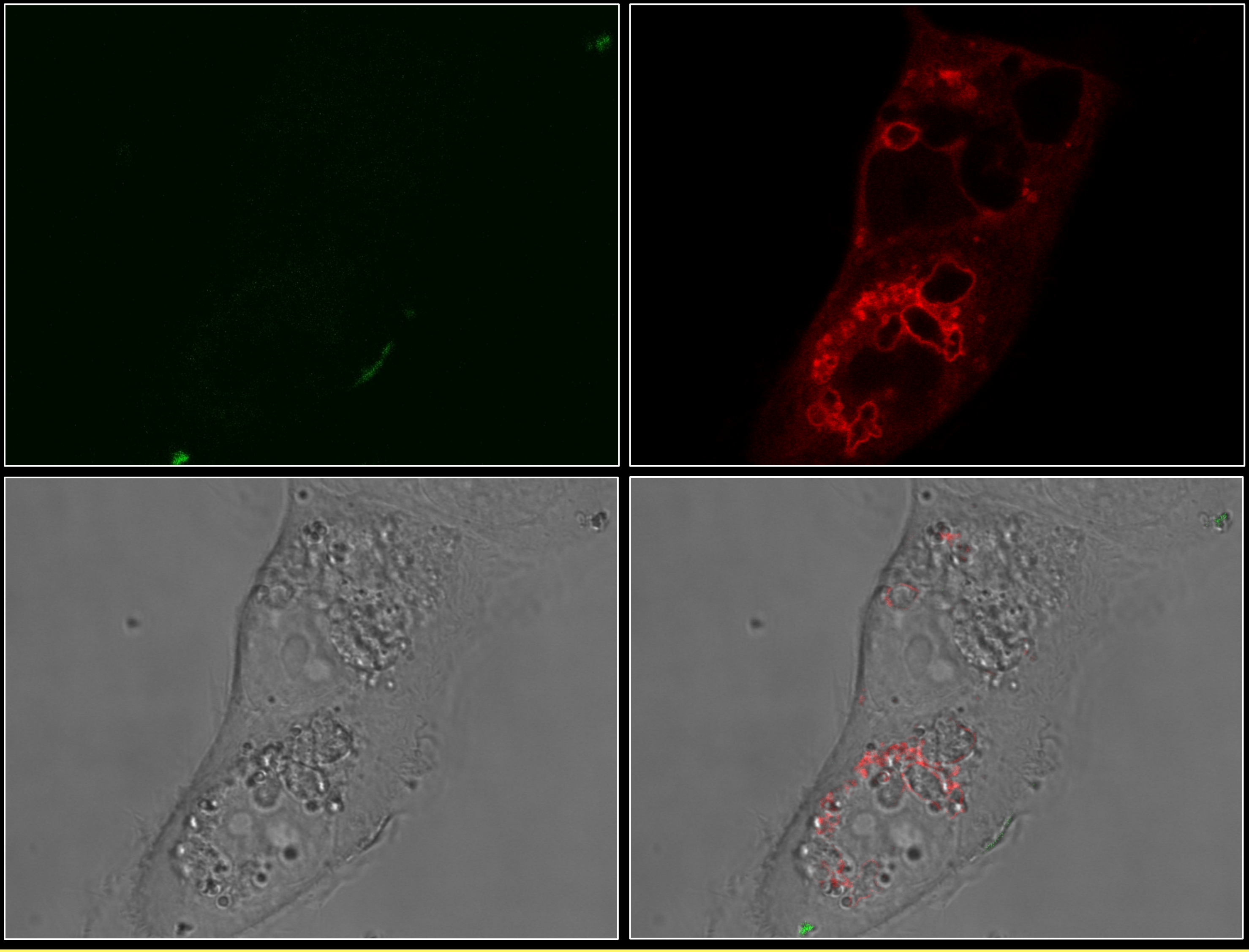

Pearsons Correlation	Colocalization Coefficient M1	Colocalization Coefficient M2	Overlap Coefficient R	Overlap Coefficient k1	Overlap Coefficient k2	Ch.1 Threshold	Ch.2 Threshold	Voxel Ratio Ch.1 / Ch.2	Global Pearsons Correlation
-0.074	0.209	0.047	0.074	0.351	0.019	9.333	11.000	0.169	0.068

Colocalization Experiments AS

Lactose/Mann-QD (20b)

ER

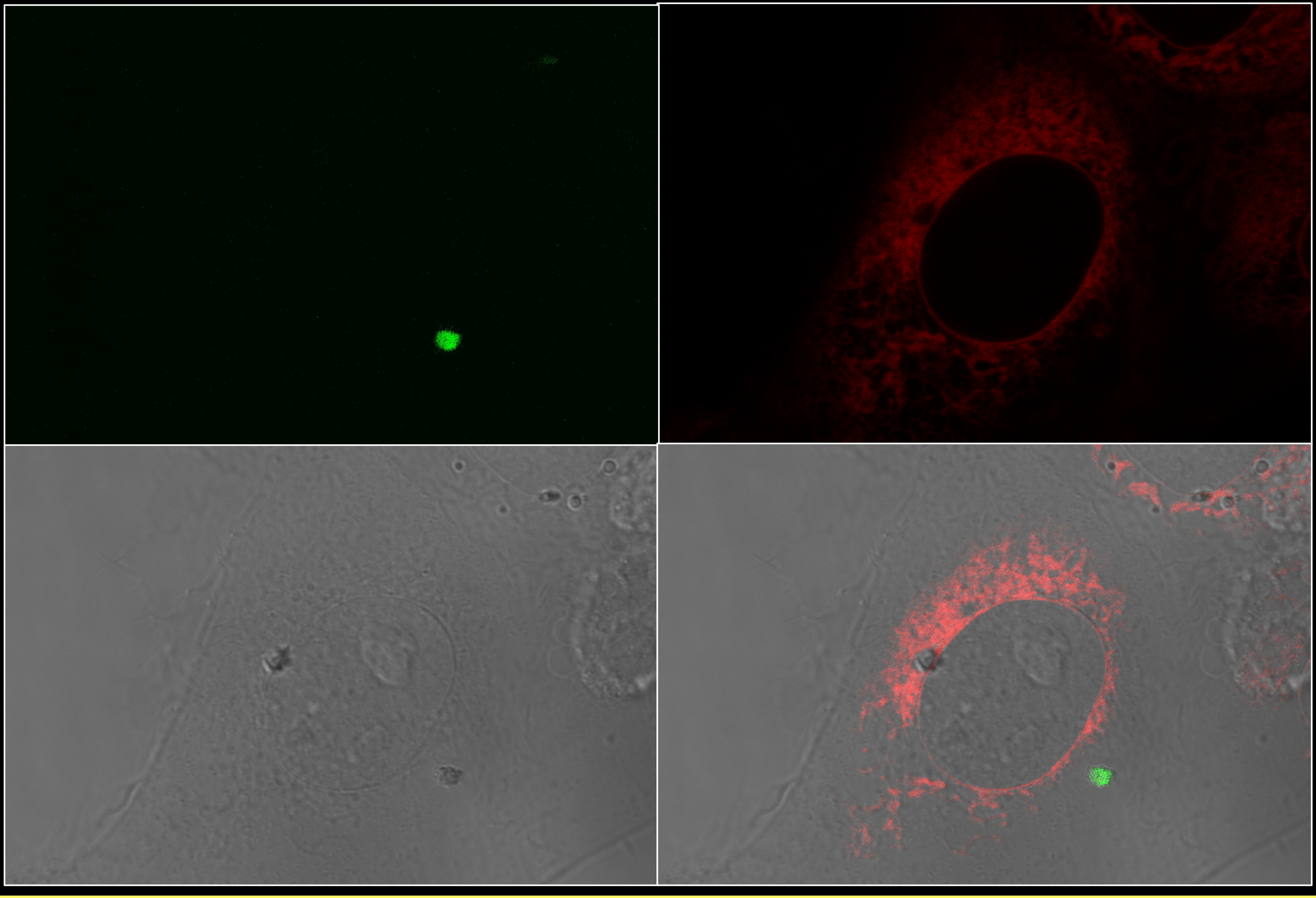

Pearsons Correlation	Colocalization Coefficient M1	Colocalization Coefficient M2	Overlap Coefficient R	Overlap Coefficient k1	Overlap Coefficient k2	Ch.1 Threshold	Ch.2 Threshold	Voxel Ratio Ch.1 / Ch.2	Global Pearsons Correlation
-0.17152	0.15422	0.007432	0.02652	0.4868	0.0016668	11.6	39	0.04928	0.02222

Colocalization Experiments AS

Lactose/Mann-QD (20b)

Lyso

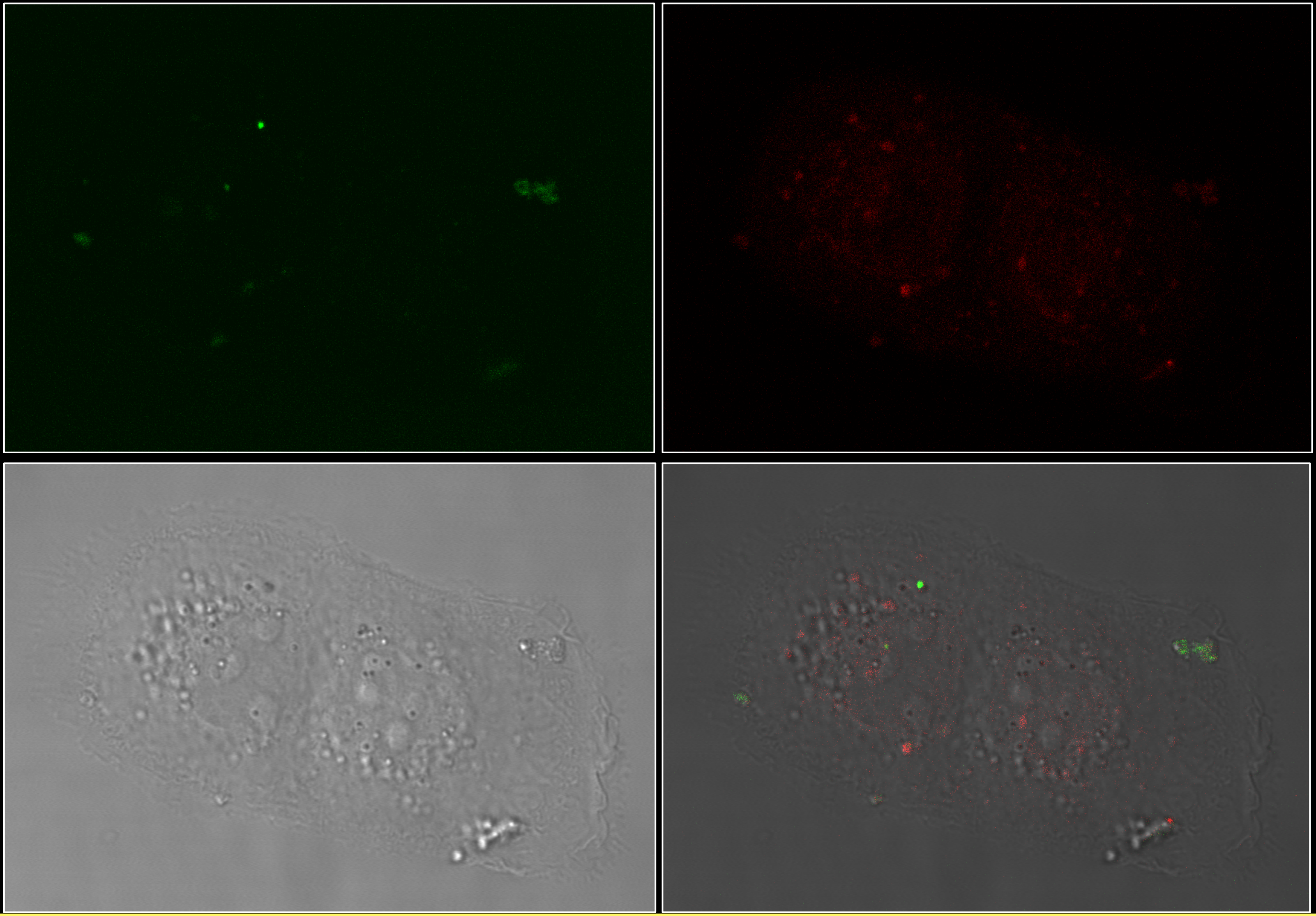

Pearsons Correlation	Colocalization Coefficient M1	Colocalization Coefficient M2	Overlap Coefficient R	Overlap Coefficient k1	Overlap Coefficient k2	Ch.1 Threshold	Ch.2 Threshold	Voxel Ratio Ch.1 / Ch.2	Global Pearsons Correlation
0.003	0.224	0.223	0.136	0.155	0.154	7.667	9.833	3.537	0.081

Colocalization Experiments AS

Lactose/Mann-QD (20b)

Golgi

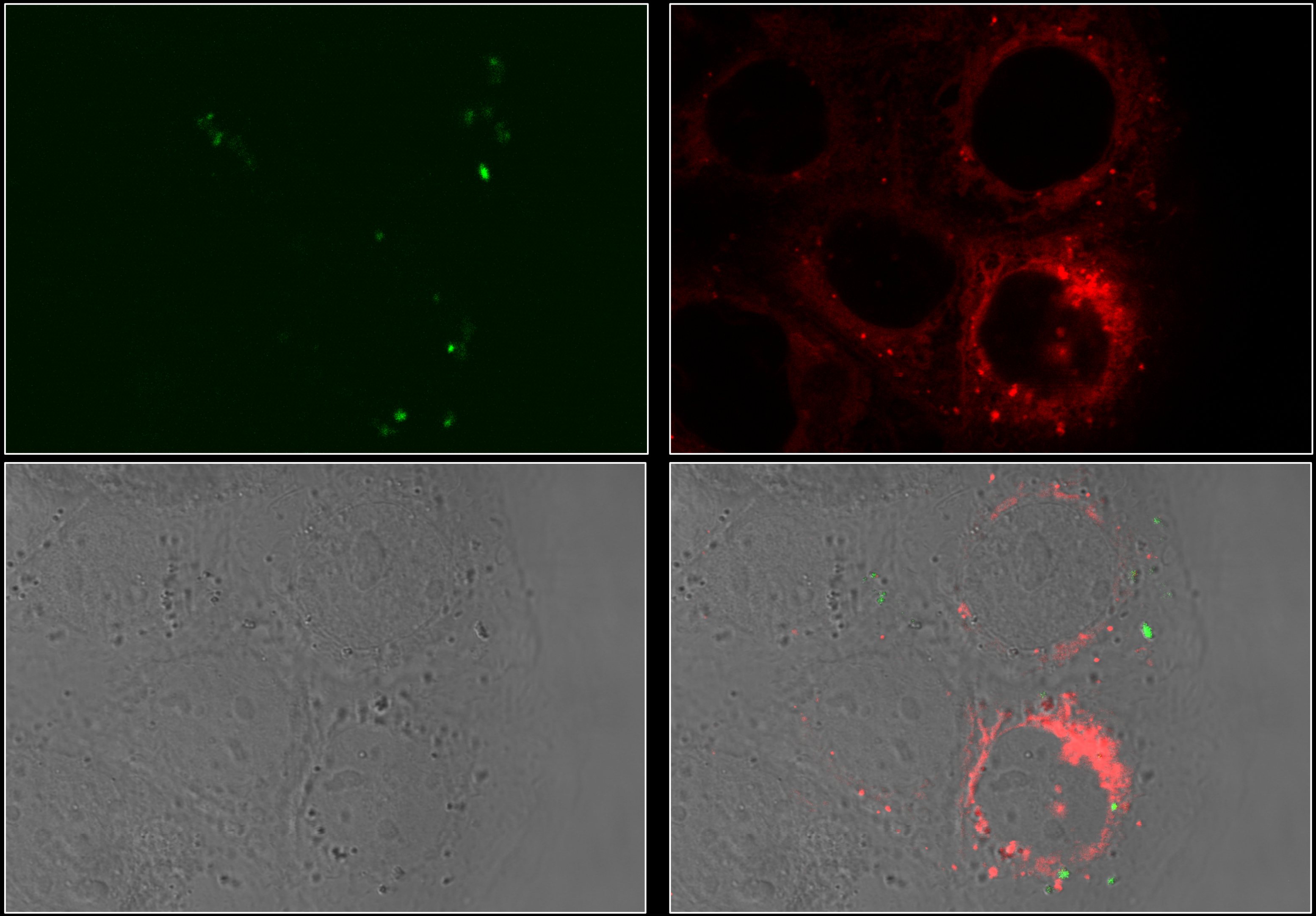

Pearsons Correlation	Colocalization Coefficient M1	Colocalization Coefficient M2	Overlap Coefficient R	Overlap Coefficient k1	Overlap Coefficient k2	Ch.1 Threshold	Ch.2 Threshold	Voxel Ratio Ch.1 / Ch.2	Global Pearsons Correlation
0.085	0.266	0.064	0.121	0.554	0.041	10.167	19.000	0.241	0.119

Colocalization Experiments HeLa

Lactose/Mann-QD (20b)

Early Endosomes

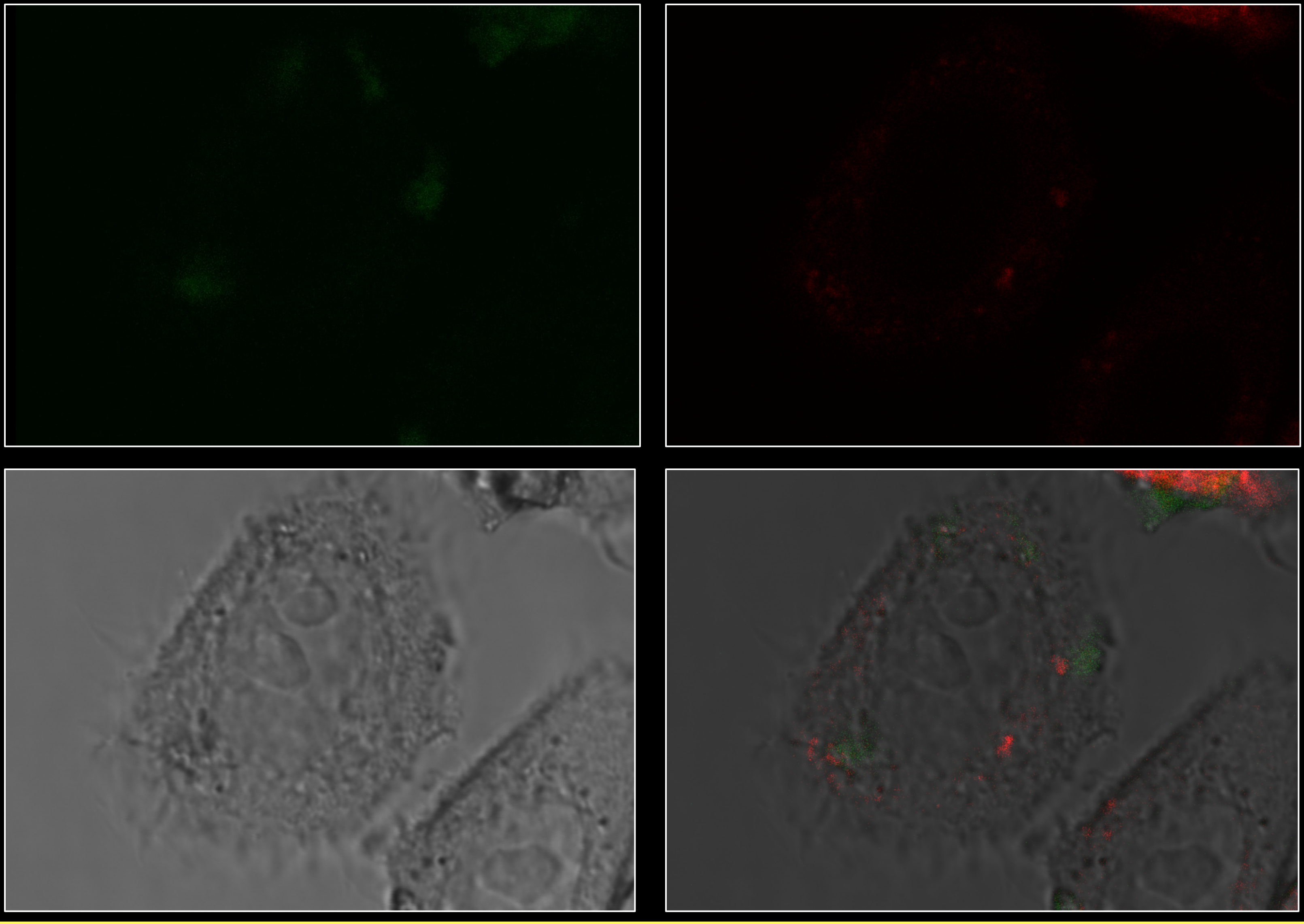

Pearsons Correlation	Colocalization Coefficient M1	Colocalization Coefficient M2	Overlap Coefficient R	Overlap Coefficient k1	Overlap Coefficient k2	Ch.1 Threshold	Ch.2 Threshold	Voxel Ratio Ch.1 / Ch.2	Global Pearsons Correlation
0.410	0.589	0.666	0.578	3.190	0.106	4.500	12.000	0.894	0.565

Colocalization Experiments HeLa

Lactose/Mann-QD (20b)

Late Endosomes

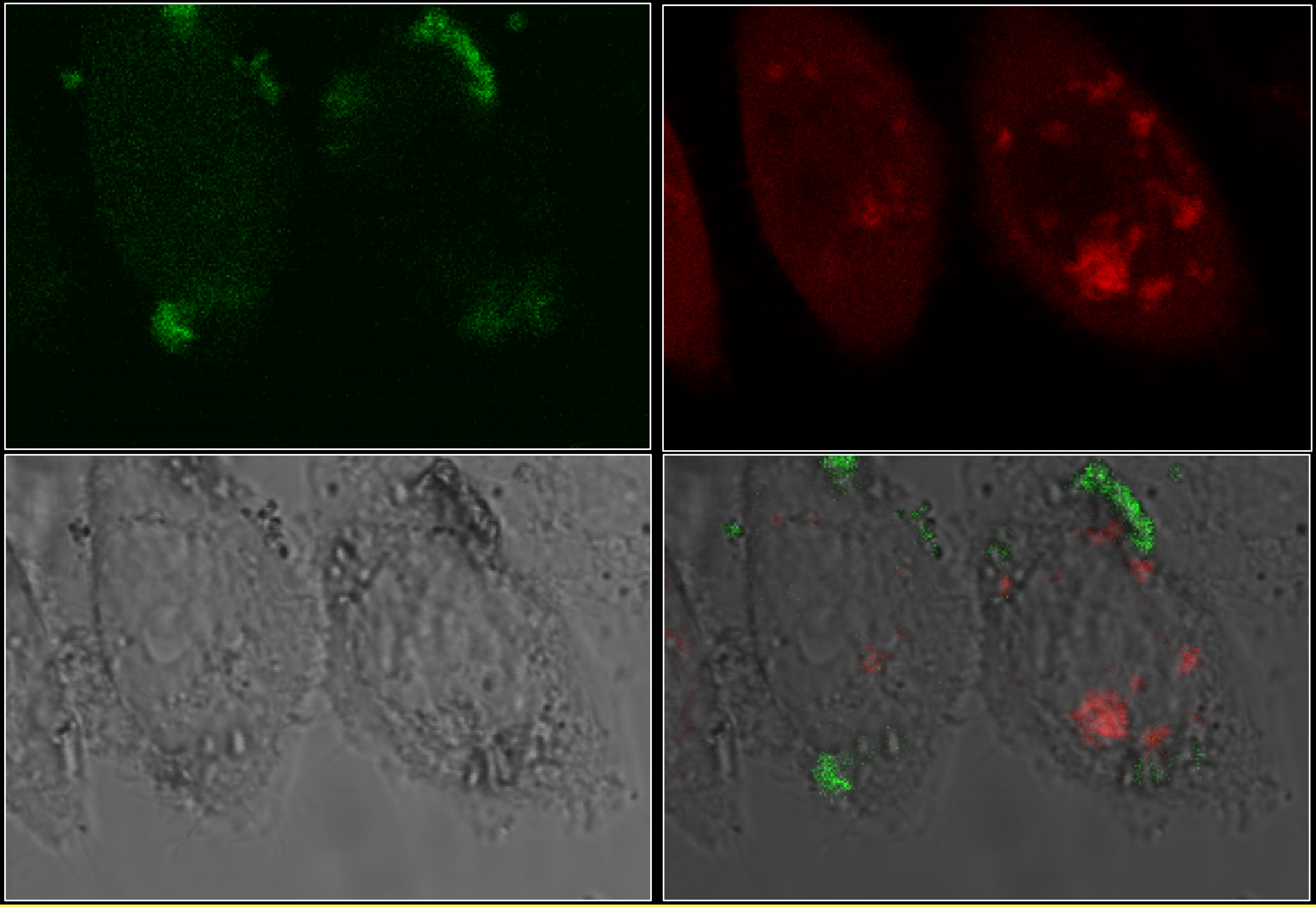

Pearsons Correlation	Colocalization Coefficient M1	Colocalization Coefficient M2	Overlap Coefficient R	Overlap Coefficient k1	Overlap Coefficient k2	Ch.1 Threshold	Ch.2 Threshold	Voxel Ratio Ch.1 / Ch.2	Global Pearsons Correlation
-0.082	0.436	0.448	0.304	0.731	0.792	8.000	10.750	1.058	0.250

Colocalization Experiments HeLa

Lactose/Mann-QD (20b)

ER

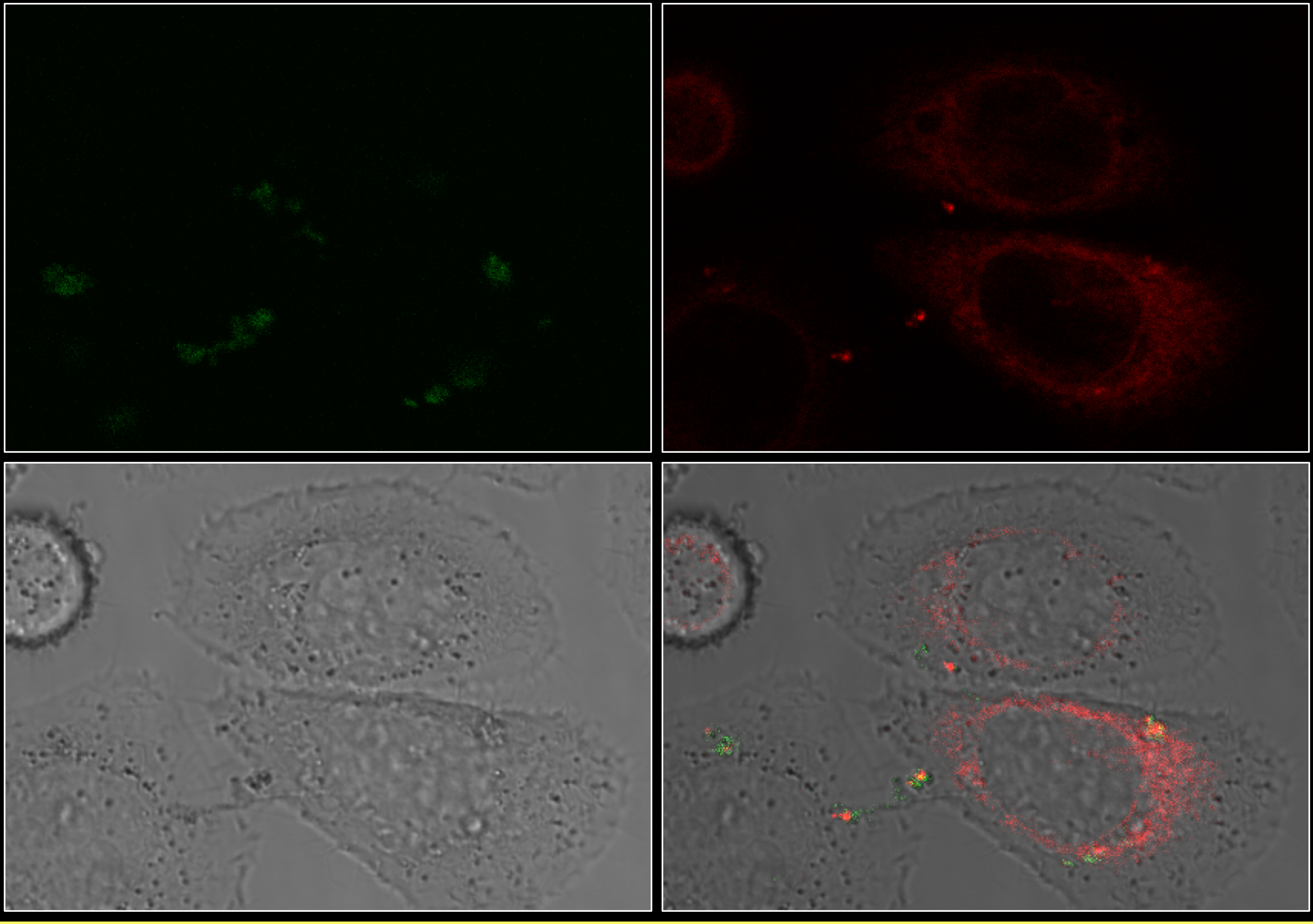

Pearsons Correlation	Colocalization Coefficient M1	Colocalization Coefficient M2	Overlap Coefficient R	Overlap Coefficient k1	Overlap Coefficient k2	Ch.1 Threshold	Ch.2 Threshold	Voxel Ratio Ch.1 / Ch.2	Global Pearsons Correlation
0.227	0.253	0.070	0.158	1.005	0.028	6.500	24.000	0.270	0.156

Colocalization Experiments HeLa

Lactose/Mann-QD (20b)

Lyso

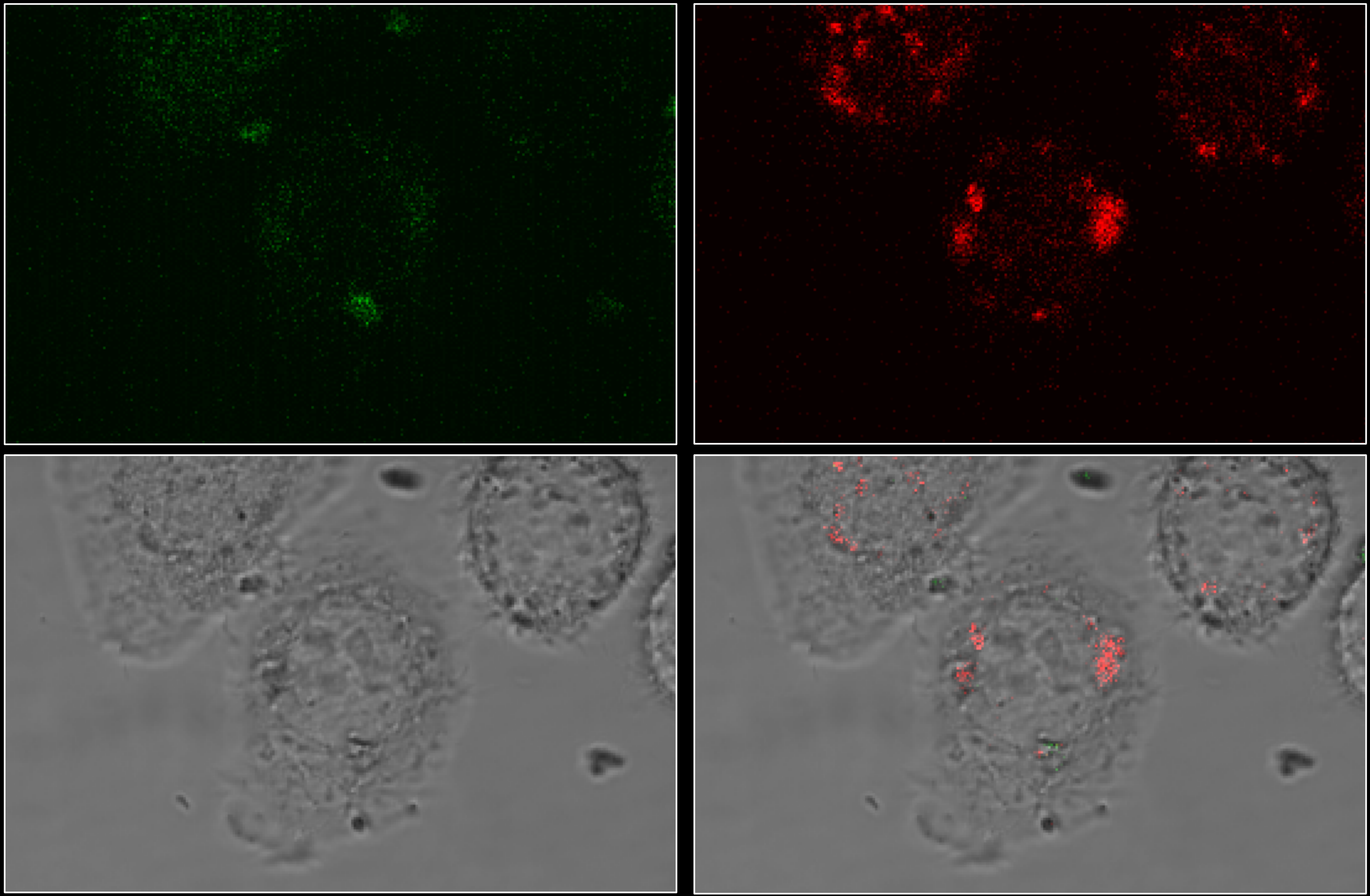

Pearsons Correlation	Colocalization Coefficient M1	Colocalization Coefficient M2	Overlap Coefficient R	Overlap Coefficient k1	Overlap Coefficient k2	Ch.1 Threshold	Ch.2 Threshold	Voxel Ratio Ch.1 / Ch.2	Global Pearsons Correlation
0.167	0.449	0.231	0.330	0.244	0.467	10.000	4.000	0.449	0.325

Colocalization Experiments HeLa

Lactose/Mann-QD (20b)

Golgi

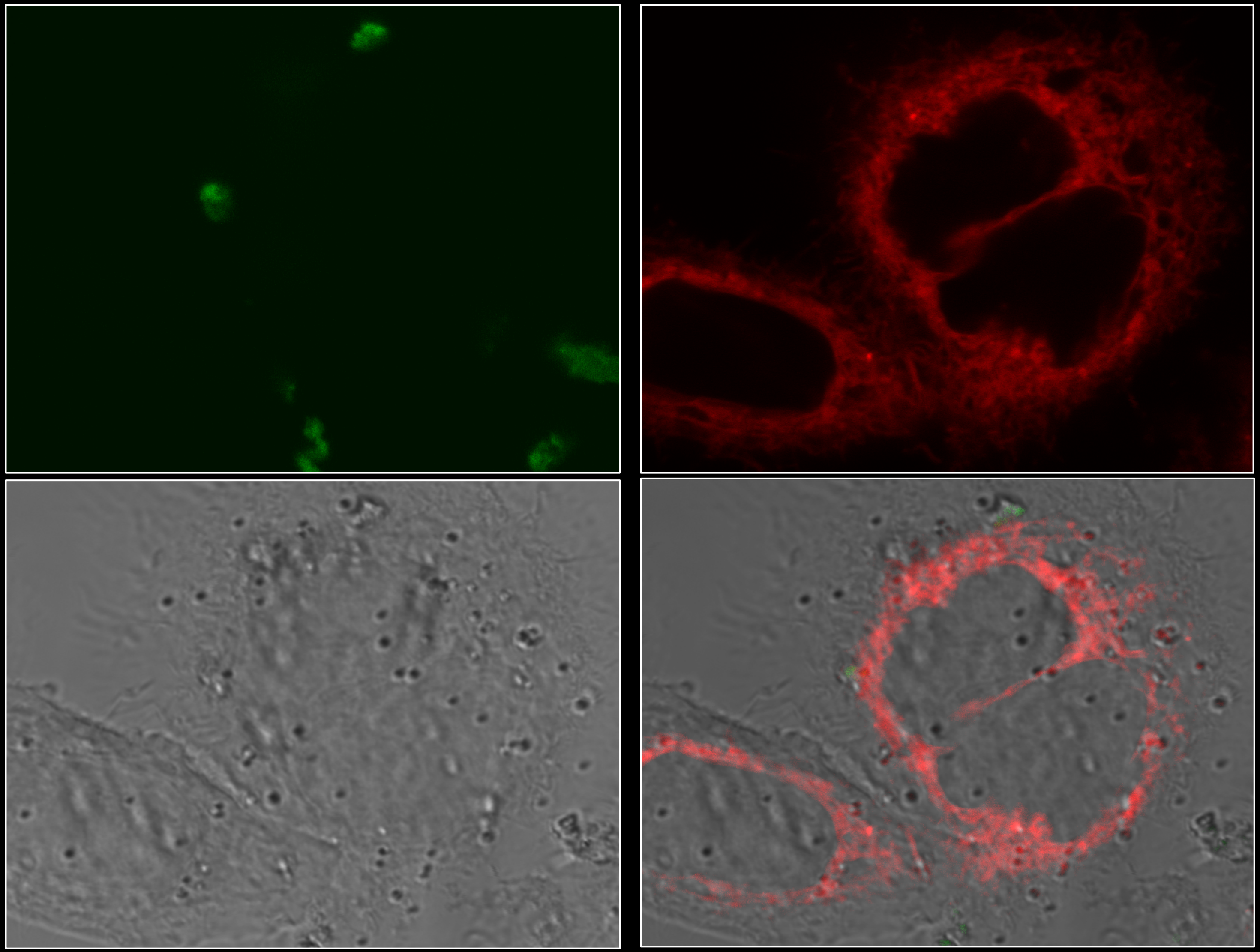

Pearsons Correlation	Colocalization Coefficient M1	Colocalization Coefficient M2	Overlap Coefficient R	Overlap Coefficient k1	Overlap Coefficient k2	Ch.1 Threshold	Ch.2 Threshold	Voxel Ratio Ch.1 / Ch.2	Global Pearsons Correlation
0.031	0.785	0.760	0.447	2.390	0.146	4.000	7.500	0.921	0.107

Colocalization Experiments HeLa

Lact/Malt3-QD (21b)

Early Endosomes

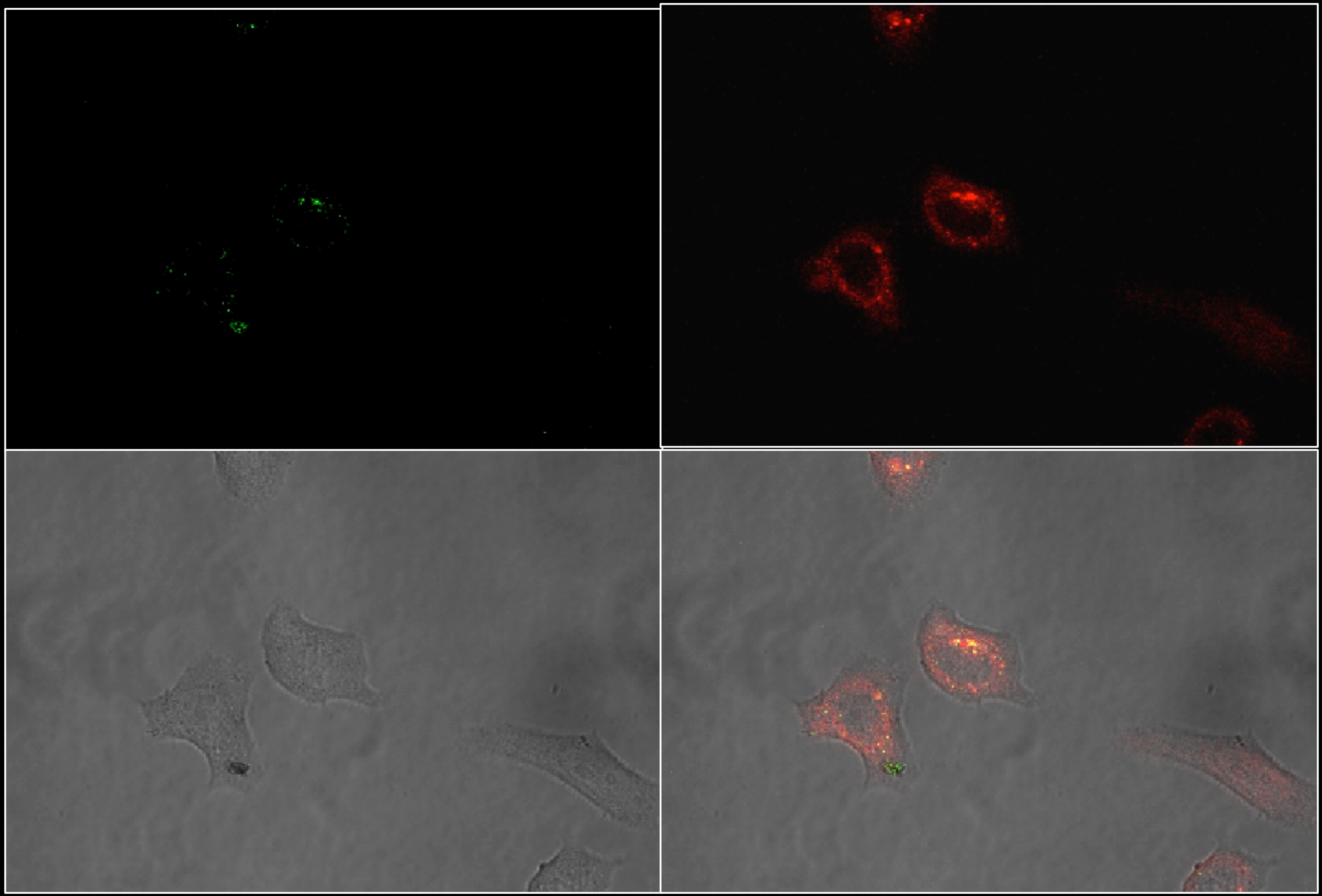

Pearsons Correlation	Colocalization Coefficient Mx	Colocalization Coefficient My	Overlap Coefficient R	Overlap Coefficient Kx	Overlap Coefficient Ky	X Min Threshold	X Max Threshold	Y Min Threshold	Y Max Threshold	Voxel Ratio Ch.X/Ch.Y	Global Pearsons Correlation
0.675	0.644	0.9994	0.698	1.53	0.319	2	255	2	255	3.167	0.661

Colocalization Experiments HeLa

Lact/Malt3-QD (21b)

Late Endosomes

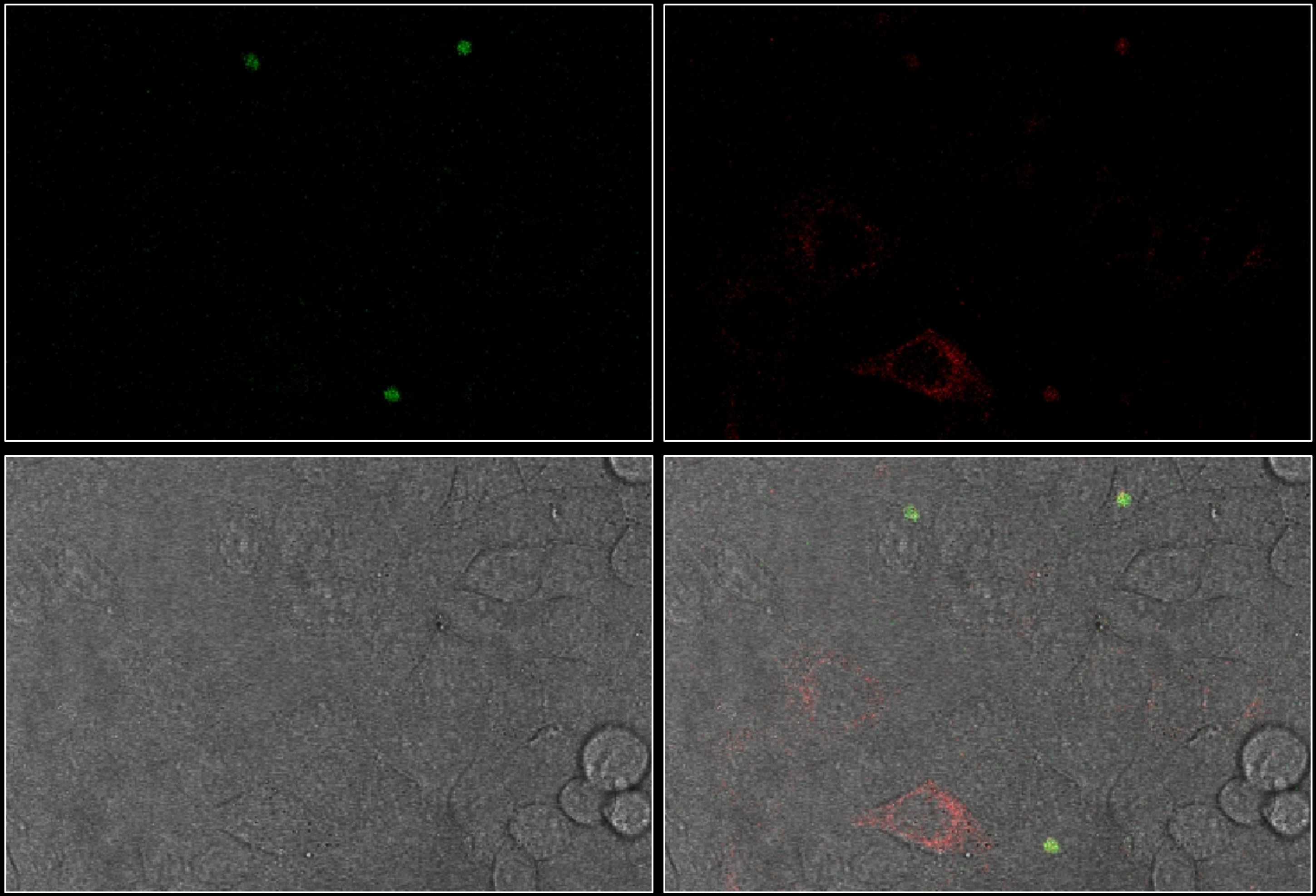

Pearsons Correlation	Colocalization Coefficient Mx	Colocalization Coefficient My	Overlap Coefficient R	Overlap Coefficient Kx	Overlap Coefficient Ky	X Min Threshold	X Max Threshold	Y Min Threshold	Y Max Threshold	Voxel Ratio Ch.X/Ch.Y	Global Pearsons Correlation
0.427	1	1	0.647	0.93	0.864	3	255	3	255	1	0.427

Colocalization Experiments HeLa

Lact/Malt3-QD (21b)

Nucleus

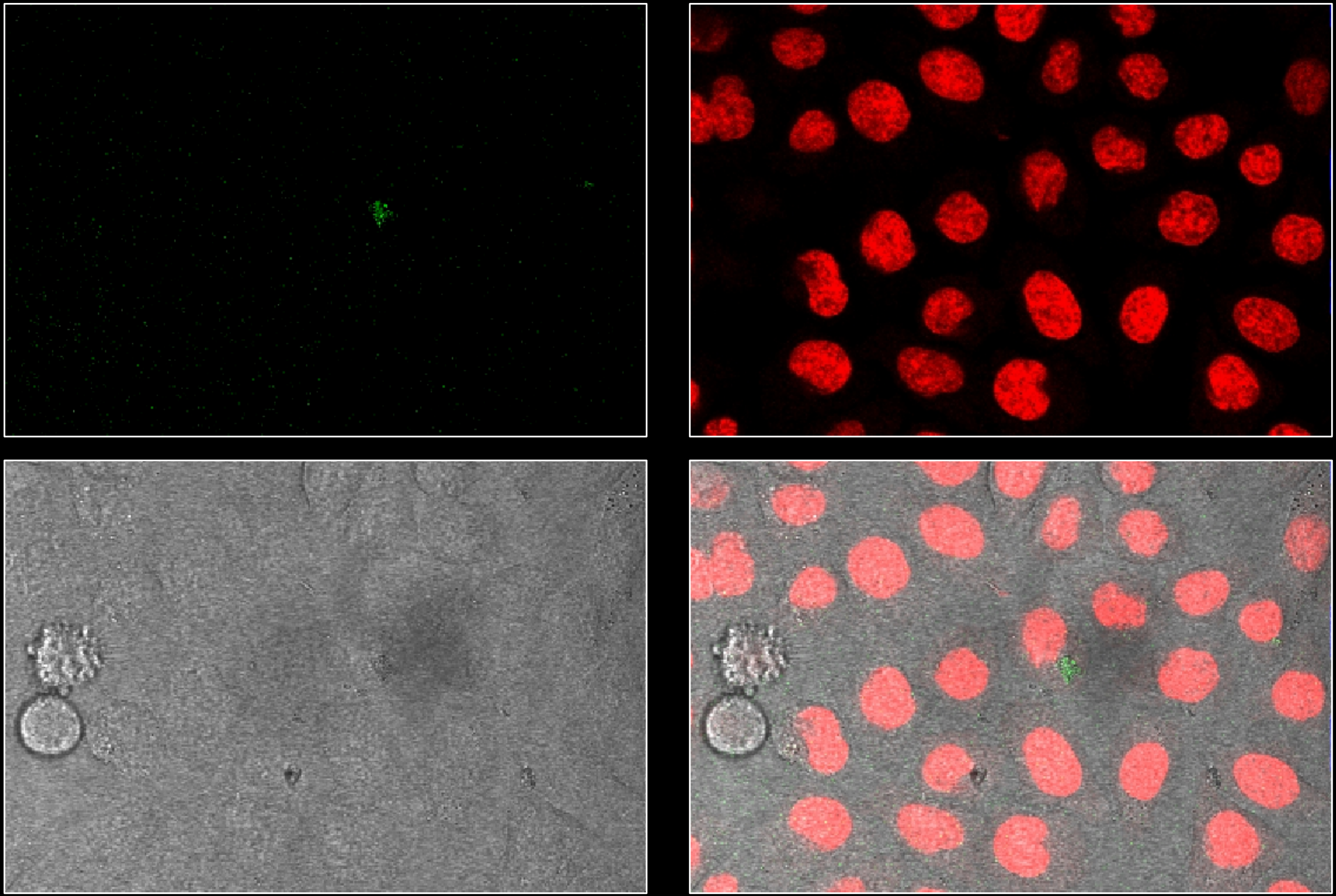

Colocalization Experiments HeLa

Lact/Malt3-QD (21b)

Lysosomes

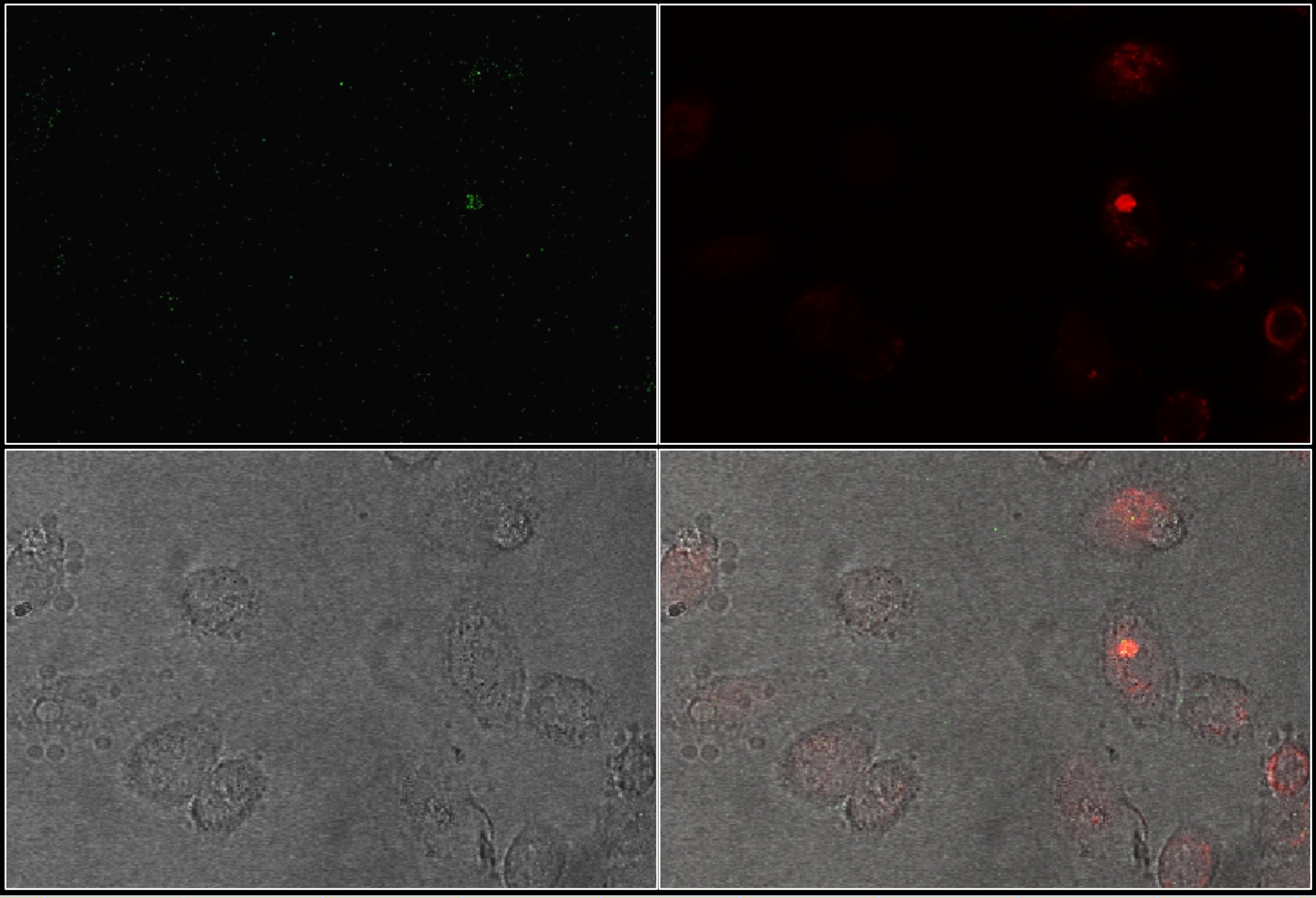

Pearsons Correlation	Colocalization Coefficient Mx	Colocalization Coefficient My	Overlap Coefficient R	Overlap Coefficient Kx	Overlap Coefficient Ky	X Min Threshold	X Max Threshold	Y Min Threshold	Y Max Threshold	Voxel Ratio Ch.X/Ch.Y	Global Pearsons Correlation
0.2695	0.2435	0.6635	0.407	0.07955	2.3155	6	255	5	255	7.575	0.3755

Colocalization Experiments HeLa

Lact/Malt3-QD (21b)

ER

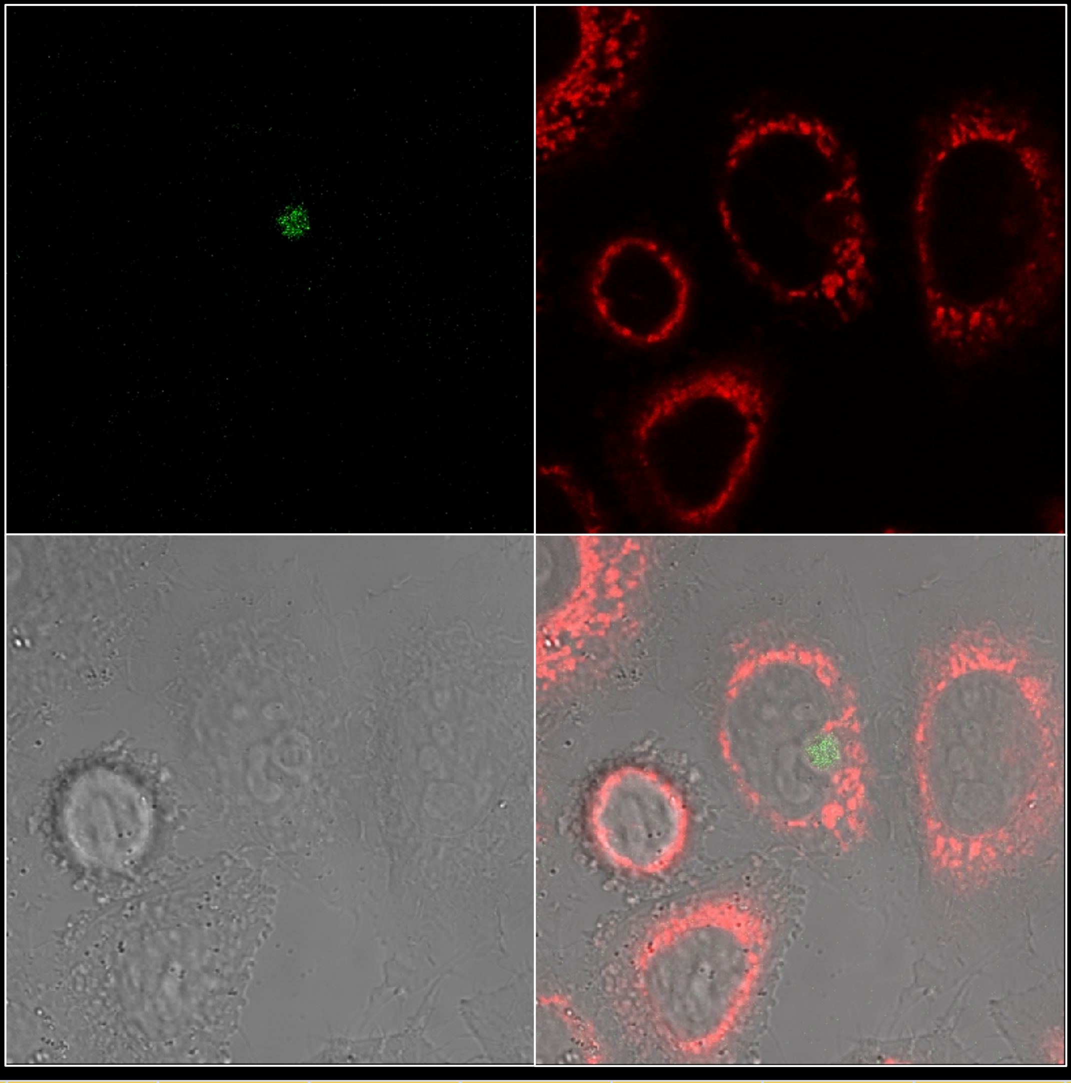

Pearsons Correlation	Colocalization Coefficient Mx	Colocalization Coefficient My	Overlap Coefficient R	Overlap Coefficient Kx	Overlap Coefficient Ky	X Min Threshold	X Max Threshold	Y Min Threshold	Y Max Threshold	Voxel Ratio Ch.X/Ch.Y	Global Pearsons Correlation
-0.06925	0.3435	0.10945	0.13	1.56	0.01115	5	255	26	255	0.2755	0.0426

Colocalization Experiments HeLa

Lact/Malt3-QD (21b)

Golgi

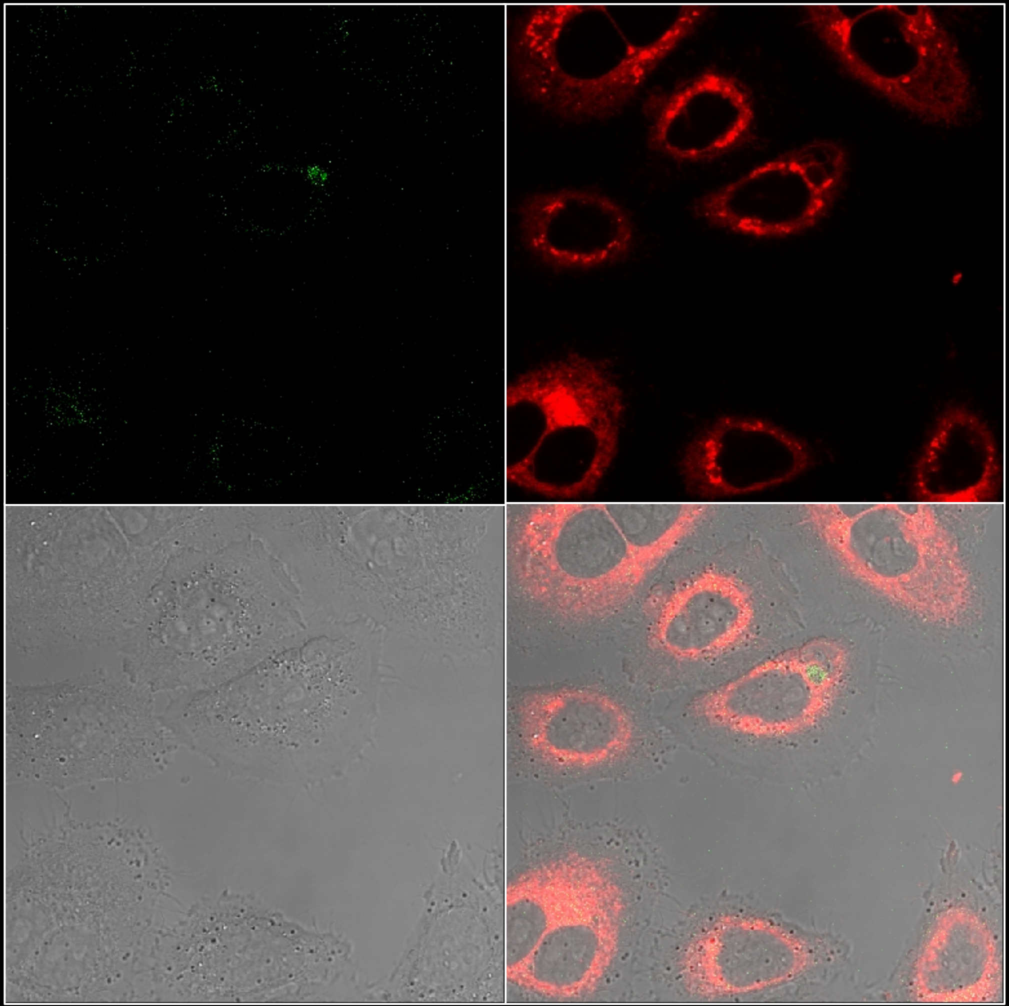

Pearsons Correlation	Colocalization Coefficient Mx	Colocalization Coefficient My	Overlap Coefficient R	Overlap Coefficient Kx	Overlap Coefficient Ky	X Min Threshold	X Max Threshold	Y Min Threshold	Y Max Threshold	Voxel Ratio Ch.X/Ch.Y	Global Pearsons Correlation
0.0144	0.6575	0.2775	0.329	6.07	0.01885	3	255	41	255	0.372	0.211

Colocalization Experiments HeLa

Lact/Malt3-QD (21b)

Golgi

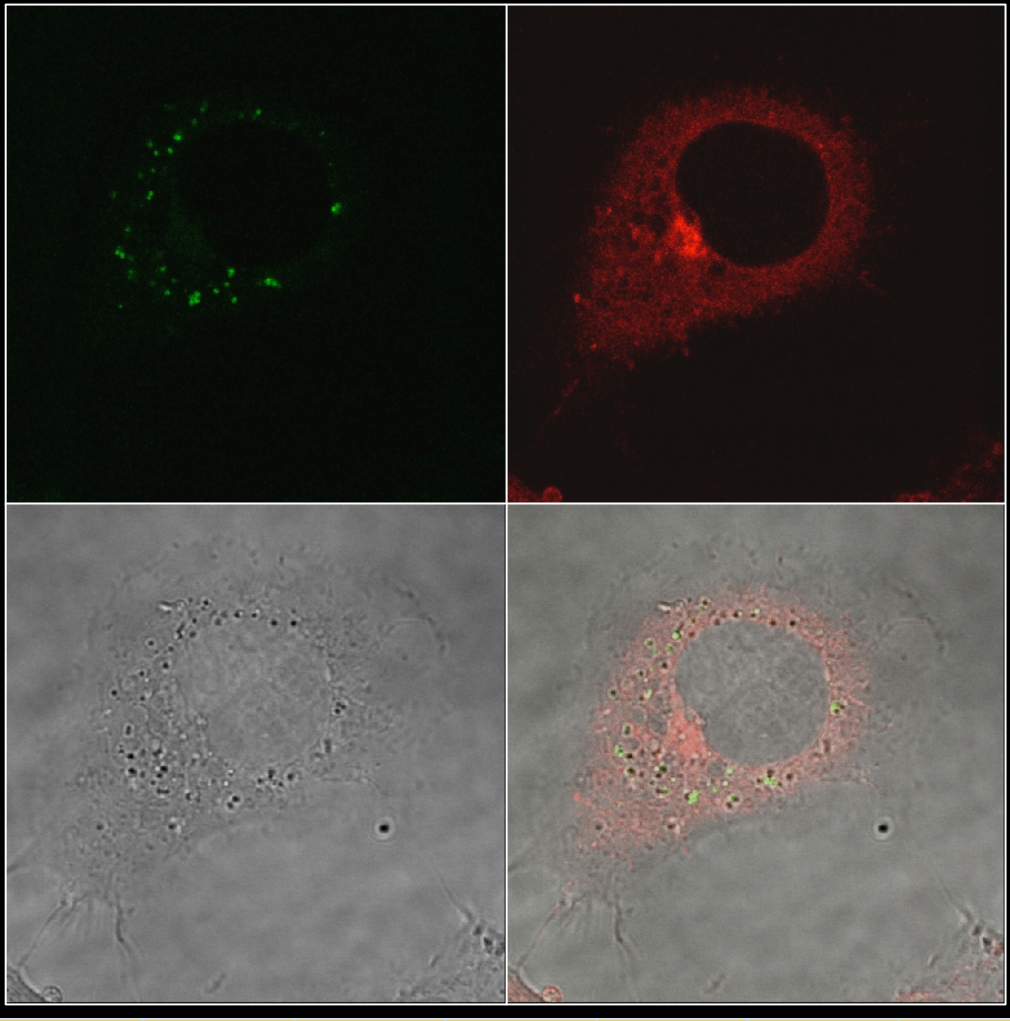

Pearsons Correlation	Colocalization Coefficient Mx	Colocalization Coefficient My	Overlap Coefficient R	Overlap Coefficient Kx	Overlap Coefficient Ky	X Min Threshold	X Max Threshold	Y Min Threshold	Y Max Threshold	Voxel Ratio Ch.X/Ch.Y	Global Pearsons Correlation
-0.0911	0.638	0.15	0.239	0.658	0.0866	12	167	19	167	0.238	0.212

Colocalization Experiments HeLa

Lact/Malt3-QD (21b)

Golgi

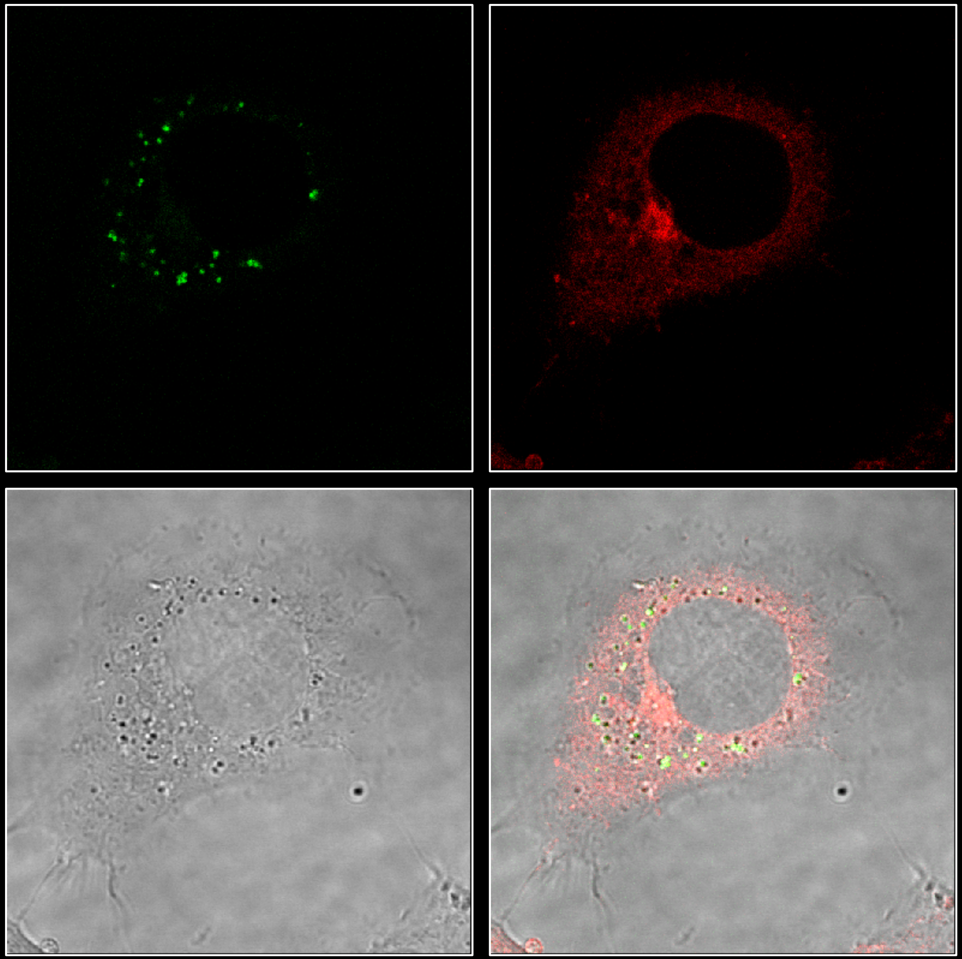

Pearsons Correlation	Colocalization Coefficient Mx	Colocalization Coefficient My	Overlap Coefficient R	Overlap Coefficient Kx	Overlap Coefficient Ky	X Min Threshold	X Max Threshold	Y Min Threshold	Y Max Threshold	Voxel Ratio Ch.X/Ch.Y	Global Pearsons Correlation
0.0144	0.6575	0.2775	0.329	6.07	0.01885	3	255	41	255	0.372	0.211

Colocalization Experiments HeLa

Lact/Malt3-QD (21b)

Mitochondria

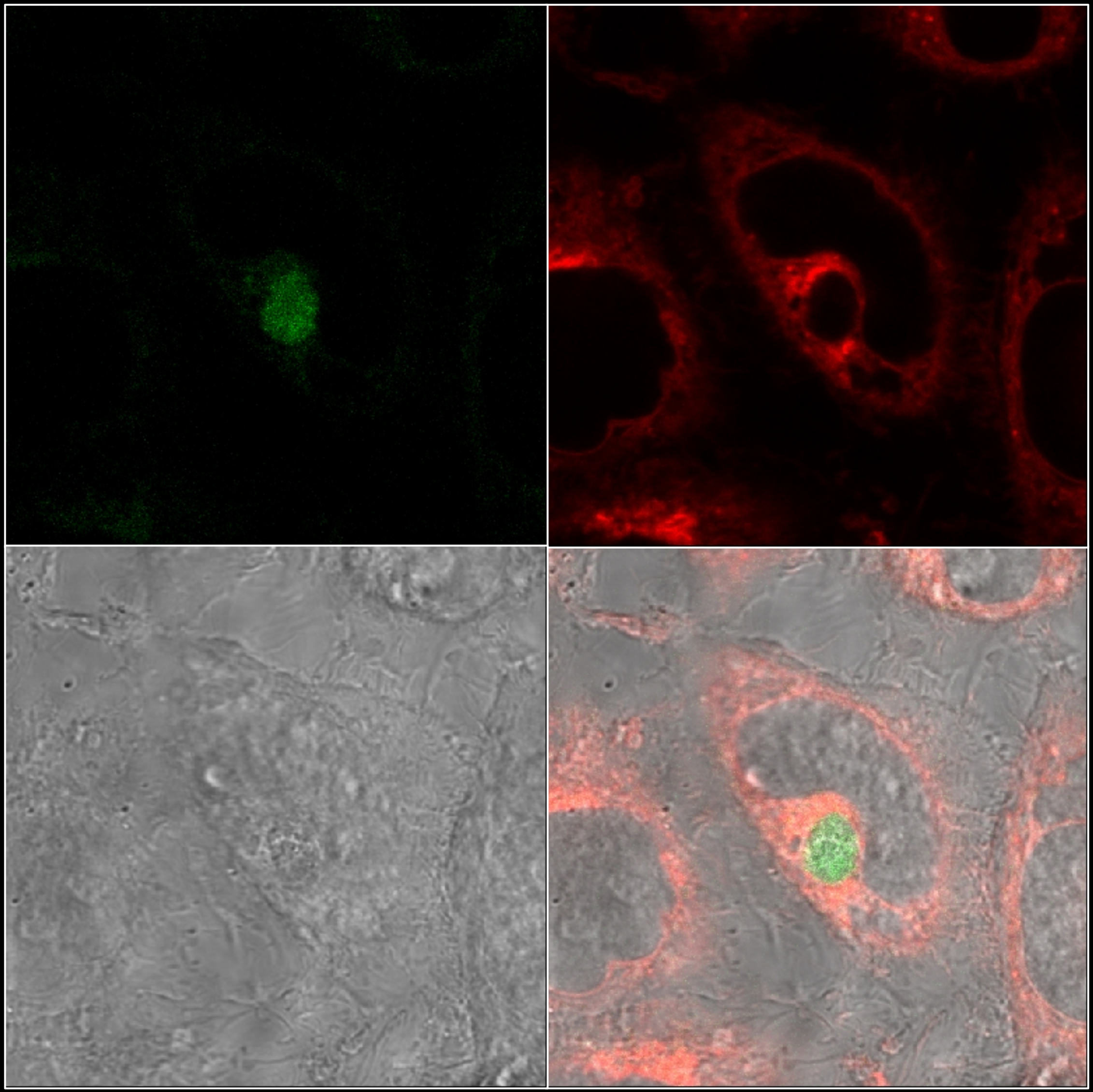

Colocalization Experiments AS

Lact/Malt3-QD (21b)

Early Endosomes

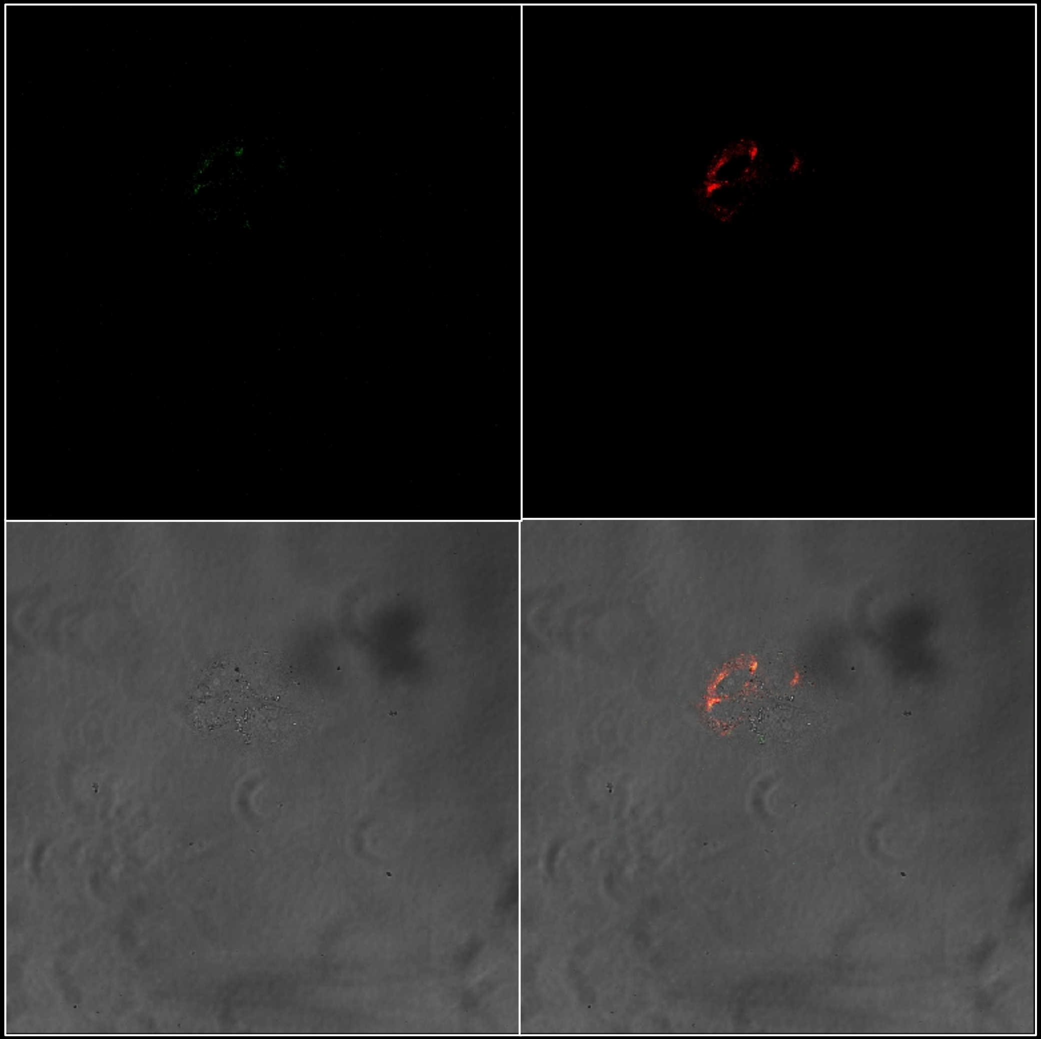

Pearsons Correlation	Colocalization Coefficient Mx	Colocalization Coefficient My	Overlap Coefficient R	Overlap Coefficient Kx	Overlap Coefficient Ky	X Min Threshold	X Max Threshold	Y Min Threshold	Y Max Threshold	Voxel Ratio Ch.X/Ch.Y	Global Pearsons Correlation
0.416	1	1	0.53	1.2745	0.2395	1	255	1	255	1	0.416

Colocalization Experiments AS

Lact/Malt3-QD (21b)

Late Endosomes

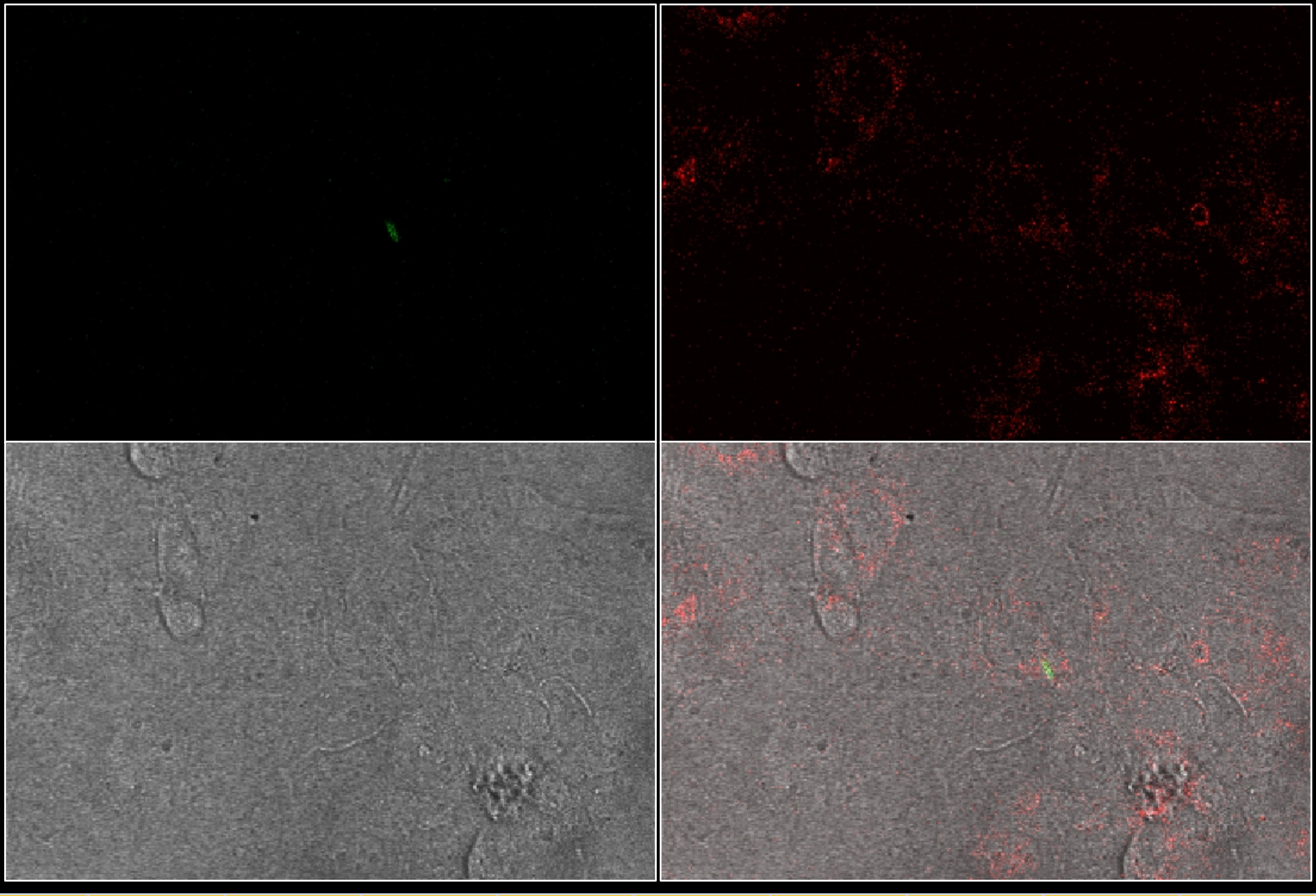

Pearsons Correlation	Colocalization Coefficient Mx	Colocalization Coefficient My	Overlap Coefficient R	Overlap Coefficient Kx	Overlap Coefficient Ky	X Min Threshold	X Max Threshold	Y Min Threshold	Y Max Threshold	Voxel Ratio Ch.X/Ch.Y	Global Pearsons Correlation
0.022	0.2512	0.0602	0.127	0.5	0.035	20	255	21	255	0.227	0.1

Colocalization Experiments AS

Lact/Malt3-QD (21b)

Lysosomes

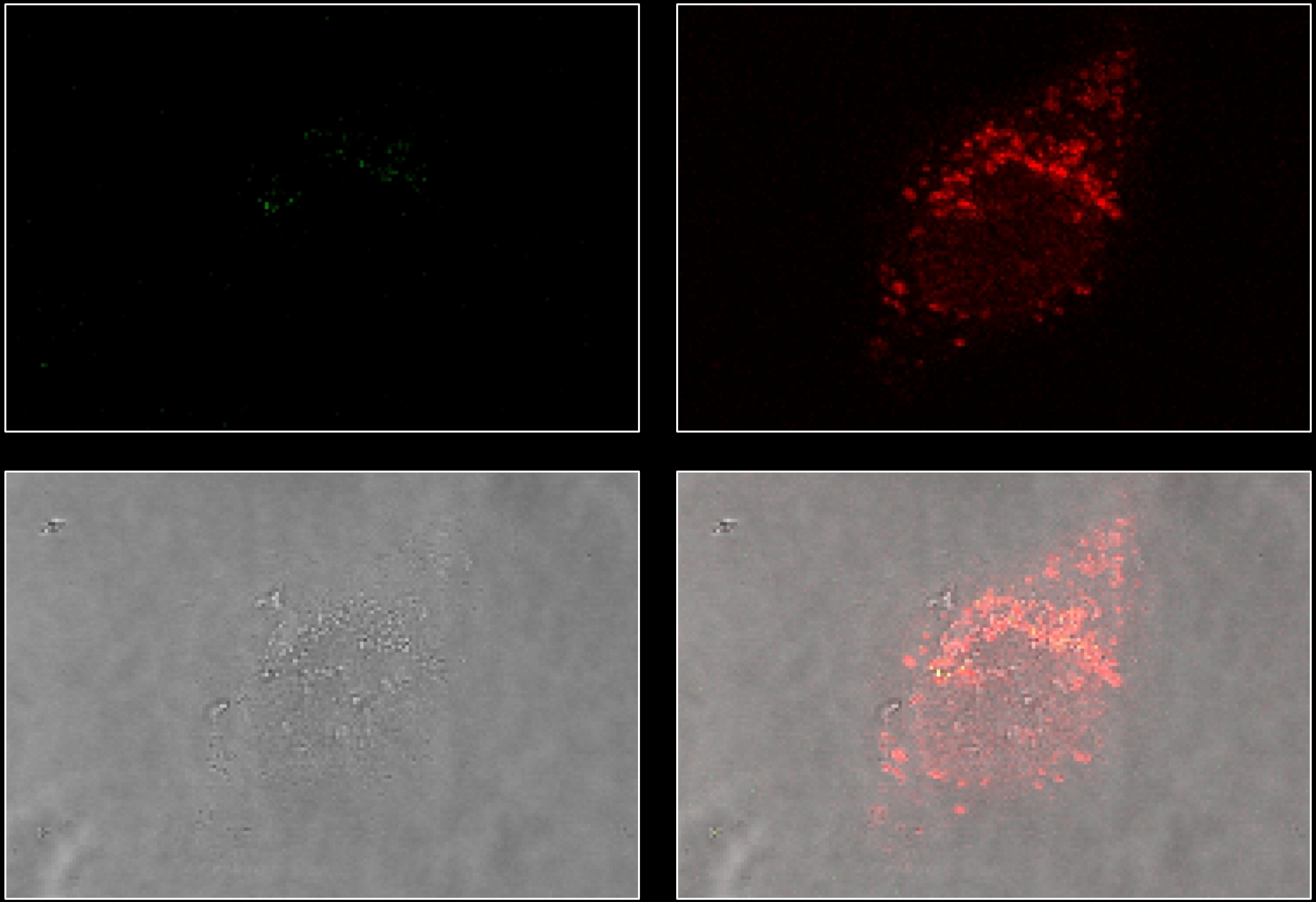

Pearsons Correlation	Colocalization Coefficient Mx	Colocalization Coefficient My	Overlap Coefficient R	Overlap Coefficient Kx	Overlap Coefficient Ky	X Min Threshold	X Max Threshold	Y Min Threshold	Y Max Threshold	Voxel Ratio Ch.X/Ch.Y	Global Pearsons Correlation
-0.0947	0.08675	0.689	0.265	0.0548	1.307	51	255	57	255	11.01	0.2445

Colocalization Experiments AS

LactMalt3-QD (21b)

Lysosomes

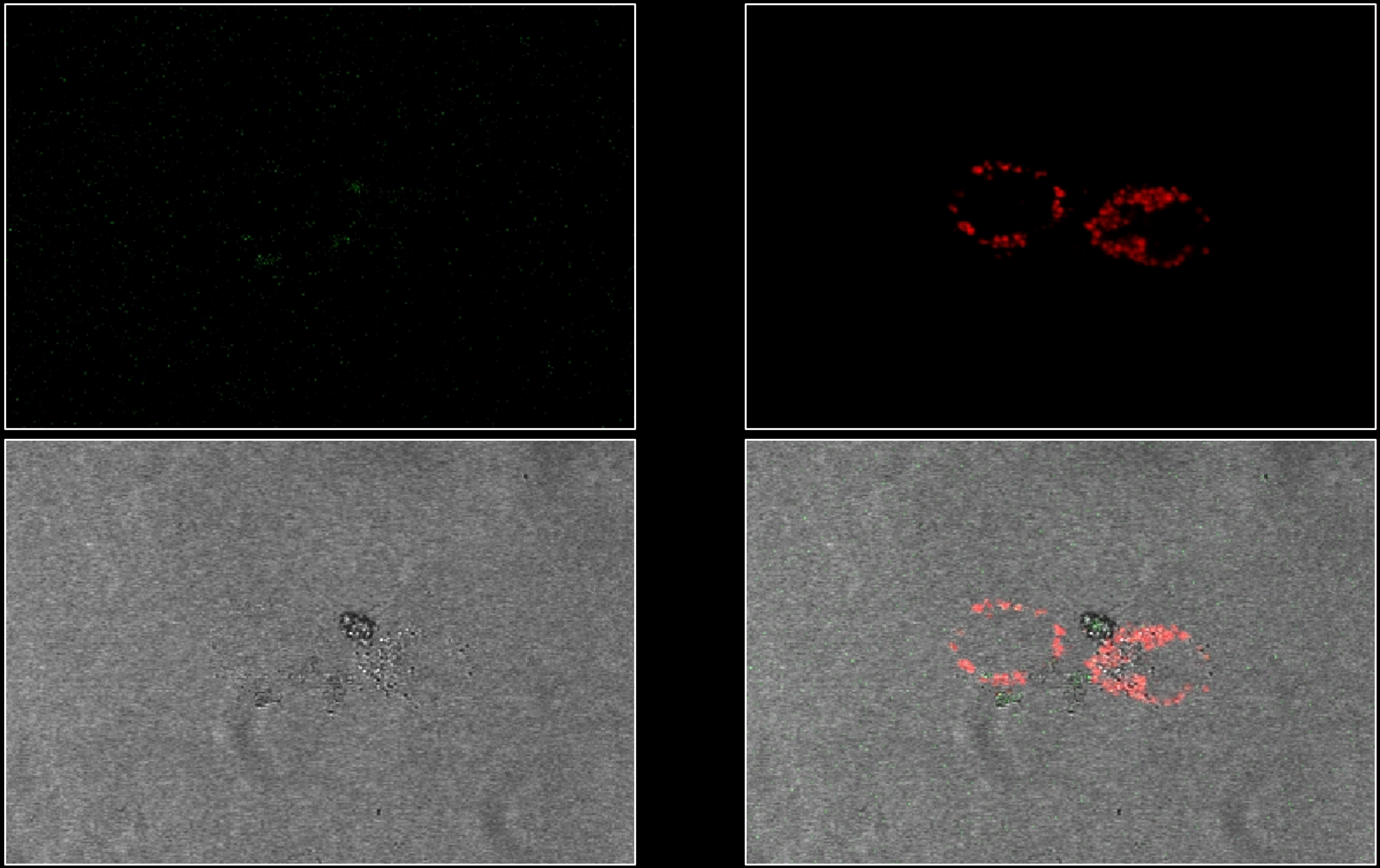

Pearsons Correlation	Colocalization Coefficient Mx	Colocalization Coefficient My	Overlap Coefficient R	Overlap Coefficient Kx	Overlap Coefficient Ky	X Min Threshold	X Max Threshold	Y Min Threshold	Y Max Threshold	Voxel Ratio Ch.X/Ch.Y	Global Pearsons Correlation
-0.2175	0.00862	0.16045	0.1374	0.00372	0.3205	41	255	41	255	18.75	0.0205

Colocalization Experiments AS

Lact/Malt3-QD (21b)

ER

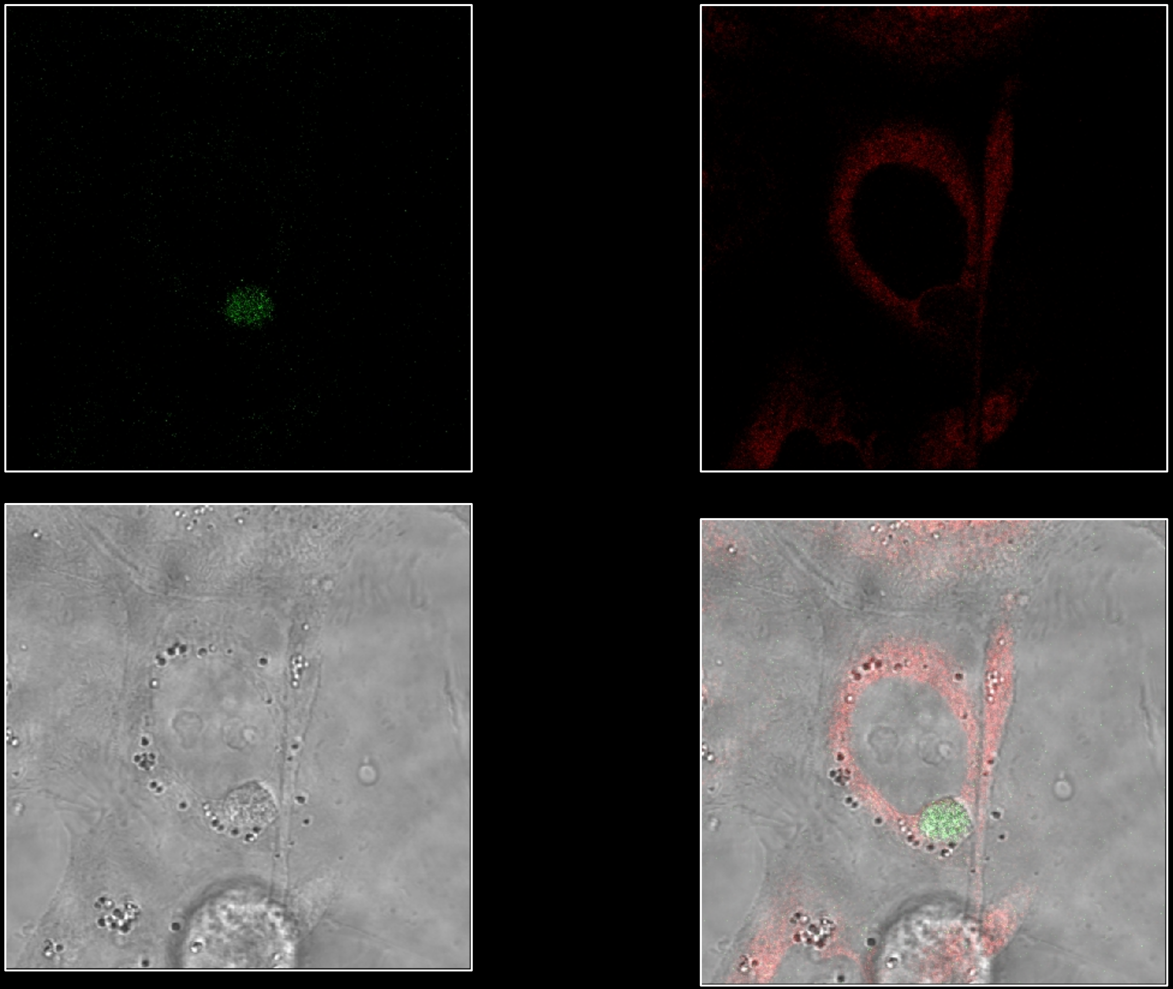

Pearsons Correlation	Colocalization Coefficient Mx	Colocalization Coefficient My	Overlap Coefficient R	Overlap Coefficient Kx	Overlap Coefficient Ky	X Min Threshold	X Max Threshold	Y Min Threshold	Y Max Threshold	Voxel Ratio Ch.X/Ch.Y	Global Pearsons Correlation
-0.102	0.05625	0.133	0.0708	0.15385	0.04125	8	128	27	128	3.015	0.0034

Colocalization Experiments AS

Lact/Malt3-QD (21b)

ER

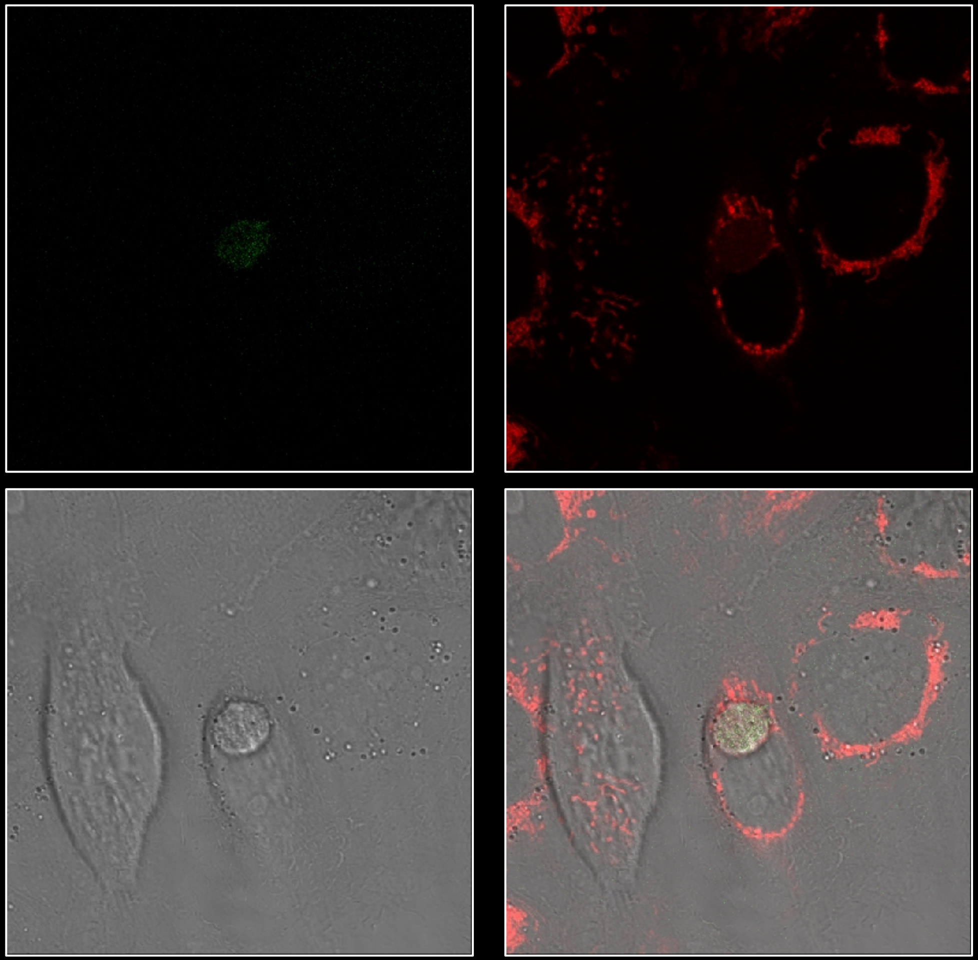

Pearsons Correlation	Colocalization Coefficient Mx	Colocalization Coefficient My	Overlap Coefficient R	Overlap Coefficient Kx	Overlap Coefficient Ky	X Min Threshold	X Max Threshold	Y Min Threshold	Y Max Threshold	Voxel Ratio Ch.X/Ch.Y	Global Pearsons Correlation
-0.07375	0.5245	0.2195	0.2155	1.0725	0.0442	5	255	12	255	0.52	0.1121

Colocalization Experiments AS

Lact/Malt3-QD (21b)

Golgi

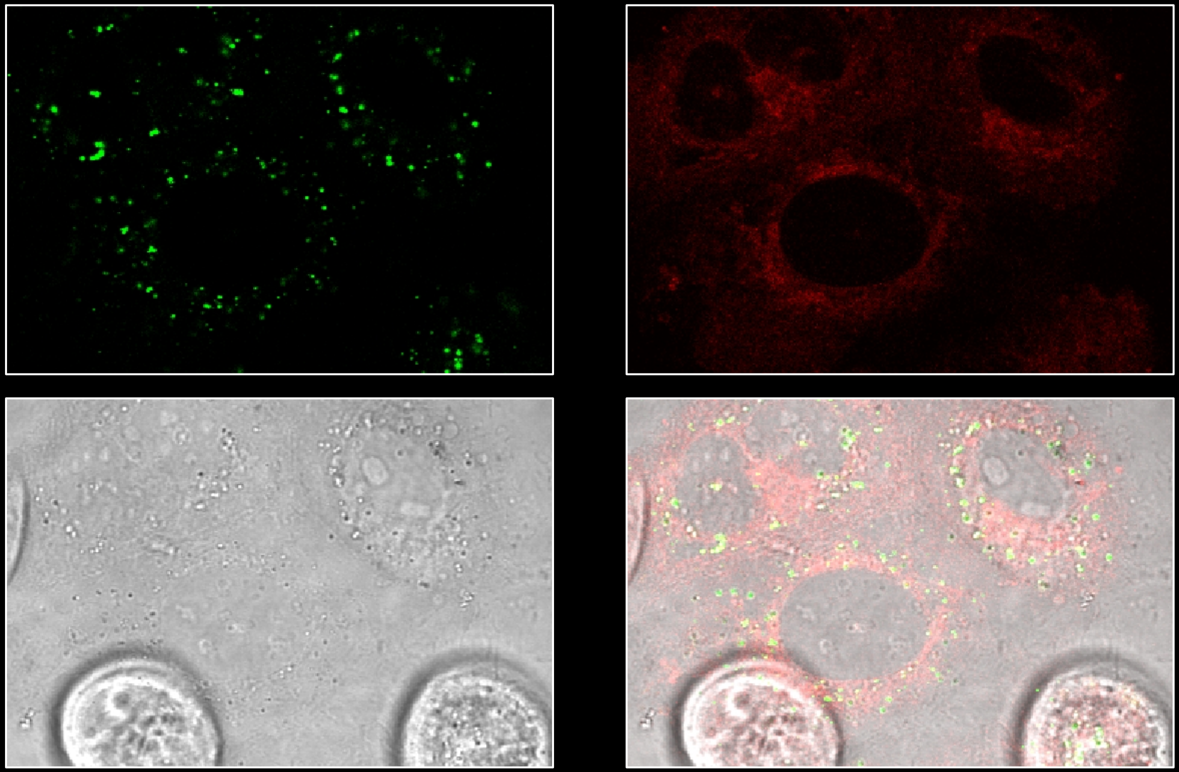

Pearsons Correlation	Colocalization Coefficient Mx	Colocalization Coefficient My	Overlap Coefficient R	Overlap Coefficient Kx	Overlap Coefficient Ky	X Min Threshold	X Max Threshold	Y Min Threshold	Y Max Threshold	Voxel Ratio Ch.X/Ch.Y	Global Pearsons Correlation
0.167	0.805	1	0.4705	0.433	0.514	5	255	7	255	1.63	0.2113

Colocalization Experiments AS

LactMalt3-QD (21b)

Golgi

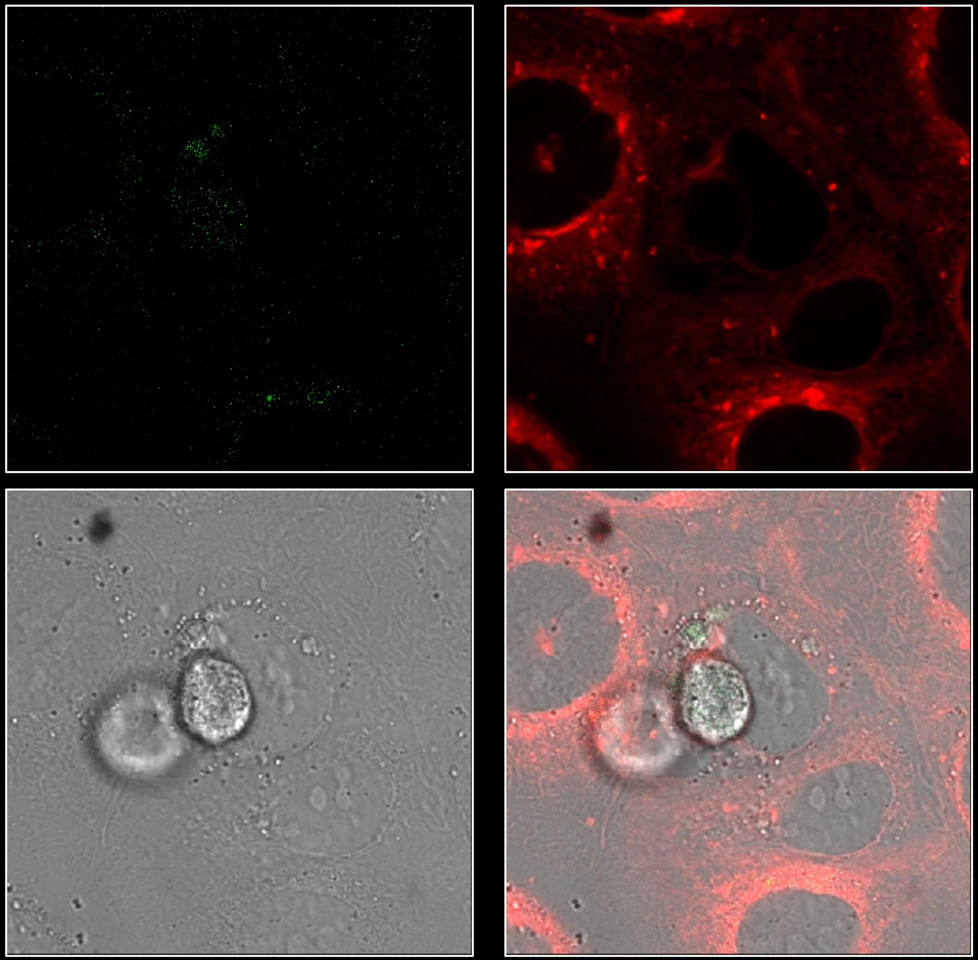

Pearsons Correlation	Colocalization Coefficient Mx	Colocalization Coefficient My	Overlap Coefficient R	Overlap Coefficient Kx	Overlap Coefficient Ky	X Min Threshold	X Max Threshold	Y Min Threshold	Y Max Threshold	Voxel Ratio Ch.X/Ch.Y	Global Pearsons Correlation
0.1097	0.25	0.193	0.2015	1.5225	0.0281	9	255	58	255	0.8	0.12315

Colocalization Experiments AS

Lact/Malt3-QD (21b)

Mitochondria

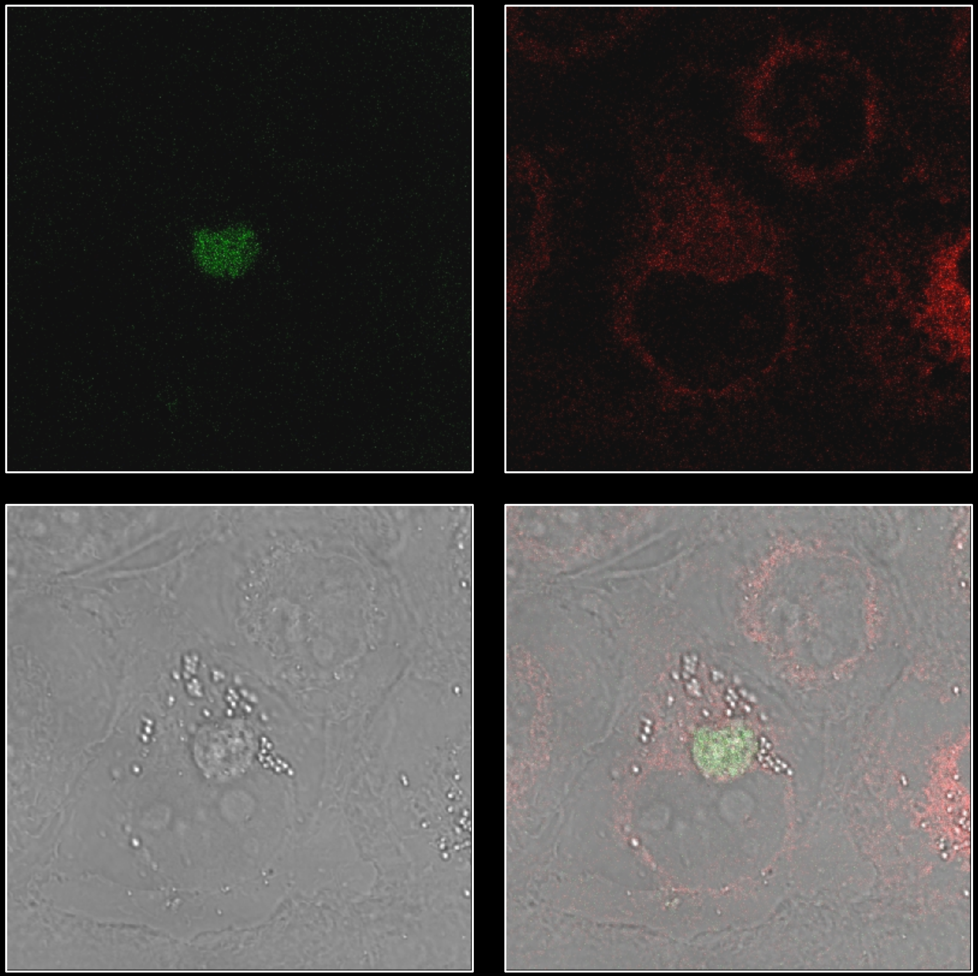

Pearsons Correlation	Colocalization Coefficient Mx	Colocalization Coefficient My	Overlap Coefficient R	Overlap Coefficient Kx	Overlap Coefficient Ky	X Min Threshold	X Max Threshold	Y Min Threshold	Y Max Threshold	Voxel Ratio Ch.X/Ch.Y	Global Pearsons Correlation
-0.02715	0.3595	0.10215	0.15515	0.3035	0.0924	11	134	13	134	0.267	0.05645

Colocalization Experiments AS

Lact/Malt3-QD (21b)

Mitochondria

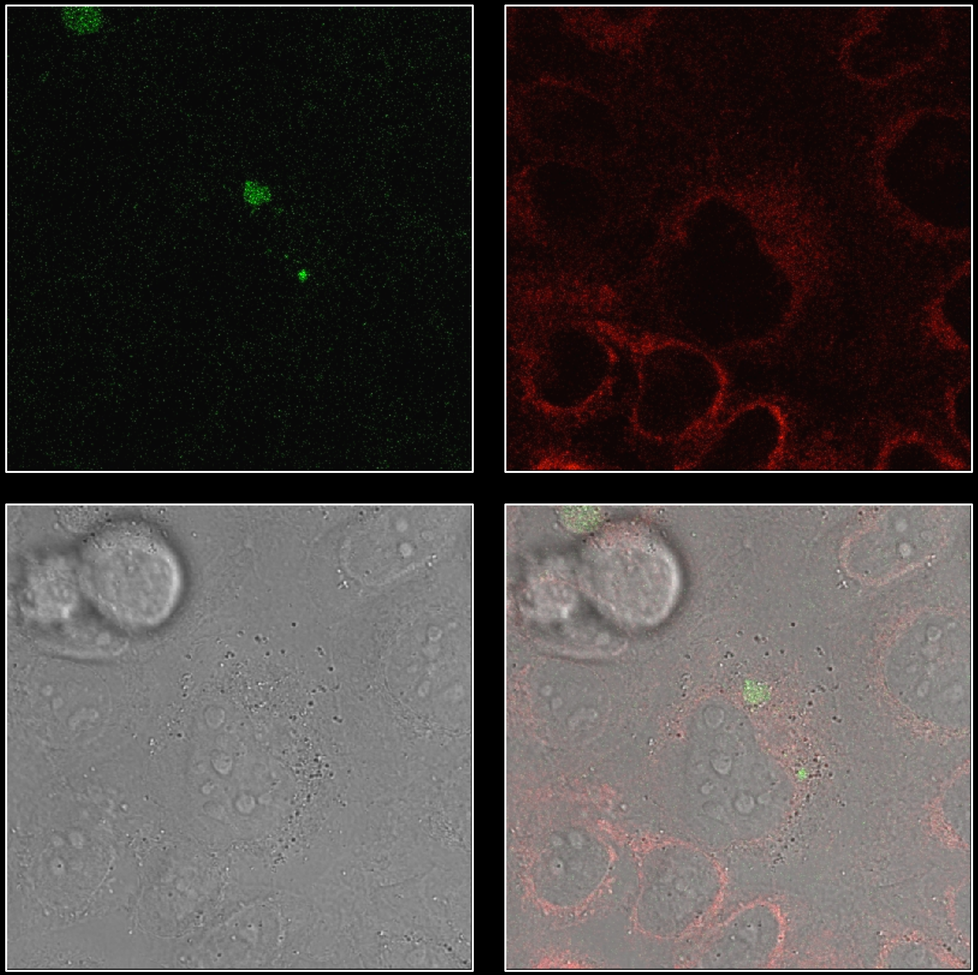

Pearsons Correlation	Colocalization Coefficient Mx	Colocalization Coefficient My	Overlap Coefficient R	Overlap Coefficient Kx	Overlap Coefficient Ky	X Min Threshold	X Max Threshold	Y Min Threshold	Y Max Threshold	Voxel Ratio Ch.X/Ch.Y	Global Pearsons Correlation
0.0553	0.421666667	0.158866667	0.2167	0.353	0.1503	10	117	12	117	0.351	0.061233333
